# Thioesters Support Efficient Protein Biosynthesis by the Ribosome

**DOI:** 10.1021/acscentsci.4c01698

**Published:** 2025-01-30

**Authors:** Alexandra D. Kent, Jacob G. Robins, Isaac J. Knudson, Jessica T. Vance, Alexander C. Solivan, Noah X. Hamlish, Katelyn A. Fitzgerald, Alanna Schepartz, Scott J. Miller, Jamie H. D. Cate

**Affiliations:** † Department of Chemistry, University of California, Berkeley, California 94720, United States; ‡ Department of Chemistry, Yale University, New Haven, Connecticut 06520, United States; § Department of Molecular and Cell Biology, University of California, Berkeley, California 94720, United States; ∥ Molecular Biophysics and Integrated Bioimaging Division, Lawrence Berkeley National Laboratory, Berkeley, California 94270, United States; ⊥ Chan Zuckerberg Biohub, San Francisco, California 94158, United States; # Innovative Genomics Institute, University of California, Berkeley, California 94720, United States

## Abstract

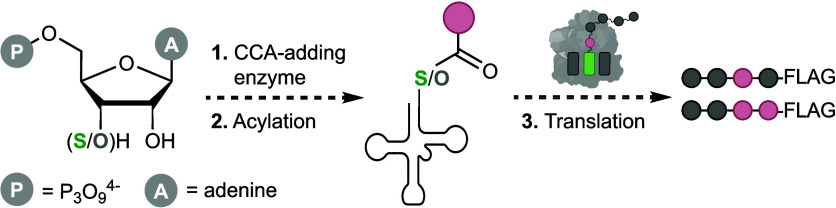

Thioesters are critical chemical intermediates in numerous extant biochemical reactions and are invoked as key reagents during prebiotic peptide synthesis on an evolving Earth. Here we asked if a thioester could replace the native oxo-ester in acyl-tRNA substrates during protein biosynthesis by the ribosome. We prepared 3′-thio-3′-deoxyadenosine triphosphate in 10 steps from xylose and demonstrated that it is an effective substrate for the *Escherichia coli* CCA-adding enzyme, which appends 3′-thio-3′-deoxyadenosine to truncated tRNAs ending with 3′-CC. Using a variety of aminoacyl-tRNA synthetases, flexizymes, or a direct thioester exchange reaction, we prepared a suite of 3′-thio-tRNAs acylated with α- and non-α-amino acids. All were recognized and utilized by wild-type *E. coli* ribosomes during *in vitro* translation reactions to generate oligopeptides in yields commensurate with native oxo-ester tRNAs. These results indicate that thioester intermediates widely used in Nature can be co-opted to support the incorporation of natural α-amino acids as well as noncanonical monomers by the extant translational machinery for sequence-defined polymer synthesis.

## Abstract 2

We show that tRNAs acylated with a thioester rather than an ester linkage to natural and unnatural monomers can be used by the extant translation machinery for sequence-defined polymer synthesis.

## Introduction

Thioesters are ubiquitous and well-studied reactive species in biosynthesis. For example, the active sites of polyketide synthases and nonribosomal peptide synthetases leverage enhanced thioester reactivity to promote challenging condensation reactions that form new C–N, C–O and C–C bonds. The markedly higher reaction rates of thioesters compared to oxo-esters with various nucleophiles has been rationalized by electron delocalization effects.^1−3^ By contrast, all extant ribosomes employ oxo-esters to support bond forming reactions, despite the observation that thioesters are generally more reactive toward amine nucleophiles.^1,2^ During translation, thioesters could in principle substitute for oxo-esters if tRNA substrates carried an SH in place of OH on the 3′-terminal ribose. However, substituting a sulfur for an oxygen atom in an acyl-tRNA could impact translation positively or negatively at multiple steps during, before, or after the translation cycle. Although thioesters are more reactive toward amine nucleophiles than oxo-esters,^1,4^ C–S bonds are longer than C–O bonds, and S atoms more polarizable than O atoms; thiols and thioethers donate and accept H-bonds differently than alcohols,^5−7^ making the overall effect of sulfur substitution on ribosomal protein synthesis challenging to predict. Steps in translation that could be affected by these differences include tRNA maturation, in which the tRNA 3′-terminal CCA nucleotides are added, tRNA aminoacylation, tRNA delivery to the ribosome during mRNA decoding, and finally peptide bond formation in the ribosomal active site, the peptidyl transferase center (PTC).^8^

Many have theorized that thioesters may have played a key role in the origin of life,^9−12^ wherein aminoacyl thioesters^13−15^ could have generated oligomeric peptides with potential catalytic activity,^10^ thereby acting as a link between the prebiotic and RNA world.^16,17^ Prebiotically plausible synthetic routes to nucleosides with 2′-thiol-modified ribose have been identified and routes to 3′-thiol-modifications proposed, further strengthening the possibility that short sequences of modified RNAs could have acted as early amino acid transfer reagents as the first RNA proto-ribosomes evolved.^9,13,15^ Thus, despite modern peptidyl transfer in the ribosome using exclusively oxo-ester-linked aminoacyl-tRNA, it is possible that thioesters rather than oxo-esters acted as early intermediates in ribosomal peptide synthesis.^18,19^

**Figure 1:**
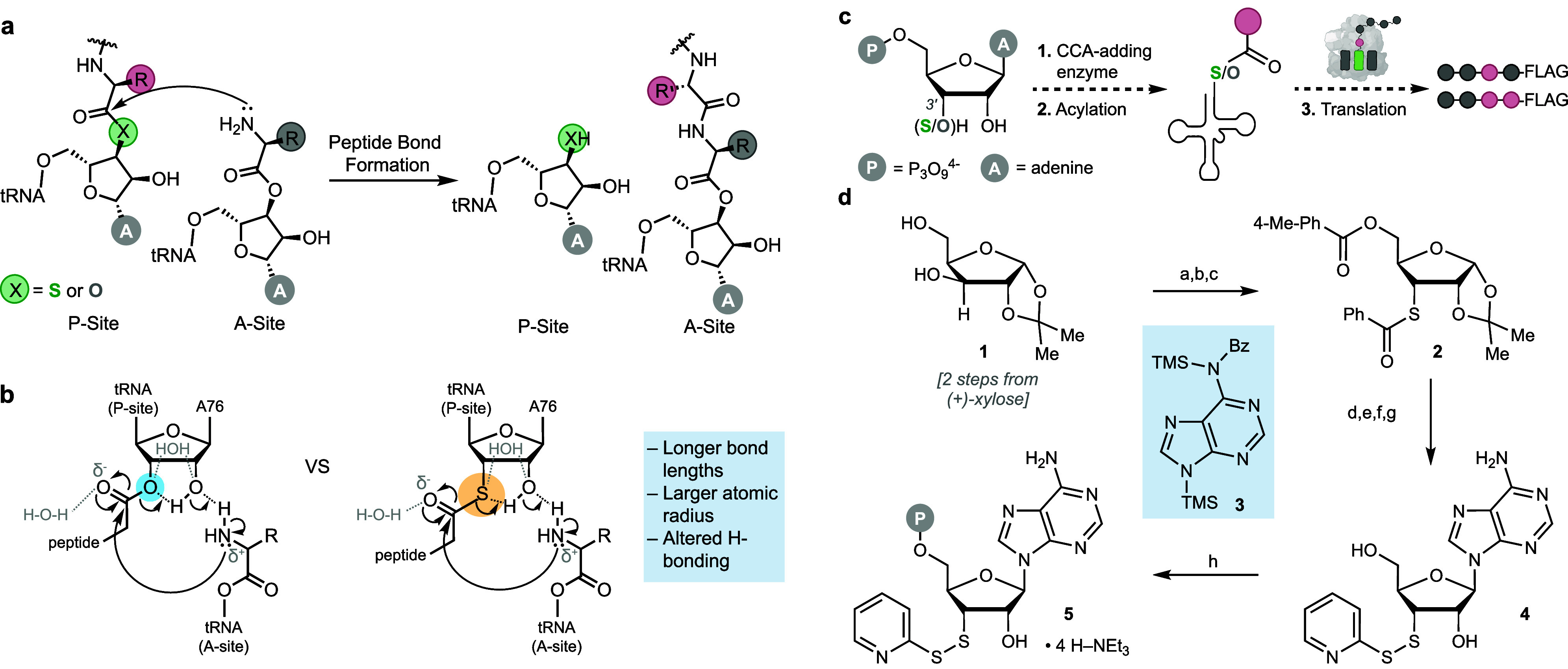
**Design and synthesis of 3**′**-thio-tRNAs. a**, Peptide bond formation within the ribosomal PTC involves attack of a nucleophile appended to an A site tRNA with an electrophile appended to the P-site tRNA. The P-site electrophile is an oxo-ester in native tRNAs, but is replaced by a thioester in the experiments described herein. **b**, Reactivity of 3′-thioester in the PTC. Relevant effects of the oxygen to substitution are highlighted. **c**, Schematic showing the three enzymatic reactions in which thioester replacement was evaluated. **d**, Synthesis of nucleotide triphosphate **5**: (a) 4-Me-PhCOCl (1.0 equiv), pyridine, 0 °C, 1 h, 56% yield; (b) Tf_2_O (2.3 equiv), DMAP (2.55 equiv), CH_2_Cl_2_, 0 °C, 45 min.; (c) NaH (3.15 equiv), thiobenzoic acid (3.0 equiv), DMF, 0–60 °C, 5 h, 35% over 2 steps; (d) HCO_2_H, 50 °C, 1 h; (e) Ac_2_O, pyridine, rt, 2 h, 65% yield over 2 steps; (f) TMSOTf (1.5 equiv), nucleobase **3** (1.2 equiv), DCE, 85 °C, 5 h, 21% yield; (g) NH_4_OH (aq.), 23 °C, 24 h, then (2-pyridyl-S)_2_ (1.5 equiv), MeOH, 88% yield; (h) POCl_3_ (1.2 equiv), PO(OMe)_3_, −10 °C, 2 h; then (*n*-Bu_3_NH_2_)_2_(H_2_P_2_O_7_) (3.0 equiv), *n*-Bu_3_N (6.0 equiv), DMF, −10 °C, 0.5 h; then Et_3_N·H_2_CO_3_ (aq.), 23 °C, 1 h, 33% yield.

Here we explored whether thiolated tRNA molecules, bearing only a single atom change from native tRNA,^19^ could serve as acyl donors by the extant translational machinery (Figure 1a,b).^18^ We replaced the 3′-OH on the 3′-terminal adenosine of a tRNA with a 3′-SH and evaluated its performance in three critical steps during translation (Figure 1c). As no 2′- or 3′-modified tRNA has previously been shown to be active in translation, we asked if *S*-acyl tRNAs would be tolerated, and if so, how the chemical differences between oxo-esters and thioesters would manifest with respect to translation and specificity. We synthesized 3′-thio-3′-deoxyadenosine triphosphate and tested whether a 3′-thio-3′-deoxyribose could function in three key steps of translation: tRNA extension using CCA-adding enzymes, aminoacylation, and finally ribosome-mediated peptide bond formation to introduce canonical and noncanonical monomers (Figure 1c). We show that all three biochemical reactions are fully supported by thioesters, with yields comparable to oxo-ester tRNA.

## Results

### Synthesis of 3′-Thio-3′-deoxyadenosine Triphosphate

These studies began with the synthesis of 3′-thio-3′-deoxyadenosine from readily available (+)-xylose (Figure 1d).^20^ Selective primary alcohol protection of diol **1** enabled triflation and substitution to provide thiobenzoate **2** as the desired thiolate epimer. We installed the nucleobase via Vorbrüggen glycosylation using bis-silylated nucleobase **3**.^21^ After global deprotection and disulfide exchange, we obtained pyridyl disulfide **4** as a bench-stable solid. To avoid further protecting group manipulations, we deployed Ludwig-Yoshikawa triphosphorylation conditions to generate disulfide-protected nucleotide triphosphate **5** as the tetrakis(triethylammonium) salt following ion-exchange chromatography.^22^

### Addition of 3′-Thio-3′-deoxyadenosine to 3′-Truncated tRNAs

We next tested whether 3′-thio-ATP **5** is a substrate for *E. coli* tRNA nucleotidyltransferase, a CCA-adding enzyme, to add 3′-thio-3′-deoxyadenosine to the 3′-end of truncated tRNAs.^23^ While used canonically by cells to add the terminal 3′-CCA to 3′-truncated tRNAs during tRNA biosynthesis, CCA-adding enzymes also append adenosine analogs to 3′-truncated tRNAs *in vitro*.^24,25^ The substrate scope of CCA-adding enzymes includes modified bases (N^6^-methyladenosine, diaminopurine), but only one 3′-modified substrate has been reported to date, 3′-amino-3′-deoxy-adenosine.^26^ The product of this reaction, 3′-amino-tRNA has found widespread use as a substrate analogue for structural studies of the ribosome,^27−29^ but because its acylated form is an amide, not an ester, it cannot support translation.

**Figure 2:**
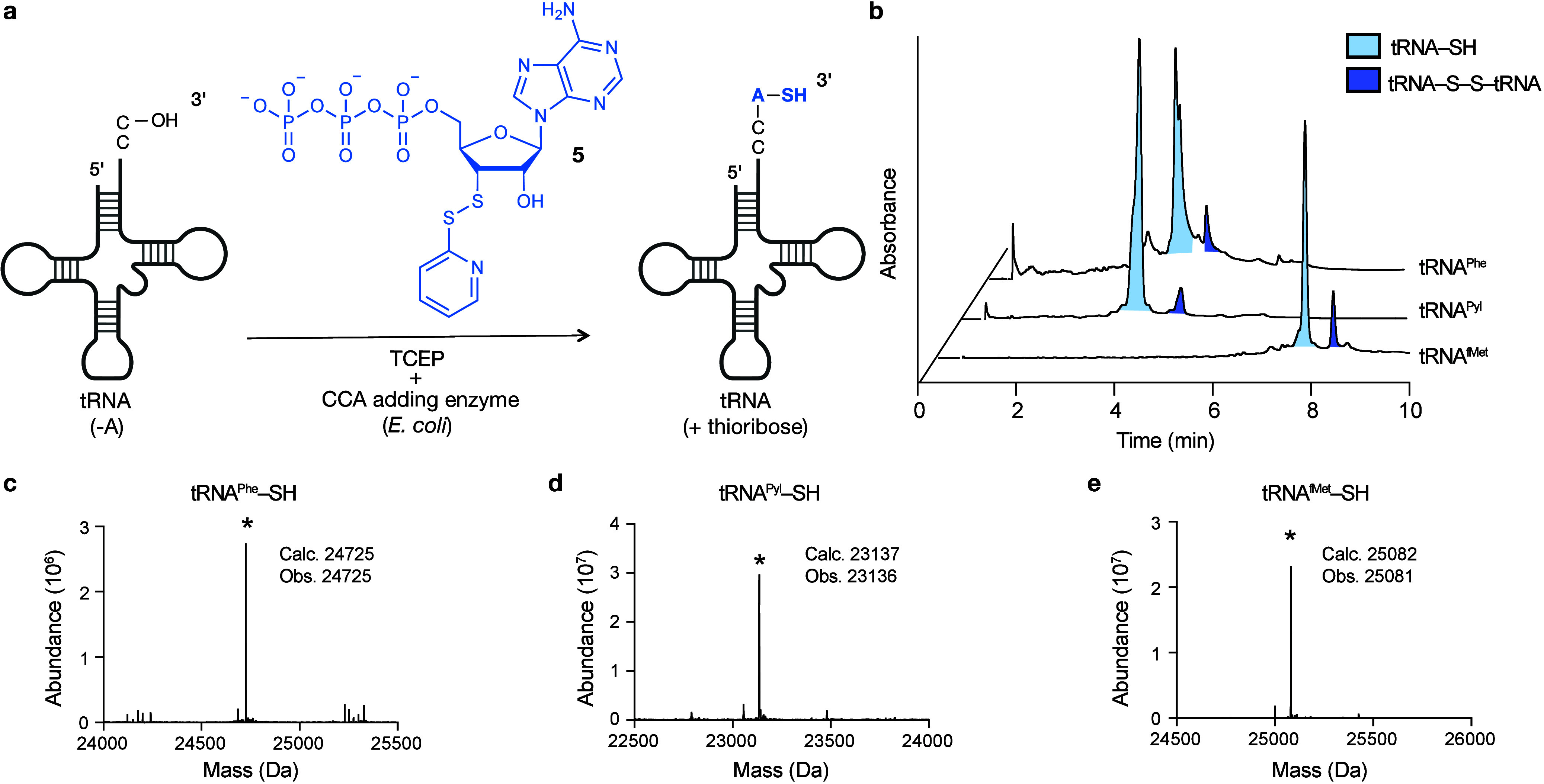
***E. coli*CCA-adding enzyme adds 3′-thio-ATP 5 to the 3′-end of multiple 3′-truncated tRNAs. a**, Reaction scheme illustrating addition of 3′-thio-ATP **5** to a 3**′**-truncated tRNA (tRNA (-A). TCEP acts as a reducing agent to remove the thiopyridyl disulfide protecting group from 3′-thio-ATP (**5**) before it is added to the 3′-end of a 3′-truncated tRNA by a CCA-adding enzyme, generating a 3′-thio-tRNA. See Methods for details. **b**, Intact LC-MS of the products resulting from treatment of 3′-truncated tRNA^fMet^, tRNA^Pyl^, and tRNA^Phe^ with **5**, TCEP, and *E. coli* CCA-adding enzyme. Light blue peaks correspond to the desired 3′-thio tRNA (tRNA-SH) products. Dark blue peaks correspond to two 3′-thio-tRNAs linked through a disulfide bond (tRNA-S-S-tRNA). **c–e**, MS spectra of intact tRNAs. Starred peaks correspond to the expected tRNA-SH product.

Using in vitro transcription, we generated a series of 3′-truncated tRNAs that lacked the 3′-adenosine–(tRNA^fMet^(-A), tRNA^Pyl^(-A), and tRNA^Phe^(-A) (Figure S6 and S7) and treated them with *E. coli* CCA-adding enzyme in the presence of 3′-thio-ATP **5** and a reducing agent, tris(2-carboxy)phosphine (TCEP) (Figure 2a). We analyzed the reaction products using intact tRNA liquid chromatography–mass spectrometry (LC-MS),^30^ revealing quantitative conversion of the 3′-truncated tRNAs into full-length 3′-thio-tRNAs (Figure 2b-e, S8). The major LC peak (light blue) corresponds to the full-length 3′-thio-tRNA (tRNA-SH), while the minor peak (dark blue) results from two 3′-thio-tRNAs being linked through a disulfide bond (tRNA-S-S-tRNA). In these reactions, we ensured that no contaminating ATP was present (Methods), as ATP contamination precluded 3′-thio-tRNA formation. These results show that the *E. coli* CCA-adding enzyme accepts 3′-thio-ATP **5** as a substrate and, using standard reaction conditions, appends this modified nucleotide to the 3′-end of multiple different truncated tRNA molecules *in vitro*.

### Aminoacylation of 3′-Thio-tRNAs

**Figure 3:**
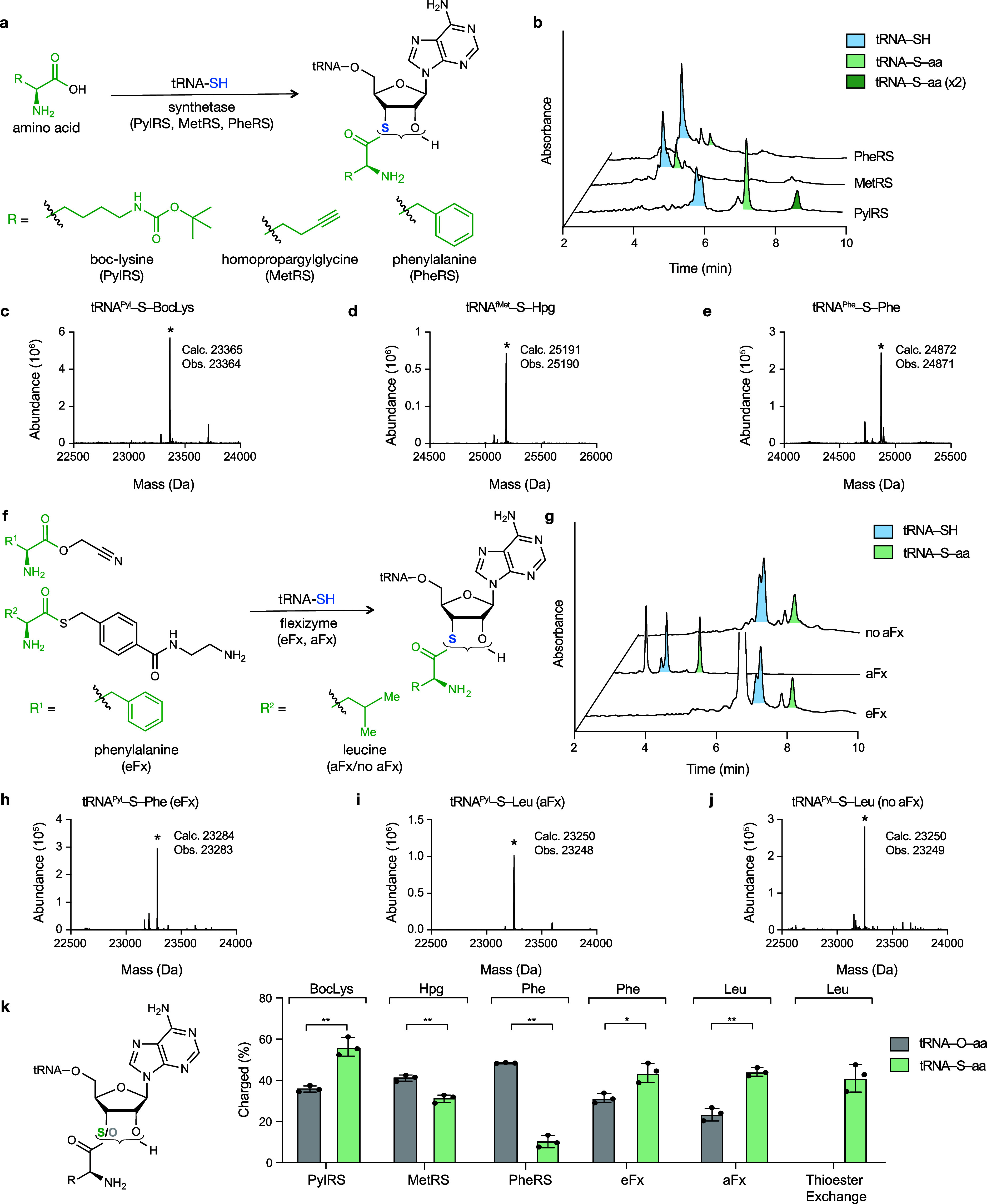
**3′-Thio-tRNAs can be acylated with aminoacyl tRNA synthetases, flexizymes, or chemically to produce thioester linkages. a**, Schematic showing the aminoacyl tRNA synthetase-catalyzed acylation of 3′-thio-tRNA with a canonical or noncanonical α-amino acid. The structures of the α-amino acids are shown and the synthetase used for each is indicated. **b**, LC-MS analysis of acylation reactions with each trace identified by the aaRS used in the reaction. The absorbance trace reveals the presence of varying amounts of tRNA-SH starting material (light blue), monoacyl tRNA, (tRNA-S-aa, light green), and, in the case of BocLys, diacylated tRNA (tRNA-S-aa (x2), dark green). **c–e**, MS spectra of the light green, tRNA-S-aa peak. Masses corresponding to the monoacylated products are starred. **f**, Schematic showing the flexizyme-promoted acylation of 3′-thio-tRNA by the activated esters shown. Amino acid side chains are shown below and the corresponding flexizymes are indicated. **g**, LC-MS analysis of acylation reactions promoted by flexizymes and by thioester exchange in the absence of flexizyme. The first uncolored peak corresponds to eFx in the tRNA^Pyl^-S-Phe sample and aFx in the tRNA^Pyl^-S-Leu sample. The light blue peak corresponds to the tRNA-SH starting material, and the light green peak corresponds to the monoacylated tRNA-S-aa. **h–j**, Deconvoluted mass spectra of the indicated monoacylated tRNA-S-aa product. The calculated and observed (*) masses for each monoacyl tRNA-S-aa are shown. **k**, Relative acylation efficiency of the indicated oxo-ester (gray) and thio-ester (light green) tRNAs as quantified from LC-MS data.

We next evaluated whether aminoacyl tRNA synthetases could acylate 3′-thio-tRNAs with natural or unnatural amino acids. We used *E. coli* phenylalanine-tRNA synthetase (PheRS) to acylate 3′-thio-tRNA^Phe^ with phenylalanine (Phe), and *E. coli* methionine-tRNA synthetase (MetRS) and *M. alvus* pyrrolysine-tRNA synthetase (PylRS) to acylate 3′-thio-tRNA^fMet^ and 3′-thio-tRNA^Pyl^ with the unnatural amino acids homopropargylglycine (Hpg) and *N*_ε_-boc-l-lysine (BocLys), respectively (Figure 3a). All three synthetases acylated the 3′-thio-tRNA, as quantified using intact tRNA LC-MS (Figure 3b-e, S9–14). It has been reported that PylRS can acylate tRNA twice to generate diacylated tRNAs with certain monomers,^30,31^ a phenomenon we also observed when we acylated 3′-thio-tRNA^Pyl^ with BocLys, obtaining a diacylated 3′-thio-tRNA.

In an effort to expand the diversity of monomers that could be used to acylate 3′-thio-tRNA, we also explored whether 3′-thio-tRNA is a substrate for flexizyme-promoted acylation reactions (Figure 3f). We found that eFx promoted the acylation of 3′-thio-tRNA^Pyl^ with cyanomethyl ester-activated Phe (CME-Phe) (Figure 3g,h, S15,16), and that aFx^32^ promoted the acylation of 3′-thio-tRNA^Pyl^ with an amino-derivatized benzyl thioester-activated leucine (ABT-Leu) (Figure 3g,i S17,18) using standard flexizyme reaction conditions. Because the leaving group of ABT-Leu contains a thioester, we hypothesized that it could undergo thioester exchange with 3′-thio-tRNA^Pyl^ even in the absence of a flexizyme. Indeed, addition of ABT-Leu to 3′-thio-tRNA^Pyl^ without aFx produced monoacylated 3′-thio-tRNA^Pyl^ under the same reaction conditions with similar yield. Thus, thioester exchange reactions of 3′-thio-tRNA provide a new, flexizyme-independent method to generate acylated tRNAs (Figure 3g,j, S19).

Additionally, we compared the acylation efficiencies of 3′-thio-tRNAs to those of canonical 3′-tRNAs (Figure 3k). For the flexizymes and some synthetases (PylRS), thioester formation is more efficient than ester formation. Conversely, for other synthetases (MetRS and PheRS), acylation of the 3′-thio-tRNA is less efficient than that of canonical tRNAs. Given the well-studied hydrolytic stability of thioesters in water,^33,34^ we were not concerned about 3′-thio-tRNA stability in aqueous buffer and did not explicitly evaluate lability trends for the acylated tRNAs described herein.

### Evidence for Thioesters: Native Chemical Ligation

**Figure 4:**
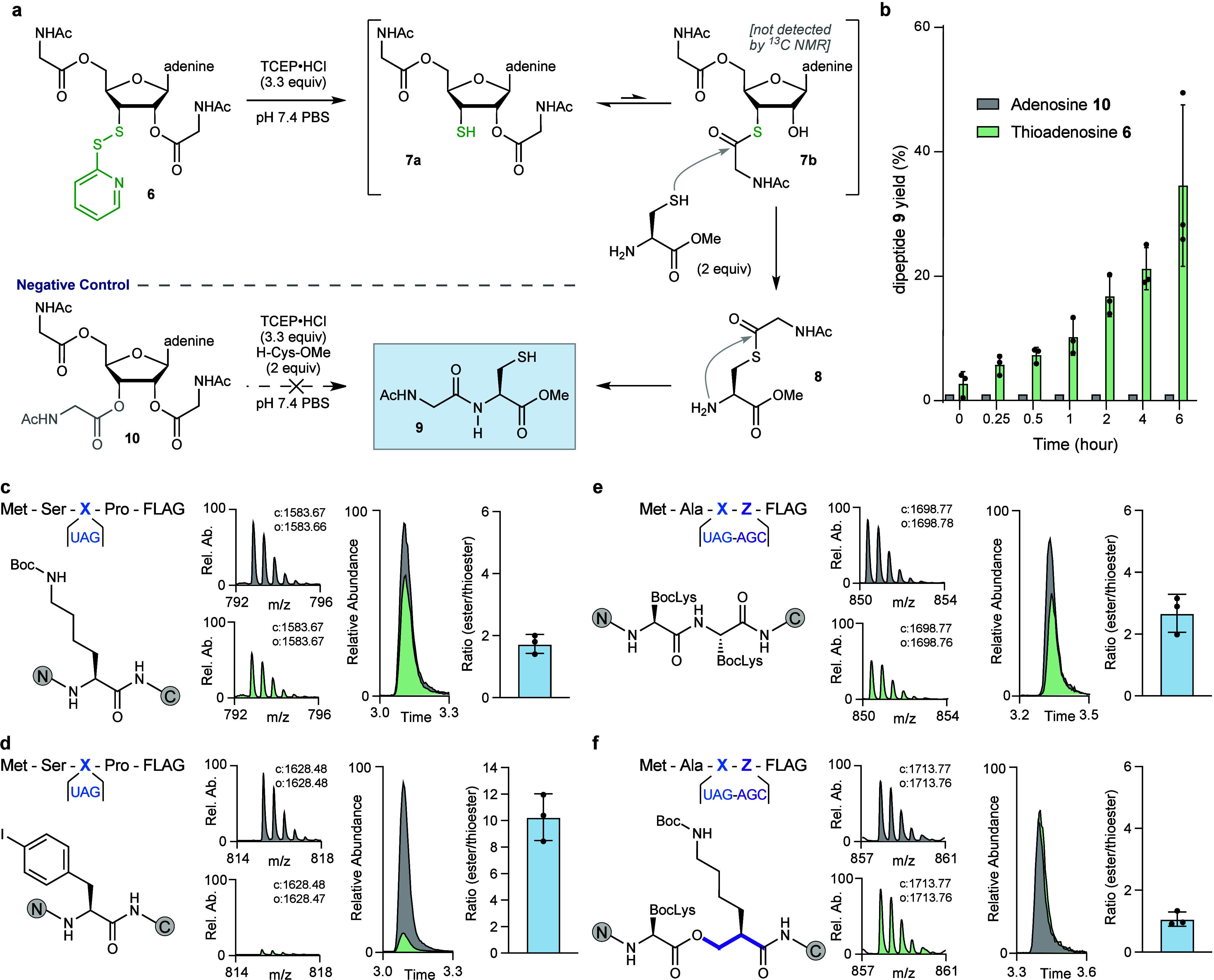
**Native chemical ligation and*****in vitro*****transcription/translation show use of 3′-thioesters in peptide bond formation. a**, Reduction of bisacylated disulfide **6** generates **7a** which has the potential to equilibrate with thioester **7b**, but only **7a** was detected by ^13^C NMR (Figure S4). To establish whether **7b** formed under these conditions, we attempted to trap it upon reaction with H-Cys-OMe to generate dipeptide **9**. Dipeptide **9** is not formed upon reaction of triacyl adenosine **10** with H-Cys-OMe. **b**, Time course illustrating formation of dipeptide **9** under conditions shown in **a**, as measured by UHPLC/HR-MS from protected thio-adenosine **6** or triacyl adenosine **10** after reduction. **c–f**, The results of in vitro translation (IVT) reactions using acylated 3′-oxo- or 3′-thio tRNAs as P-site electrophiles. Shown are the translated peptides, the structure of the incorporated monomer, and *m*/*z* spectra corresponding of the +2 ion of the calculated (c) and observed (o) masses in terms of relative abundance (Rel. Ab.). Extracted ion chromatograms (EIC) of the anticipated products are shown and peak integration values were used to generate the ratio of ester/thioester plot. Error bars are representative of three independent experiments. **c**, Incorporation of BocLys over a recoded stop codon into a short peptide containing a FLAG-tag, synthesized during IVTT. Structure of the portion of the peptide containing BocLys and *m*/*z* spectra corresponding to the +2 ion of peptides with calculated (c) and observed (o) masses in terms of relative abundance (Rel. Ab.) and error (e) reported in ppm (e: 6.3 ppm). Incorporation from an ester is shown in gray and from a thioester in green. Extracted ion chromatograms (EIC) are shown for each mass and peak integration values were used to generate the ratio of ester/thioester plot. Error bars are representative of three independent experiments. **d**, Incorporation of PheI originating from either an ester or a thioester linkage into a short peptide (e: 6.1 ppm). **e**, Incorporation of BocLys from either an ester or a thioester linkage followed by incorporation of BocLys from an ester linkage into a peptide using a slightly different peptide template where the first BocLys is incorporated by stop codon recoding and the second BocLys is incorporated by serine codon recoding (e: 5.9 ppm). **f**, Incorporation of BocLys incorporated by stop codon recoding from either an ester or a thioester linkage followed by incorporation of (*R*)-β^2^-OH by serine codon recoding from an ester linkage into a peptide (e: 5.8 ppm).

Although the above reactions demonstrate 3′-thio-tRNA acylation can occur using several approaches, the resulting product acyl-tRNAs may adopt an unreactive form for use by the ribosome. Beyond ribose conformation, there is the question of exchange of the acyl group between 2′ and 3′-positions. Although prior studies have shown that a complex hydrogen bond network favors 3′-acylated species in the ribosomal PTC,^35^ oxo-esters on tRNAs rapidly exchange between the 2′- and 3′-oxygens, and model studies suggest that the acylated 3′-thio-tRNAs might thermodynamically favor the 2′-oxo-ester rather than the 3′-thioester.^1^ We therefore probed whether the acyl-tRNA would be reactive for transacylation from the 3′-thioester rather than the 2′-oxo-ester. To evaluate the isomerization between 3′-thioester and 2′-oxo-ester, we synthesized bis-acylated adenosine **6** from **5** (Figure 4a) and reduced the disulfide protecting group with TCEP to allow the initially formed **7a** to equilibrate with isomer **7b**. ^13^C NMR analysis of this reaction mixture provided no evidence for thioester **7b** even after a 17 h incubation at 37 °C (see Figures S2–S5). Taking inspiration from studies of native chemical ligation,^36^ we exposed the putative **7a,b** mixture to an excess of cysteine methyl ester.^37^ We observed formation of peptide dimer **9** over time, as supported by HR-MS time studies (Figure 4b; see Extended Data for more information). Given that triacyladenosine **10** did not provide any measurable dimer **9**, we hypothesized that the equilibrium between esters **7a,b**, while strongly favoring ester **7a**, is kinetically accessible, thereby operating under Curtin–Hammett control.^38^ While ^13^C NMR experiments only showed the presence of 2′-oxo-ester, formation of dipeptide **9** provides strong evidence of rapid S–S acyl exchange to transiently form thioester **8**, which immediately reacted via native chemical ligation to liberate dipeptide **9**. These results demonstrate that dynamic interconversion of the two species **7a** and **7b** is kinetically achievable, despite the clear difference in thermodynamic stability.

### *In Vitro* Translation Using Thioester-tRNAs to Generate Peptides

Having demonstrated that a 3′-thioester is kinetically accessible within a nucleoside model system (Figure 4a,b), we asked whether tRNAs containing thioester amino acid linkages would support translation within the ribosomal PTC. We used a DNA template encoding a FLAG-tagged peptide product with a stop codon at the third position in our *in vitro* translation reactions, and detected the resulting peptide using high-resolution LC-MS (Figure 4c,d) as described previously (see Methods). We chose to use our above acylated and described tRNA^Pyl^-S-BocLys or tRNA^Pyl^-O-BocLys to incorporate the unnatural amino acid. Using a PURE system lacking release factor 1 and supplemented with equivalent amounts of monoacylated tRNA^Pyl^-S-BocLys or tRNA^Pyl^-O-BocLys prepared using PylRS (Figure 4c). We quantified the relative amount of peptide produced from analysis of the extracted ion chromatograms (EIC) revealing a 2-fold higher yield when the reaction was supplemented with tRNA^Pyl^-O-BocLys than with tRNA^Pyl^-S-BocLys (Figure 4c, S24). To eliminate potential complications due to the presence of diacylated tRNA promoted by PylRS, we also performed analogous IVT reactions supplemented with tRNA charged with a phenylalanine analog (PheI), using a flexizyme that produces only monoacylated products. We chose to use PheI because phenylalanine analogs have been successfully incorporated into proteins by translational machinery, and generating PheI was more synthetically accessible than an activated Boc-Lys analog. We used the flexizyme eFx to synthesize tRNA^Pyl^-O-PheI and tRNA^Pyl^-S-PheI (Figure S20, S21) and used these charged tRNAs in the IVTT reactions described above. In this case, analysis of the paired EIC traces revealed a 10-fold higher yield when the reaction was supplemented with tRNA^pyl^-O-PheI than with tRNA^pyl^-S-PheI (Figure 4d, S25).

Having shown that ribosomal translation tolerates a single O to S atom substitution when the P-site tRNA is acylated with a noncanonical α-amino acid, we next examined the effect of the atom substitution on translation when incorporating a β^2^-hydroxy acid monomer in the A-site.^31^ This effort required a DNA template in which two adjacent codons are recoded (Figure 4e). We designed a DNA template containing a STOP codon at position 3 and a SER codon at position 4, followed by a FLAG-tag to enable purification and LC-MS analysis. The STOP codon at position 3 was decoded using either tRNA^pyl^-O-BocLys or tRNA^Pyl^-S-BocLys, prepared using PylRS, whereas the SER codon was decoded using tRNA^Pyl(Ser)^-O-BocLys (Figure S22) or tRNA^Pyl(Ser)^-O-(*R*)-β^2^-OH-*N*_ε_-BocLys ((*R*)-β^2^-OH) (Figure 4e,f, S23). The resulting peptide sequence would be Met-Ala-X-Z-FLAG, where X corresponds to BocLys originating from tRNA^Pyl^-O-BocLys or tRNA^Pyl^-S-BocLys and Z corresponds to BocLys or (*R*)-β^2^-OH originating from tRNA^Pyl(Ser)^-O-BocLys or tRNA^Pyl(Ser)^-O-(*R*)-β^2^-OH. In this case, analysis of the EIC traces revealed a 2.7-fold higher yield of a peptide with BocLys at two adjacent positions when the P-site tRNA carried an oxo-ester (Figure 4e, S26). When BocLys was followed by (*R*)-β^2^-OH we observed equivalent yield of peptide when the P-site tRNA had an oxo-ester or thioester, although the overall yield compared to incorporation of two successive BocLys monomers is significantly reduced (Figure 4f, S27). Thus, *in vitro* ribosomal translation tolerates a single O to S atom substitution of the electrophile that participates in bond formation within the PTC.

## Discussion

All extant ribosomes employ oxo-esters to support bond forming reactions, despite the observation that thioesters are generally more reactive toward amine nucleophiles. To explore the effects of thioesters on acyl-tRNA biogenesis and utilization, we synthesized 3′-thio-3′-deoxyadenosine triphosphate and demonstrated that it supports three essential steps of the translation cycle: formation of 3′-thio-tRNA using a CCA-adding enzyme, aminoacylation of 3′-thio-tRNA using both enzymes and ribozymes, and finally ribosome-promoted peptide bond formation. In each and every one of these transformations, the substitution of O for S recapitulated the expected chemistry. In the case of peptide bond formation by the ribosome, the yields of peptides containing both canonical (α-amino acids) and noncanonical (β^2^-hydroxy acid) monomers were comparable regardless of whether the P-site tRNA carried an oxo-ester or a thioester. It is remarkable that, despite differences in bond length, hydrogen bonding potential, and electrophilicity,^39^ we observe efficient translation using thioester-tRNA substrates. Our results indicate that the tRNA and thioester-linked growing polymer chain remain sufficiently well positioned to facilitate bond formation by the extant ribosomal PTC.

Because of the critical role the terminal ribose 3′-position in tRNA plays in protein synthesis, the single atom substitution to sulfur could impact multiple steps in translation. Clues that the 3′-thio-3′-deoxyadenosine triphosphate would be tolerated by a nucleotidyl transferase derive from the fact that ATP analogs with ribose 3′- and 2′- substitutions are often tolerated. Additionally, CCA-adding enzymes are utilized extensively to generate 3′-amino-3′-deoxy-tRNAs for structural biology.^24,40−42^ However, although many ATP analogs can be utilized by the *E. coli* CCA-adding enzyme, they are incorporated less efficiently.^43^ Indeed, we found that any trace ATP contamination present in CCA-adding reactions to introduce 3′-thio-ATP resulted in almost no desired product, indicating the ribose 3′-position likely plays a role in substrate recognition or accommodation.

For the next step in translation, aminoacyl tRNA synthetases have evolved two distinct mechanisms to activate α-amino acid substrates and position them for attack by a tRNA substrate.^44^ Class 1 synthetases bind tRNA substrates on the minor groove side of the acceptor stem and CCA-end and acylate tRNA on the ribose 2′-hydroxyl,^45,46^ whereas class 2 synthetases bind tRNA from the major groove side and acylate tRNA on the ribose 3′-hydroxyl.^47^ We tested both classes of synthetase here, and found that both can tolerate a 3′-thiol in the tRNA substrate. Interestingly, pylRS falls in the class 2 category and is thought to initially charge tRNA substrates using the 3′-hydroxyl, but is also capable of diacylating tRNAs, including 3′-thio-tRNAs.^30,48,49^ Although tRNA aminoacylation by synthetases is an ancient reaction, presumably tRNAs were originally aminoacylated by RNA enzymes, or ribozymes. A modern set of ribozymes capable of this reaction—flexizymes—likely promote acylation through substrate proximity effects.^50,51^ We found that 3′-thio-tRNAs are generally excellent substrates for flexizymes. Given that the aFx flexizyme substrate is a thioester, we tested whether an enzyme is needed at all for charging 3′-thio-tRNAs. Remarkably, we found that ABT-Leu, an amino derivatized benzyl-thioester, could readily serve as an acyl donor to charge 3′-thio-tRNA^Pyl^, opening up a new avenue for charging tRNAs in the future with nonproteinogenic monomers without the need to evolve new aminoacyl-tRNA synthetases or to use flexizymes. Furthermore, thioester exchange may have been a nonspecific mechanism to acylate 3′-thio-nucleotides or μ-helix RNAs for use by early ribozyme mediated translation systems prior to the emergence of catalyzed acylation.

Although the replacement of an ester with a thioester has the potential to increase the rate of peptide bond formation due to increased reactivity toward some nucleophiles, during canonical translation, peptide bond formation is not the rate limiting step.^52^ Furthermore, changes in bond length and hydrogen bonding when the 3′-oxo-ester is replaced with a thioester have the potential to impact the positioning of monomers in the PTC and the water network that supports catalysis.^35^ Because we observe appreciable peptide bond formation with 3′-thio-tRNA substrates, it is clear that this change is not disruptive enough to inhibit translation, giving us insight into possible plasticity in the PTC. Notably during translation, the amino acid must be in the 3′-position to support peptide bond formation.^52,53^ However, we demonstrated that, at equilibrium, acylated 3′-thio-tRNAs strongly favor the 2′-ester over the 3′-thioester. The fact that we observe robust translation suggests binding of acylated 3′-thio-tRNAs in the PTC likely promotes an appreciable shift in the equilibrium to form the 3′-thioester for catalysis. We also hypothesize that EF-Tu, which binds to and delivers 3′-acylated tRNAs to the ribosome during mRNA decoding, likely binds the 3′-thioester rather than the 2′-ester form of the acyl-tRNA to deliver it to the ribosome, thus effectively shifting the thioester/ester equilibrium during A-site tRNA accommodation.^54,55^ Thus, EF-Tu may also be a key component of thioester function in the extant translation system. The critical role of EF-Tu is highlighted in translation reactions incorporating the (*R*)-β^2^-OH monomer. We see less peptide produced in all conditions when using the (*R*)-β^2^-OH monomer, but we did not see a relative decrease in yield when replacing canonical tRNA with 3′-thioester-tRNA. It is known that EF-Tu cannot effectively bind to β^2^-amino acids,^56^ thus requiring EF-Tu independent delivery of β^2^-monomer charged tRNAs to the ribosome, causing tRNA accommodation to become the yield limiting step.

We were drawn to replace the ester in aminoacyl-tRNA with a thioester because they can participate in similar chemistries, and the sulfur substitution would increase the electrophilicity of the substrate in the ribosomal P site. From an origins of life perspective, the use of thioesters deriving from the presence of iron–sulfur clusters, carbon dioxide, and water may have been a critical step in developing an early metabolism consisting of fatty acids and short peptides formed through thioester mediated polymerization.^9−12,17,57−60^ Prebiotic routes to nucleoside analogs with a thiol at the 2′-position have been reported and routes to the 3′-thiol have been proposed.^13,15,61−63^ As the RNA world arose, it is possible that early peptidyl transferase ribozymes could have used thioester-activated amino acids as early substrates with better electrophilicity, marking a key transition to the emergence of the modern systems we see today. However, sulfur differs from oxygen in other ways that could impact biosynthetic reactions, including forming longer bonds and having altered hydrogen bonding capabilities. We have shown that despite the key properties differentiating esters from thioesters, 3′-thio-tRNAs are usable by the full suite of translation machinery to incorporate unnatural monomers into peptides comparably to extant translation systems. In the field of synthetic biology, modifications to the translation machinery, including the ribosome, to incorporate monomers with a range of chemical properties continues to be a key challenge in expanding the diversity of sequence defined polymers.^64,65^ Our orthogonal approach of altering the chemistry of translation itself through replacement of the ester bond linking tRNA to amino acid with a thioester to increase substrate electrophilicity opens a new avenue to explore the incorporation or even allow for reactivity of less nucleophilic monomers in protein biosynthesis.

## Data Availability

All the original data for the manuscript has been deposited at the following location: 10.5281/zenodo.14427666.

## Supporting Information Available

General experimental information, compound synthesis and characterization including ^1^H and ^13^C NMR, 2D NMR, and HRMS data, materials and methods for biochemical experiments, full LC-MS data and replicates for both tRNA and peptide producing experiments, and a table of DNA sequences used to generate both tRNA and mRNA templates (PDF)
Transparent Peer Review report available (PDF)
