# Supplementary material for: Thioesters Support Efficient Protein Biosynthesis by the Ribosome
Source: ACS Cent Sci. 2025 Jan 30;11(3):404–12. doi: 10.1021/acscentsci.4c01698 (PMC11950863; doi:10.1021/acscentsci.4c01698)
Supplement: Supplementary file 1 — oc4c01698_si_001.pdf [file oc4c01698_si_001.pdf]

Supporting Information

Thioesters support efficient protein biosynthesis by the ribosome

Alexandra D. Kent[1†], Jacob G. Robins[2†], Isaac Knudson[1], Jessica T. Vance[3], Alexander C. Solivan[1], Noah X. Hamlish[3], Katelyn A. Fitzgerald[3], Alanna Schepartz[1,3,4,5], Scott J. Miller[2], Jamie H. D. Cate[1,3,4,6]

[1]*Department of Chemistry, University of California, Berkeley, California 94720, United States*
[2]*Department of Chemistry, Yale University, New Haven, Connecticut 06520, United States*
[3]*Department of Molecular and Cell Biology, University of California, Berkeley, California 94720, United States*
[4]*Molecular Biophysics and Integrated Bioimaging Division, Lawrence Berkeley National Laboratory, Berkeley, California 94270, United States*
[5]*Chan Zuckerberg Biohub, San Francisco, California 94158, United States*
[6]*Innovative Genomics Institute, University of California, Berkeley, California 94720, United States*

[†] These authors contributed equally to this work

Email: schepartz@berkeley.edu
Email: scott.miller@yale.edu
Email: j-h-doudna-cate@berkeley.edu

Note: *Table of Contents entries are hyperlinked for simplified access*

Table of Contents

General Information 2
Synthesis of 3′-thio-3′-deoxyadenosine triphosphate (Fig. 1d) 3
((2R,3R,4S,5S)-3-(Benzoylthio)-4,5-bis(formyloxy)tetrahydrofuran-2-yl)methyl 4-methylbenzoate (S1a) & ((2R,3R,4S,5S)-5-acetoxy-3-(benzoylthio)-4-(formyloxy)tetrahydrofuran-2-yl)methyl 4-methylbenzoate (S1b) 3
((2R,3R,4S,5R)-4-Acetoxy-5-(6-benzamido-9H-purin-9-yl)-3-(benzoylthio)tetrahydrofuran-2-yl)methyl 4-methylbenzoate (S2a) & ((2R,3R,4S,5R)-5-(6-benzamido-9H-purin-9-yl)-3-(benzoylthio)-4-(formyloxy)tetrahydrofuran-2-yl)methyl 4-methylbenzoate (S2b) 9
(2R,3S,4S,5R)-2-(6-Amino-9H-purin-9-yl)-5-(hydroxymethyl)-4-(pyridin-2-yldisulfaneyl)tetrahydrofuran-3-ol (4) 13
Figure S1: Variable Temperature 1H NMR Studies for Compound 4 16
Tetrakis(triethylammonium) ((2R,3S,4S,5R)-5-(6-amino-9H-purin-9-yl)-4-hydroxy-3-(pyridin-2-yldisulfaneyl)tetrahydrofuran-2-yl)methyl triphosphate (5) 17
Evidence for thioester intermediates: native chemical ligation studies 18
((2R,3R,4S,5R)-4-((Acetylglycyl)oxy)-5-(6-amino-9H-purin-9-yl)-3-(pyridin-2-yldisulfaneyl)tetrahydrofuran-2-yl)methyl acetylglycinate (6) 18
(2R,3R,4R,5R)-2-(((Acetylglycyl)oxy)methyl)-5-(6-amino-9H-purin-9-yl)tetrahydrofuran-3,4-diyl bis(2-acetamidoacetate) (10) 24
Dimethyl 3,3'-disulfanediyl(2R,2'R)-bis(2-(2-acetamidoacetamido)propanoate) (S3): 27
NMR studies on disulfide 6 31
Figure S2: 1H NMR (D2O, 400 MHz, standard view) at t0, prior to heating 32
Figure S3: 1H NMR (D2O, 600 MHz, standard view) after 17 h at 37 °C 33
Figure S4: 13C NMR (D2O, 600 MHz, standard view) after 17 h at 37 °C 34
Figure S5: 1H/13C HMBC NMR Spectrum after 17 h at 37 °C (600/151 MHz, D2O) 35
Methods for and analysis of mass spectrometry data for NCL experiment: 36
Synthesis of para-iodophenylalanine cyanomethyl ester 37
Cyanomethyl (S)-2-((tert-butoxycarbonyl)amino)-3-(4-iodophenyl)propanoate (S5) 37
Cyanomethyl (S)-2-amino-3-(4-iodophenyl)propanoate hydrochloride (S6) 40
Biochemistry Methods 43

Supplementary Figures 47
Figure S6. Intact tRNA LC-MS of truncated-tRNAs (-A). 47
Figure S7. Intact tRNA LC-MS of full length tRNAs. 48
Figure S8. Intact tRNA LC-MS of 3′-thio-tRNAs. 49
Figure S9. Intact tRNA LC-MS of Nε-boc-L-lysine (BocLys) added to 3′-thio-tRNAPyl using PylRS. 50
Figure S10. Intact tRNA LC-MS of Nε-boc-L-lysine (BocLys) added to tRNAPyl using PylRS. 51
Figure S11. Intact tRNA LC-MS of homopropargylglycine (Hpg) added to 3′-thio-tRNAfMet using MetRS. 52
Figure S12. Intact tRNA LC-MS of homopropargylglycine (Hpg) added to tRNAfMet using MetRS. 53
Figure S13. Intact tRNA LC-MS of phenylalanine (Phe) added to 3′-thio-tRNAPhe using PheRS. 54
Figure S14. Intact tRNA LC-MS of phenylalanine (Phe) added to tRNAPhe using PheRS. 55
Figure S15. Intact tRNA LC-MS of phenylalanine (Phe) added to 3′-thio-tRNAPyl using eFx. 56
Figure S16. Intact tRNA LC-MS of phenylalanine (Phe) added to tRNAPyl using eFx. 57
Figure S17. Intact tRNA LC-MS of leucine (Leu) added to 3′-thio-tRNAPyl using aFx. 58
Figure S18. Intact tRNA LC-MS of leucine (Leu) added to tRNAPyl using aFx. 59
Figure S19. Intact tRNA LC-MS of leucine (Leu) added to 3′-thio-tRNAPyl using thioester exchange. 60
Figure S20. Intact tRNA LC-MS of iodo-phenylalanine (PheI) added to 3′-thio-tRNAPyl using eFx. 61
Figure S21. Intact tRNA LC-MS of iodo-phenylalanine (PheI) added to tRNAPyl using eFx. 62
Figure S22. Intact tRNA LC-MS of Nε-boc-L-lysine (BocLys) added to tRNAPyl(Ser) using PylRS. 63
Figure S23. Intact tRNA LC-MS of (R)-β2-OH-Nε-BocLys ((R)-β2-OH) added to tRNAPyl(Ser) using PylRS. 64
Figure S24. Incorporation of BocLys into a peptide from either 3′-thio-tRNAPyl or tRNAPyl. 65
Figure S25. Incorporation of PheI into a peptide from either 3′-thio-tRNAPyl or tRNAPyl. 66
Figure S26. Incorporation of two BocLys monomers into a peptide from either 3′-thio-tRNAPyl or tRNAPyl. 67
Figure S27. Incorporation of (R)-β2-OH into a peptide following BocLys incorporated from either 3′-thio-tRNAPyl or tRNAPyl. 68
Supplementary Tables 69
Table 1: DNA sequences. 69
Table 2: Peptide Sequences. 70
References 71

General Information

General Synthetic Methods: Unless otherwise stated, all reactions were carried out under an atmosphere of dry nitrogen in flame-dried glassware with magnetic stirring. Thin layer chromatography (TLC) was performed on EMD Millipore silica gel 60 F254 precoated plates (0.25 mm thickness) or 60 Å Silica Gel F254 pre-coated plates (0.25 mm thickness). Visualization was accomplished using UV light and either $KMnO_4$ solution or cerium ammonium molybdate (CAM) solution followed by heating. Flash chromatography was performed under positive air pressure using Siliaflash-P60 silica gel (40-63 µm) purchased from Silicycle or a Teledyne ISCO Combiflash NextGen 300+ equipped with a 4g RediSep column. Reversed-phase flash chromatography (RP-FCC) was performed using an automated Biotage® Isolera™ One flash purification system equipped with a 12, 30, 60, or 120 g SNAP C18 (HS 50 µm silica) or SNAP Ultra C18 (HP Sphere, 25 µm silica) cartridge. Yield refers to isolated yield of pure material unless otherwise noted.

Instrumentation and Data Acquisition: Proton (^{1}H) and carbon (^{13}C) magnetic resonance spectra were obtained on a Bruker AVIII HD 5 MHz spectrometer equipped with a 500 MHz Bruker AVIII HD with BBO Prodigy cryoprobe, a 400 MHz Bruker AVIII HD with BBFO probe, a 600 MHz Varian INOVA, Agilent 400 MHz and 500 MHz spectrometers, or Bruker 500 Mhz spectrometer equipped with a 1H/BB iProbe, using solvent resonances for internal chemical shift calibration (^{1}H NMR: $CDCl_3$ at 7.26 ppm, C_6D_6 at 7.16 ppm, MeOD at 3.31 ppm, DMSO-d6 at 2.50 ppm, D_2O at 4.79 ppm; ^{13}C NMR: $CDCl_3$ at 77.00 ppm, C_6D_6 at 128.06 ppm, MeOD at 49.00 ppm, DMSO-d6 at 39.52). High-resolution mass spectrometry (HRMS) was conducted by the Chemical and Biophysical Instrumentation Center in the chemistry department at Yale University, on a Waters Xevo Q-TOF high-resolution Mass Spectrometry using ESI, or in the Schepartz lab with an Agilent 6530 Accurate-Mass Q-TOF LC-MS using ESI.

NMR Data Reporting: The following format is used for the presentation of 1H NMR spectroscopic data: magnet strength, analysis solvent, chemical shift (ppm), multiplicity (s = singlet, br s = broad singlet, app s = apparent singlet, d = doublet, bd = broad doublet, t = triplet, app t = apparent triplet, q = quartet, app q = apparent quartet, dd = doublet of doublets, td =

triplet of doublets, app td = apparent triplet of doublets, ddd = doublet of doublet of doublets, ddt = doublet of doublet of triplets, app ddt = apparent doublet of doublet of triplets, dddd = doublet of doublet of doublet of doublets, m = multiplet), *J*-coupling constants (Hz), and integration.

Materials: Unless otherwise stated, technical grade solvents and reagents were used as received. Anhydrous acetonitrile (MeCN), tetrahydrofuran (THF), diethyl ether (Et_2O), methylene chloride (CH_2Cl_2), toluene (PhMe), and dimethylformamide (DMF) were dried over alumina and dispensed under argon from a Glass Contour Seca Solvent Purification System. Solvent ratios are reported as volume ratios. All other chemicals were purchased and used as received. No unexpected or unusually high safety hazards were encountered.

Synthesis of 3'-thio-3'-deoxyadenosine triphosphate (Fig. 1d)

((2*R*,3*R*,4*S*,5*S*)-3-(Benzoylthio)-4,5-bis(formyloxy)tetrahydrofuran-2-yl)methyl 4-methylbenzoate (S1a) & ((2*R*,3*R*,4*S*,5*S*)-5-acetoxy-3-(benzoylthio)-4-(formyloxy)tetrahydrofuran-2-yl)methyl 4-methylbenzoate (S1b)

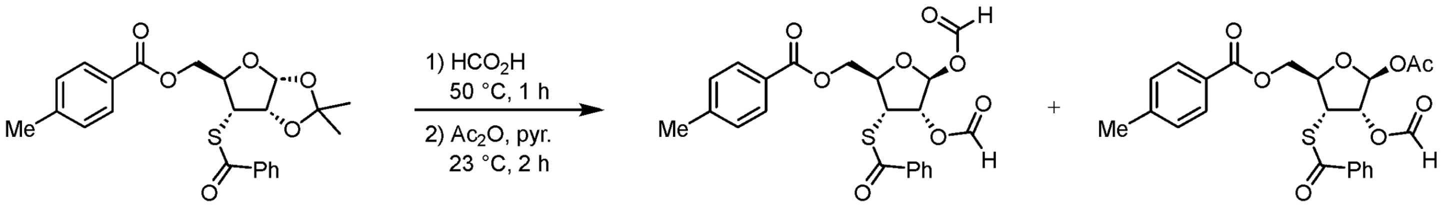

A 250 mL round-bottomed flask was charged with acetonide **2**[1] (3.082 g, 7.199 mmol, 1.0 equiv) and formic acid (48 mL). After stirring at 50 °C for 1 h, the reaction mixture was concentrated *in vacuo* and dried azeotropically with 1-butanol (1 x 20 mL) and toluene (2 x 20 mL). The crude brown oil was dried on hi-vac overnight.

The crude material was treated with pyridine (48 mL) and Ac_2O (35 mL) and stirred at room temperature. After 2 h, the reaction mixture was poured onto crushed ice and the aqueous layer was extracted with Et_2O (3 x 30 mL). The combined ethereal extracts were washed with brine, dried over sodium sulfate, filtered, and concentrated *in vacuo*. The crude material was purified by flash column chromatography (SNAP Ultra 100 g, CV = 132 mL, flow rate = 100 mL•min^{-1}, silica gel; Column 1 = 0% EtOAc/Hex for 1 CV, 0-30% EtOAc/Hex linear gradient over 6 CV, 30% EtOAc/Hex for 2 CV, 30-50% EtOAc/Hex linear gradient over 3 CV, 50-100% EtOAc/Hex linear gradient over 2 CV, 100% EtOAc/Hex for 1 CV; Column 2 = 0% EtOAc/Hex for 1 CV, 0-30% EtOAc/Hex linear gradient over 4 CV, 30% EtOAc/Hex for 4 CV, 30-100% EtOAc/Hex linear gradient over 5 CV, 100% EtOAc/Hex for 1 CV) to provide a mixture title compounds in a 7.2:1 ratio as a pale-yellow foam (2.0992 g). The product distribution ratio was determined via ^{1}H NMR spectroscopic analysis by integration of peaks at δ 6.35 ppm (bis-formate **S1a**) and δ 6.25 ppm (formate-acetate **S1b**). Additionally, small amounts of a bis-acetate (6.31 ppm, 7%, confirmed by HRMS) and an undefined anomer (6.22 ppm, 2%) were present but not included in the tabulated spectroscopic data below.

Physicochemical Properties

pale-yellow foam

^{1}H NMR (600 MHz, $CDCl_3$) δ 8.14 (dd, *J* = 2.5, 1.1 Hz, 0.74H [0.43H + 0.32 H]), 7.98 (t, *J* = 8.2 Hz, 2H), 7.92 (dt, *J* = 7.3, 1.2 Hz, 2H [includes additional 0.43H formate proton]), 7.61 (tt, *J* = 7.3, 1.3 Hz, 1H), 7.49 – 7.44 (m, 2H), 7.21 (dd, *J* = 8.2, 3.2 Hz, 2H), 6.35 (s, 0.43H), 6.25 (s, 0.32H), 5.58 (d, *J* = 4.6 Hz, 0.48H), 5.56 (d, *J* = 4.6 Hz, 0.37H), 4.80 – 4.71 (m, 2H), 4.61 – 54 (m, 1H), 4.37 – 4.33 (m, 1H), 2.40 (s, 3H), 1.98 (d, *J* = 1.0 Hz, 1H [3x 0.32H for acetate protons]).

^{13}C NMR (151 MHz, $CDCl_3$) δ 188.8, 188.6, 169.0, 166.1, 159.1, 159.0, 158.9, 158.6, 144.0, 143.9, 135.8, 135.7, 134.2, 134.2, 129.81, 129.76, 129.1, 129.1, 128.82, 128.81, 127.54, 127.52, 127.0, 126.9, 98.3, 97.9, 82.1, 81.7, 76.5, 76.3, 63.0, 62.9, 42.1, 42.0, 21.7 (two benzylic ^{13}C signals overlap), 20.9.

TLC (EtOAc/Hex = 30/70): R_f = 0.4

HRMS (ESI) for bis-formate **S1a**: Calc. for $[C_{22}H_{20}O_8S+Na]^+$: 467.0771; Found: 467.0737

HRMS (ESI) for formate-acetate **S1b**: Calc. for $[C_{23}H_{22}O_8S+Na]^+$: 481.0928; Found: 481.0900

HRMS (ESI) for bis-acetate: Calc. for $[C_{24}H_{24}O_8S+Na]^+$: 495.1085; Found: 495.1054

Compounds S1a and S1b (^1H, 600 MHz, $CDCl_3$, standard view)

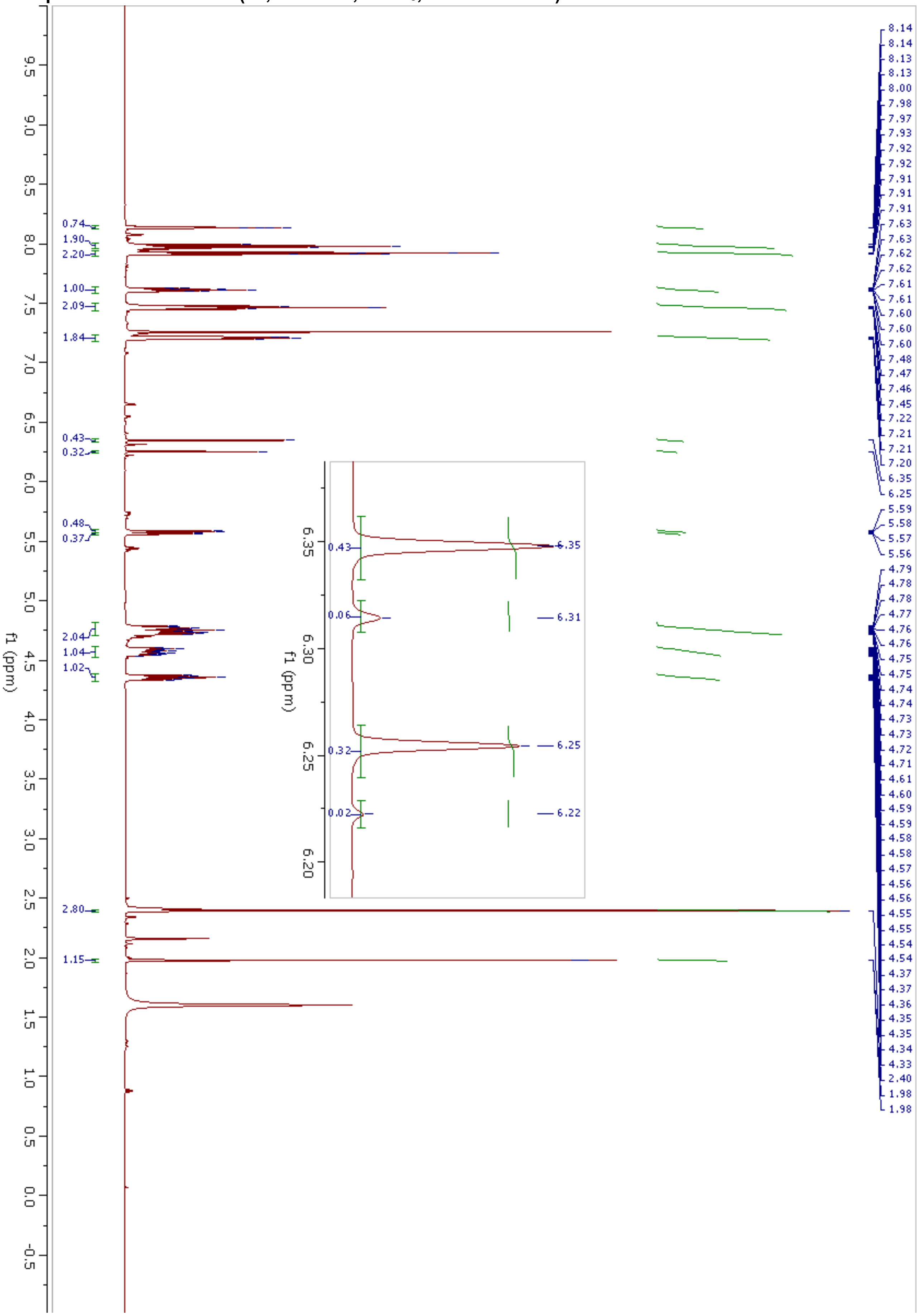

Compounds S1a and S1b ($^{13}C\{^{1}H\}$, 151 MHz, $CDCl_3$, standard view)

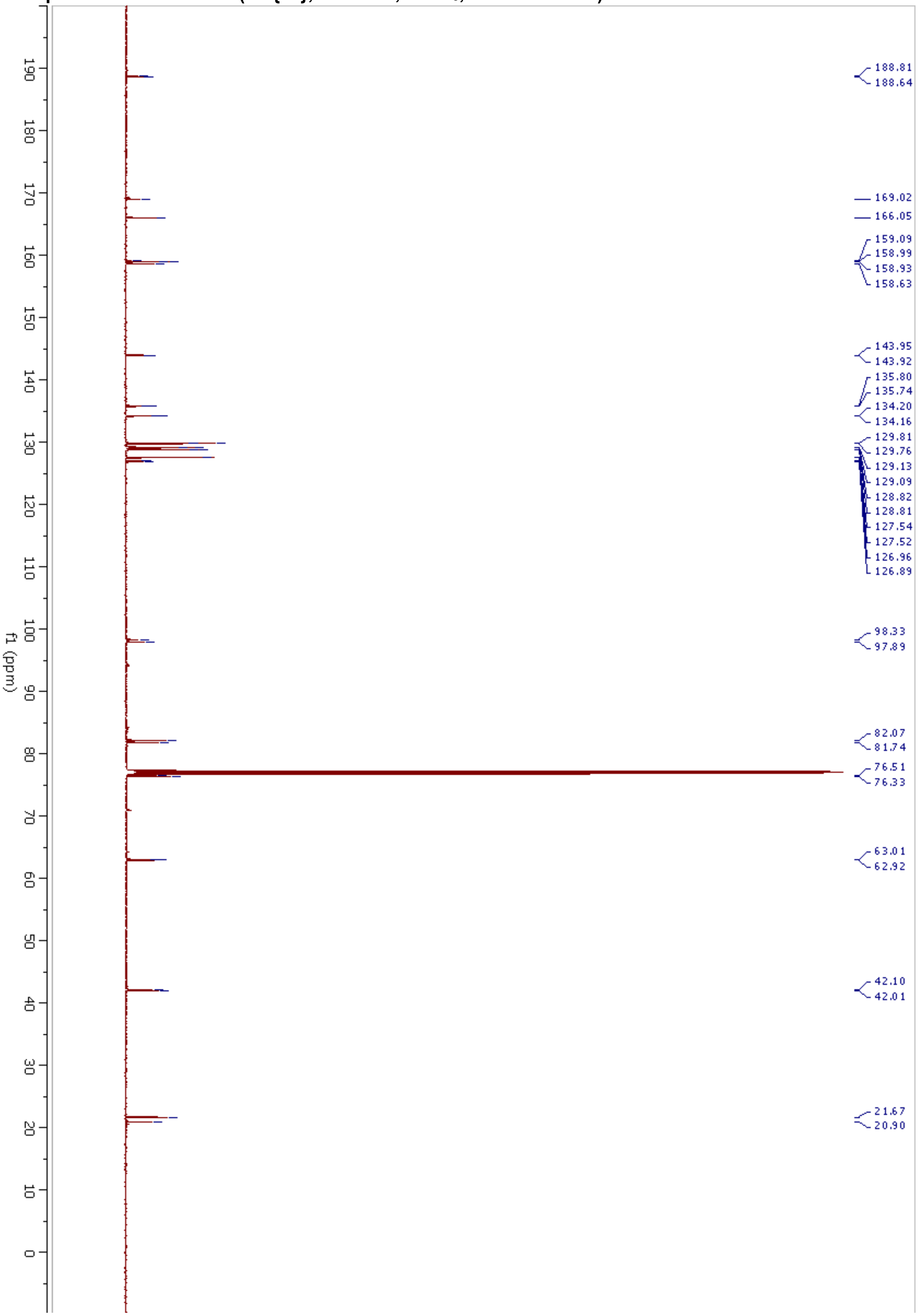

$^{1}H/^{13}C$ HSQC NMR Spectrum of Compounds S1a and S1b (600/151 MHz, $CDCl_3$)

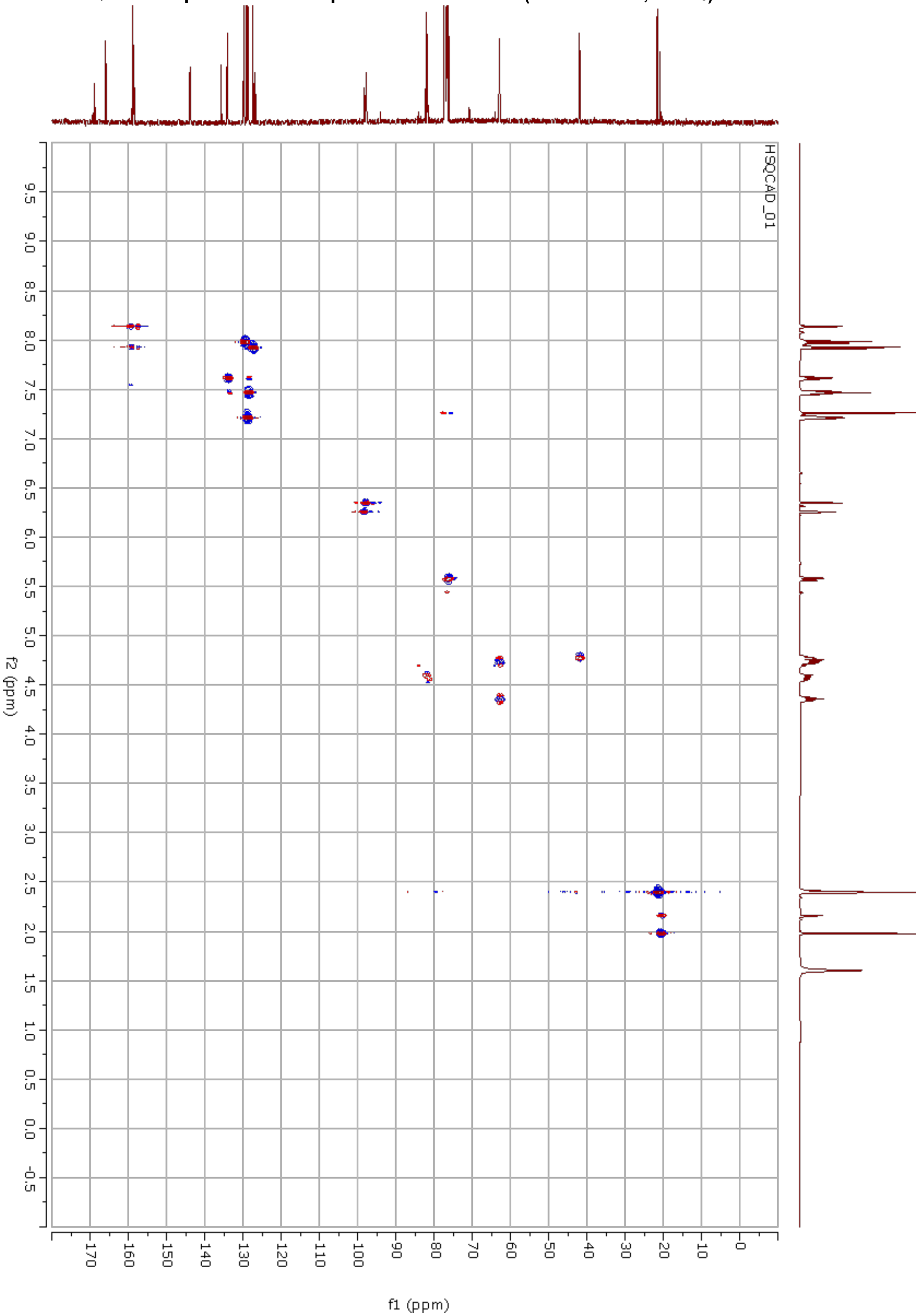

$^{1}H/^{13}C$ HMBC NMR Spectrum of Compounds S1a and S1b (600/151 MHz, $CDCl_3$)

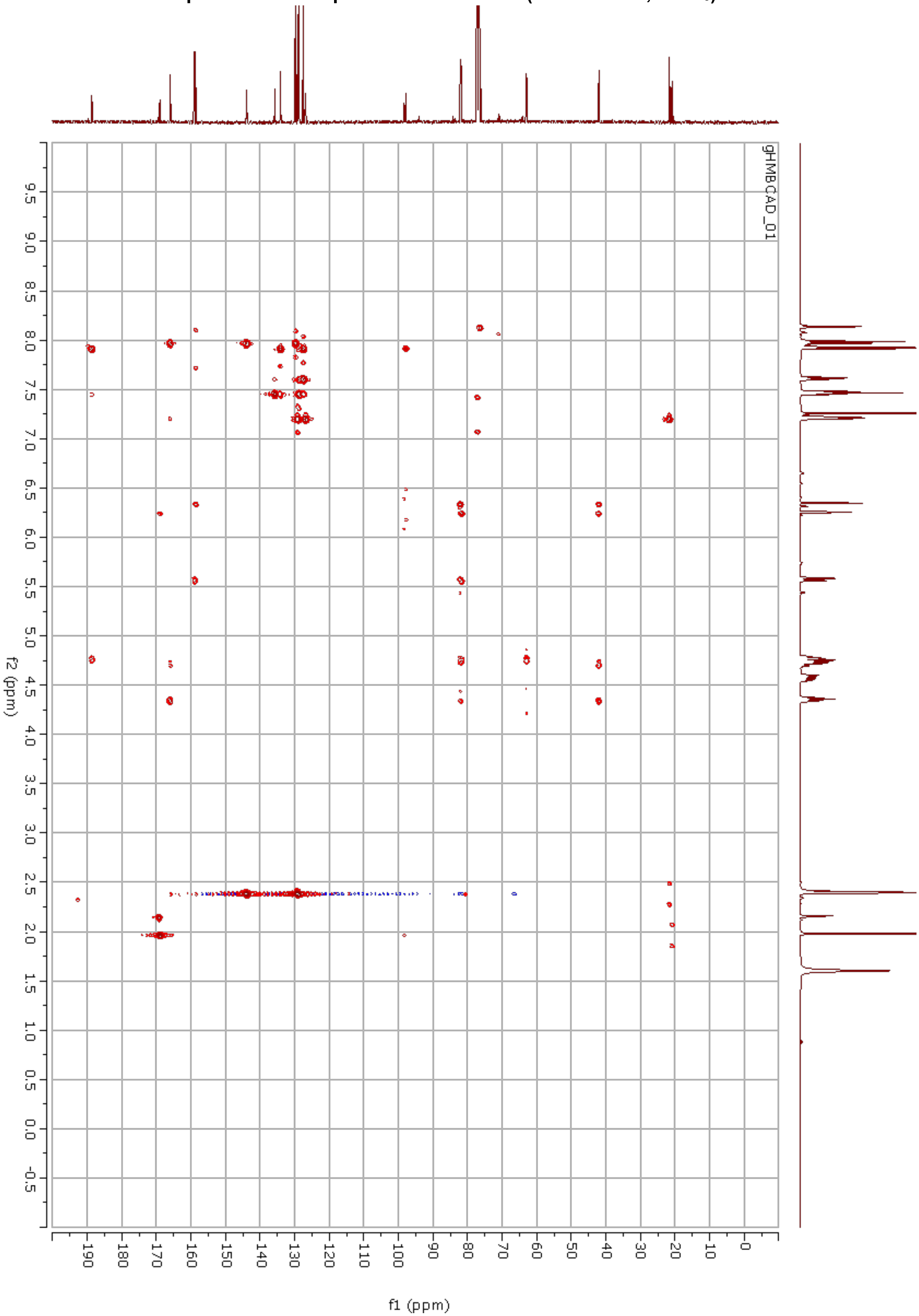

((2*R*,3*R*,4*S*,5*R*)-4-Acetoxy-5-(6-benzamido-9*H*-purin-9-yl)-3-(benzoylthio)tetrahydrofuran-2-yl)methyl 4-methylbenzoate (S2a) & ((2*R*,3*R*,4S,5*R*)-5-(6-benzamido-9*H*-purin-9-yl)-3-(benzoylthio)-4-(formyloxy)tetrahydrofuran-2-yl)methyl 4-methylbenzoate (S2b)

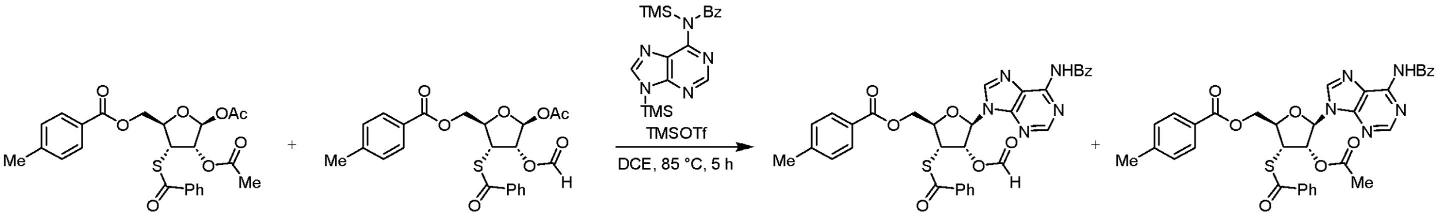

Preparation of *N*-(trimethylsilyl)-*N*-(9-(trimethylsilyl)-9*H*-purin-6-yl)benzamide **3**:[2] A flame-dried 250 mL Schlenk tube equipped with a magnetic stir bar and reflux condenser was cooled under a stream of $Ar_{(g)}$ and charged with N^6-benzoyladenine (1.334 g, 5.578 mmol, 1.2 equiv), bis(trimethylsilyl)acetamide (5.58 mL), HMDS (22.3 mL), and PhMe (28 mL). The resulting cloudy white suspension was submerged in an oil bath at 130 °C and stirred for 16 h to afford a homogenous yellow solution. The reaction vessel was cooled to room temperature and concentrated *in vacuo* by connection to a hi-vac system to afford a dark yellow oil. (NB: desilylation will result from even brief exposure to air or moisture, as evidenced by the formation of a white powder).

The crude persilylated nucleobase **3** was purged with $N_{2(g)}$ and a solution of glycosyl acetates **S1a,b** (2.0992 g, 4.648 mmol, 1.0 equiv) in DCE (23.2 mL, including washes) was added via Schlenk arm, followed by TMSOTf (1.26 mL, 6.972 mmol, 1.5 equiv). The resulting brown solution was stirred at 85 °C for 5 h, cooled to room temperature, and quenched with saturated aqueous $NaHCO_3$. The aqueous layer was extracted with EtOAc (3 x 30 mL) and the combined organic extracts were washed with brine, dried over sodium sulfate, filtered, and concentrated *in vacuo* to provide a brown semi-solid. The crude material was purified by flash column chromatography (SNAP Ultra 100 g, CV = 132 mL, flow rate = 100 mL•min^{-1}, silica gel; Column 1 = 70% EtOAc/Hex for 1 CV, 70-100% EtOAc/Hex linear gradient for 10 CV, 100% EtOAc for 2 CV; Column 2 = 20% EtOAc/Hex for 1 CV, 20-100% EtOAc/Hex linear gradient for 6 CV, 100% EtOAc for 7 CV) to afford the title compounds, as an inconsequential 3.6:1 mixture of acetate and formate esters, as a white foam (634.8 mg, 0.9915 mmol, 21% yield). The product distribution ratio was determined via ^{1}H NMR spectroscopic analysis by integration of peaks at δ 6.20 ppm (formate) and δ 6.18 ppm (acetate).

Physicochemical Properties

Off-white foam

^{1}H NMR (600 MHz, $CDCl_3$) δ 8.91 (s, 1H), 8.85 (s, 1H), 8.21 (s, 1H), 8.17 (d, *J* = 4.0 Hz, 1H), 8.04 – 8.01 (m, 2H), 7.97 – 7.95 (m, 2H), 7.89 – 7.86 (m, 2H), 7.65 – 7.60 (m, 2H), 7.57 – 7.52 (m, 2H), 7.50 – 7.46 (m, 2H), 7.19 (d, *J* = 7.9 Hz, 2H), 6.28 (d, *J* = 5.9 Hz, 0.77H), 6.20 (d, *J* = 1.6 Hz, 0.76H), 6.18 (d, *J* = 1.8 Hz, 0.21H), 6.10 (dd, *J* = 5.9, 1.8 Hz, 0.21H), 5.39 – 5.34 (m, 1H), 4.86 – 4.82 (m, 1H), 4.67 – 4.64 (m, 1H), 4.55 – 4.51 (m, 1H), 2.37 (s, 3H), 2.21 (s, 0.63H).

^{13}C NMR (151 MHz, $CDCl_3$) δ 188.3, 166.2, 164.3, 159.1, 1532, 151.1, 149.7, 144.1, 141.6, 135.8, 134.2, 133.6, 132.9, 129.7, 129.2, 128.9, 128.9, 127.8, 127.6, 126.6, 123.5, 89.4, 82.2, 76.7, 62.5, 42.9, 21.7.

TLC (EtOAc/Hex = 80/20): R_f = 0.3

HRMS (ESI) for formate **S2a**: Calc. for $[C_{33}H_{27}N_5O_7S+H]^+$: 638.1704; Found: 638.1689

HRMS (ESI) for acetate **S2b**: Calc. for $[C_{34}H_{29}N_5O_7S+H]^+$: 652.1860; Found: 652.1898

Compounds S2a and S2b (¹H, 600 MHz, $CDCl_3$, standard view)

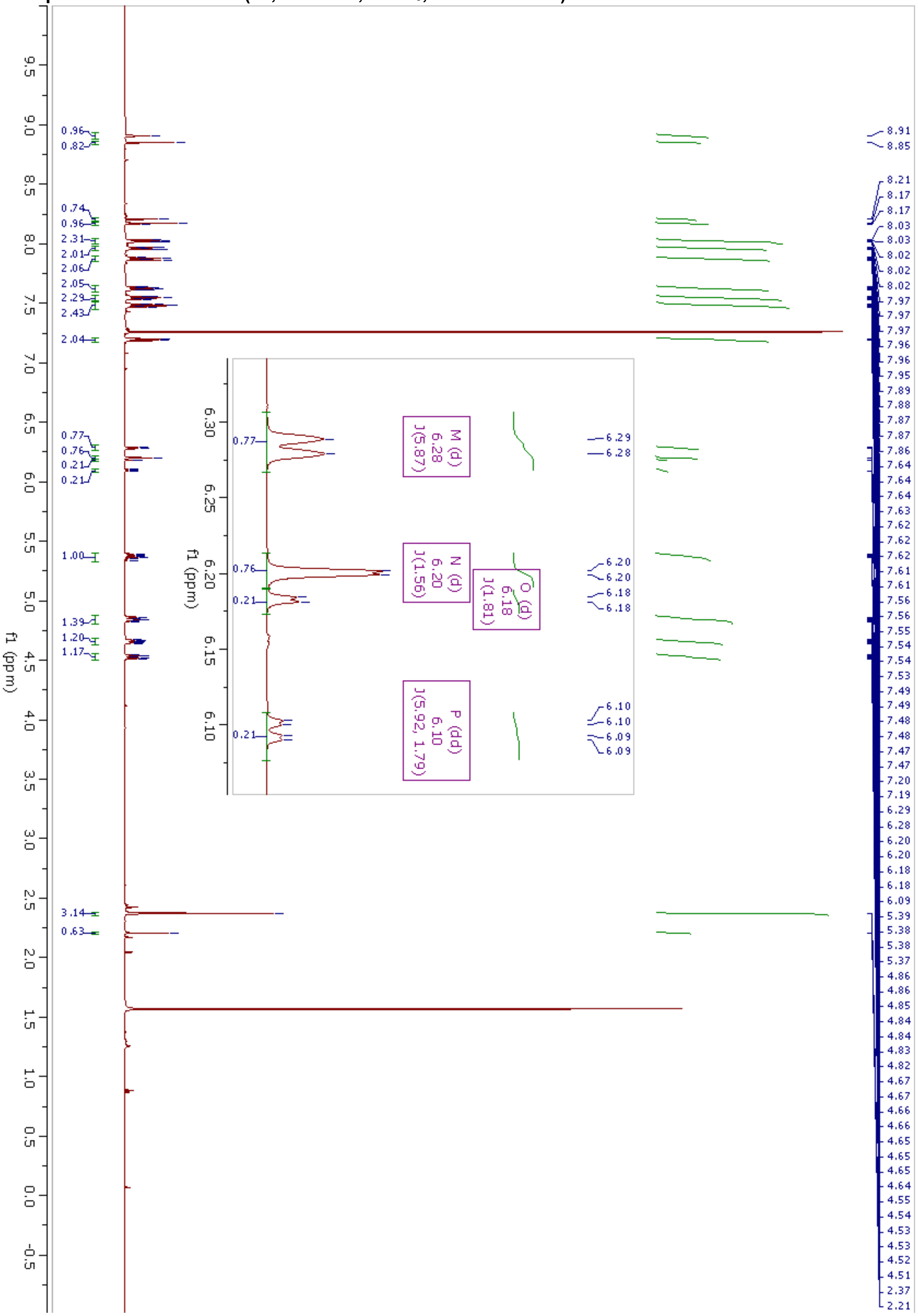

Compounds S2a and S2b (${}^{13}C\{{}^{1}H\}$, 151 MHz, $CDCl_3$, standard view)

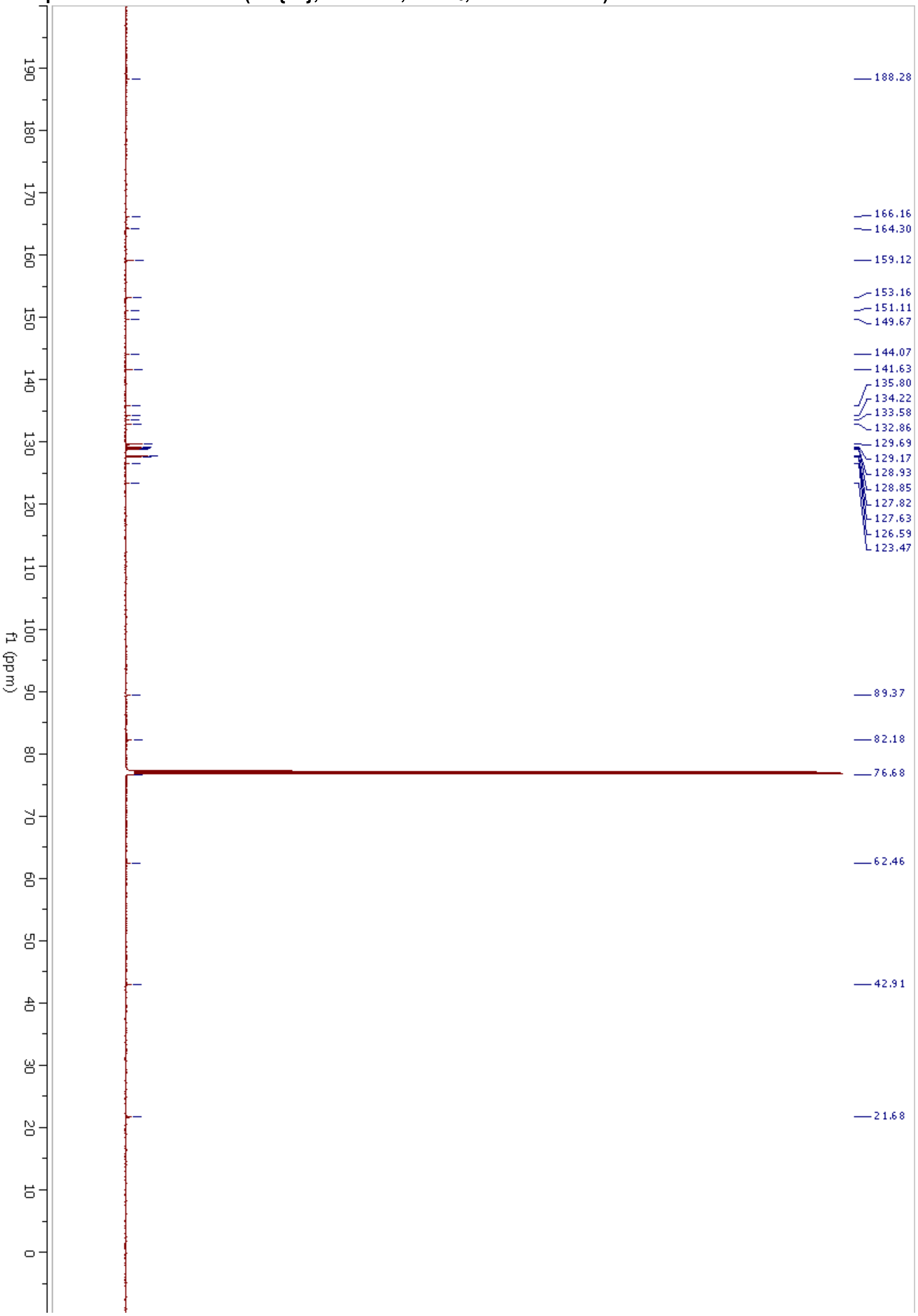

$^1H/^{13}C$ HSQC NMR Spectrum of Compounds S2a and S2b (600/151 MHz, $CDCl_3$)

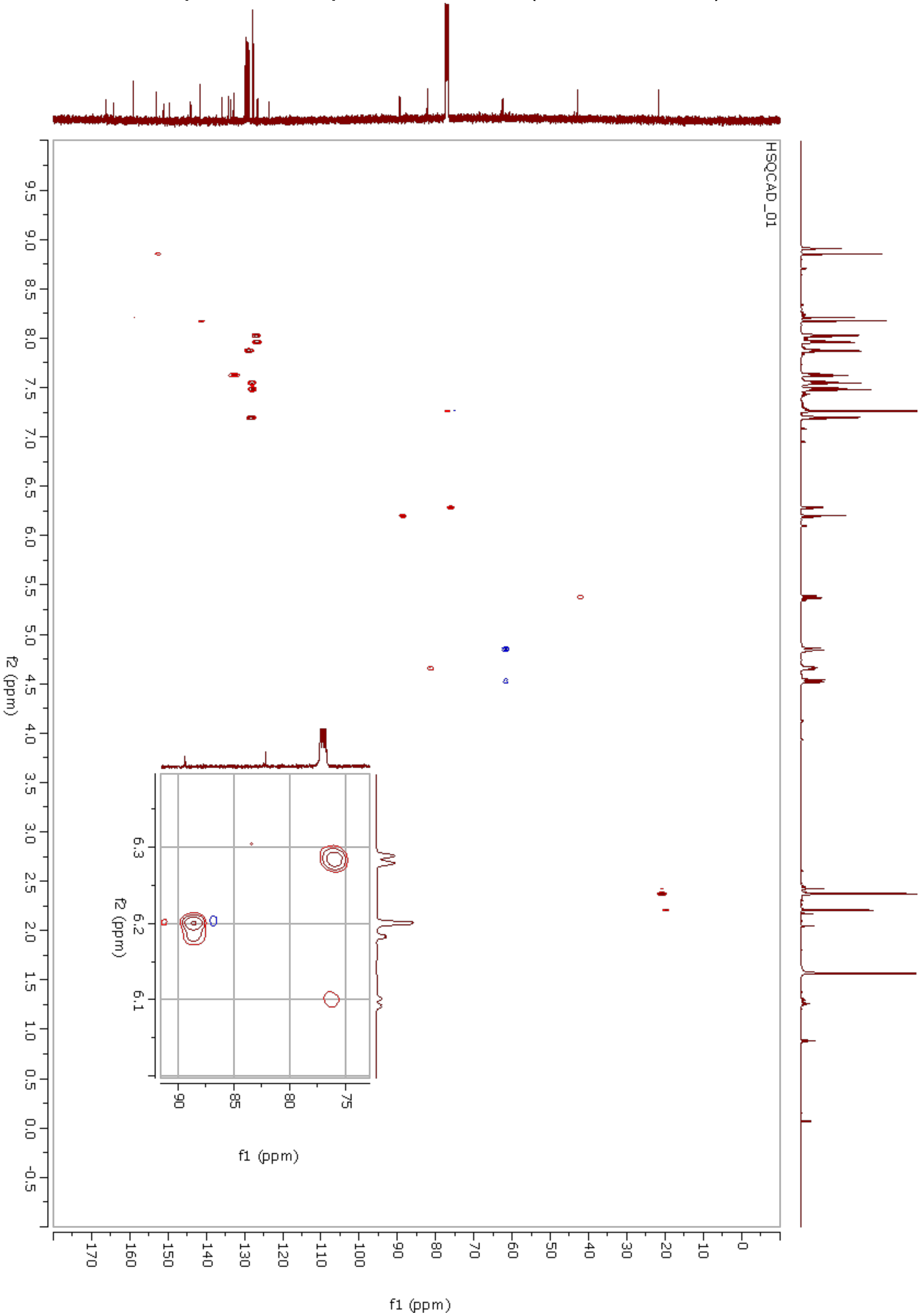

(2*R*,3*S*,4*S*,5*R*)-2-(6-Amino-9*H*-purin-9-yl)-5-(hydroxymethyl)-4-(pyridin-2-yldisulfaneyl)tetrahydrofuran-3-ol (4)

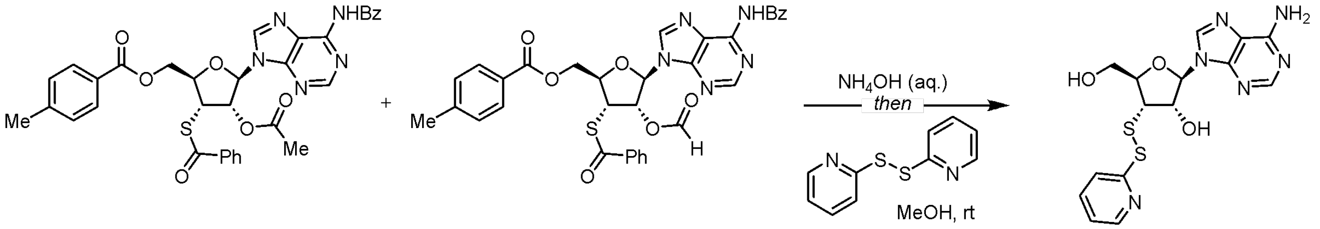

A 250 mL round-bottomed flask was charged with nucleotides **S2a,b** (604.1 mg, 0.941 mmol, 1.0 equiv) and NH_4OH (94 mL). The vessel was sealed with a rubber septum and the mixture was stirred at room temperature. After 1 h, the cloudy suspension had clarified. After 24 h, the reaction mixture was conc. *in vacuo* to ~1/2 volume then sparged with $Ar_{(g)}$ for 10 min. A separately prepared solution of aldrithiol (310.7 mg, 1.412 mmol, 1.5 equiv) in degassed MeOH (35 mL) was added. After stirring at room temperature for 1 h, the mixture was conc. *in vacuo*. The resulting tan solid was azeotropically dried with PhMe (3 x 10 mL) and Et_2O (3 x 10 mL), filtered, and washed with Et_2O to provide the title compound (323.9 mg, 0.826 mmol, 88% yield) as a mixture of rotamers about the S–S bond, as supported by the coalescence of signals at 4.89 ppm in variable temperature ^{1}H NMR experiments (25–130 °C taken at 15 °C intervals).

Physicochemical Properties

tan powder

^{1}H NMR (600 MHz, DMSO-d6) δ 8.49 – 8.48 (m, 0.1H), 8.46 (dt, *J* = 4.9, 1.1 Hz, 0.7H), 8.38 (s, 1H), 8.12 (s, 0.2H), 8.10 (s, 0.7H), 7.88 – 7.79 (m, 2H), 7.32 (s, 2H), 7.26 (ddd, *J* = 6.6, 4.8, 1.8 Hz, 1H), 6.56 (d, *J* = 5.1 Hz, 0.85H), 6.26 (d, *J* = 5.2 Hz, 0.15H), 6.01 (d, *J* = 3.4 Hz, 0.8H), 5.96 (d, *J* = 3.5 Hz, 0.2H), 5.47 (dd, *J* = 6.5, 4.8 Hz, 0.17H), 5.36 (dd, *J* = 6.3, 4.8 Hz, 0.85H), 4.85 (td, *J* = 5.4, 3.5 Hz, 0.85H), 4.81 (q, *J* = 5.0 Hz, 0.15H), 4.31 (dt, *J* = 6.8, 3.2 Hz, 0.85H), 4.28 (dd, *J* = 6.9, 3.4 Hz, 0.15H), 4.09 (q, *J* = 5.3 Hz, 0.2H), 3.95 (dd, *J* = 7.2, 5.7 Hz, 1H), 3.89 – 3.84 (m, 0.2H), 3.80 (ddd, *J* = 12.3, 4.8, 2.7 Hz, 0.9H), 3.66 (ddd, *J* = 11.6, 6.7, 3.8 Hz, 0.2H), 3.55 (ddd, *J* = 12.2, 6.4, 3.8 Hz, 0.9H).

^{13}C NMR (151 MHz, DMSO-d6) δ 158.9, 156.13, 156.11, 152.5, 149.7, 149.6, 148.8, 139.5, 139.4, 138.2, 137.9, 121.8, 121.4, 119.7, 119.5, 119.2, 119.1, 89.3, 84.7, 74.9, 61.4, 53.7.

HRMS (ESI): Calc. for [$C_{23}H_{26}N_7O_8S_2$+H]: 591.1439; Found: 591.1451

Compound 4 (^{1}H, 600 MHz, DMSO-d6, standard view)

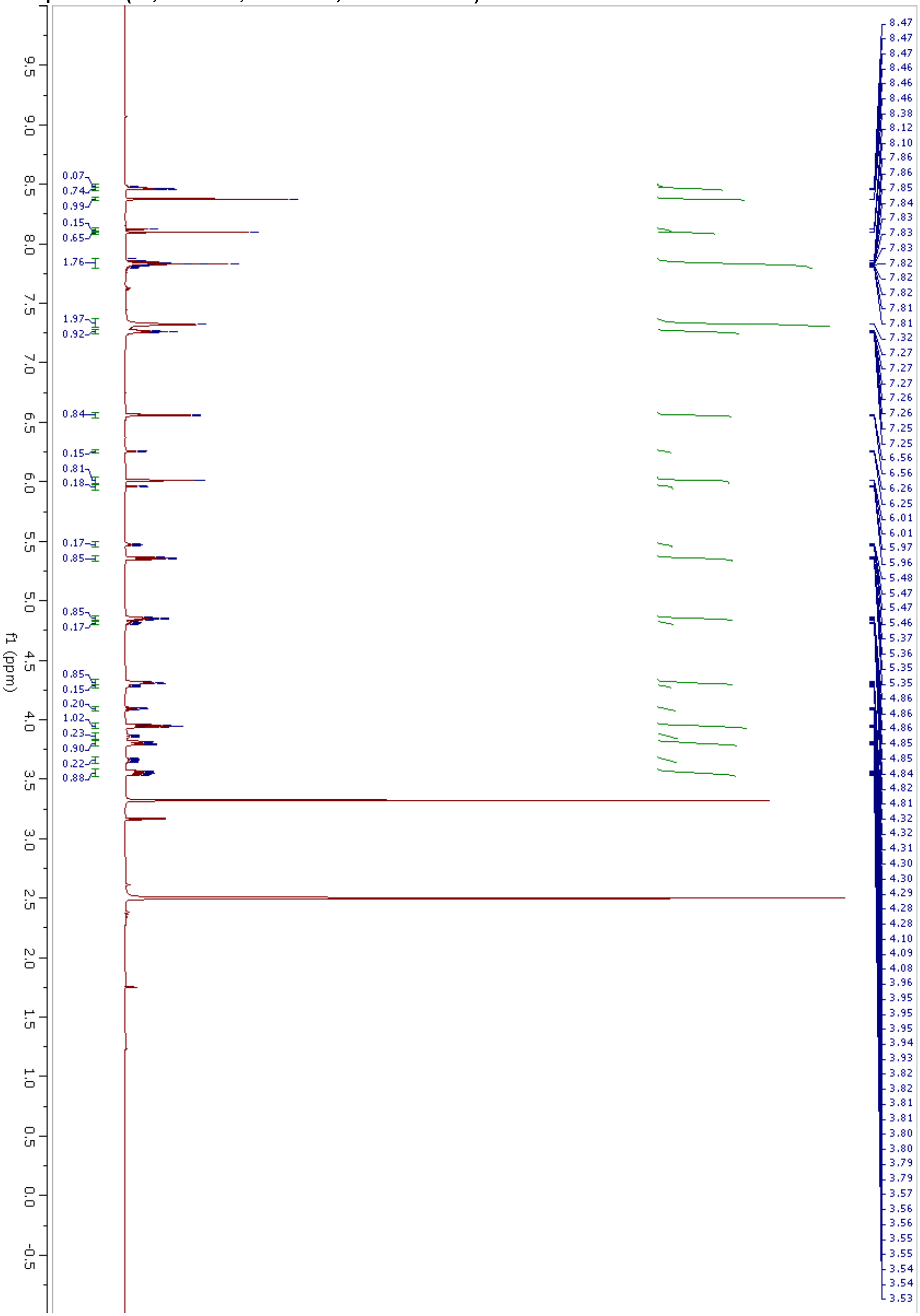

Compound 4 ($^{13}C\{^{1}H\}$, 151 MHz, DMSO-d6, standard view)

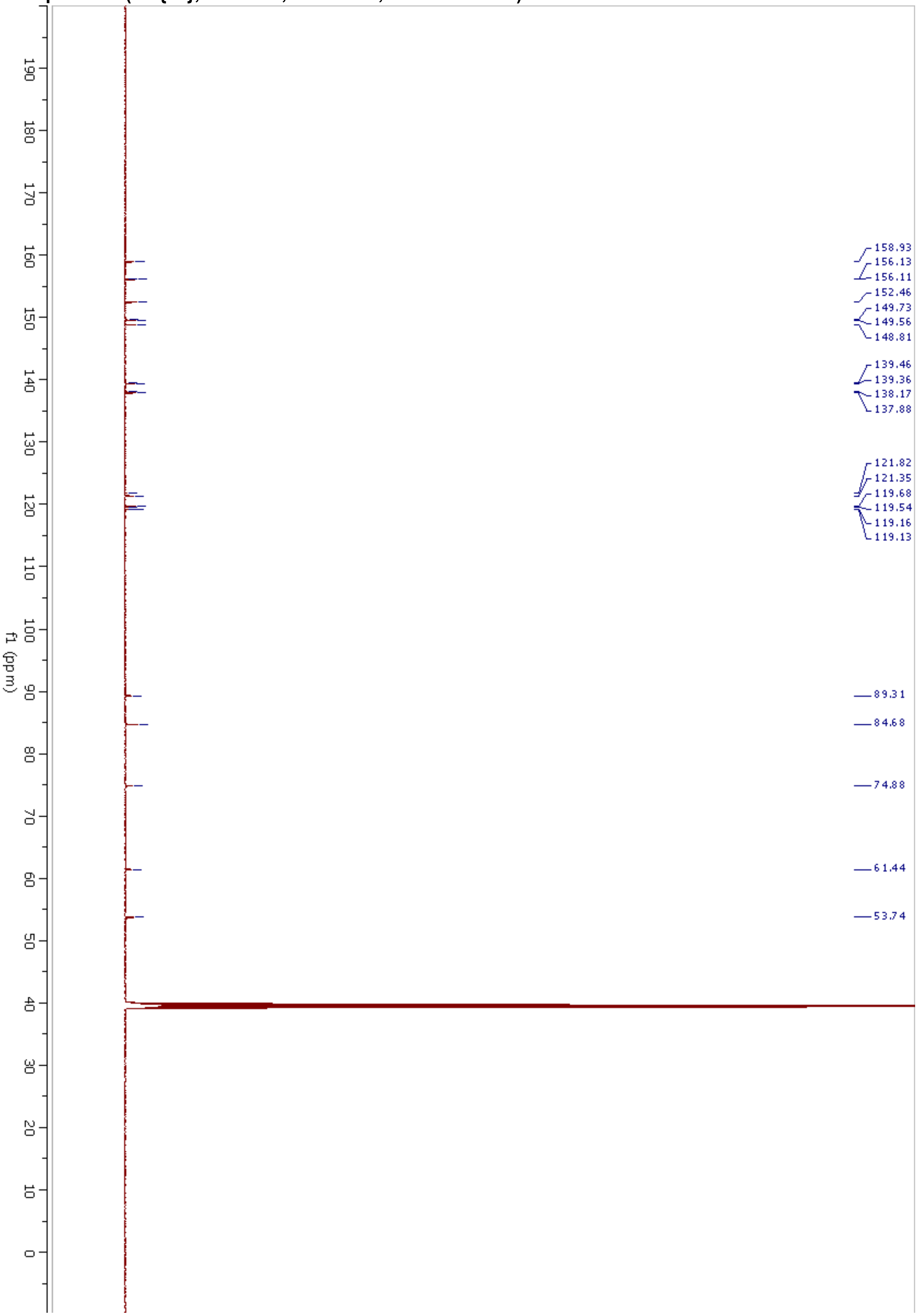

Figure S1: Variable Temperature ^{1}H NMR Studies for Compound **4**

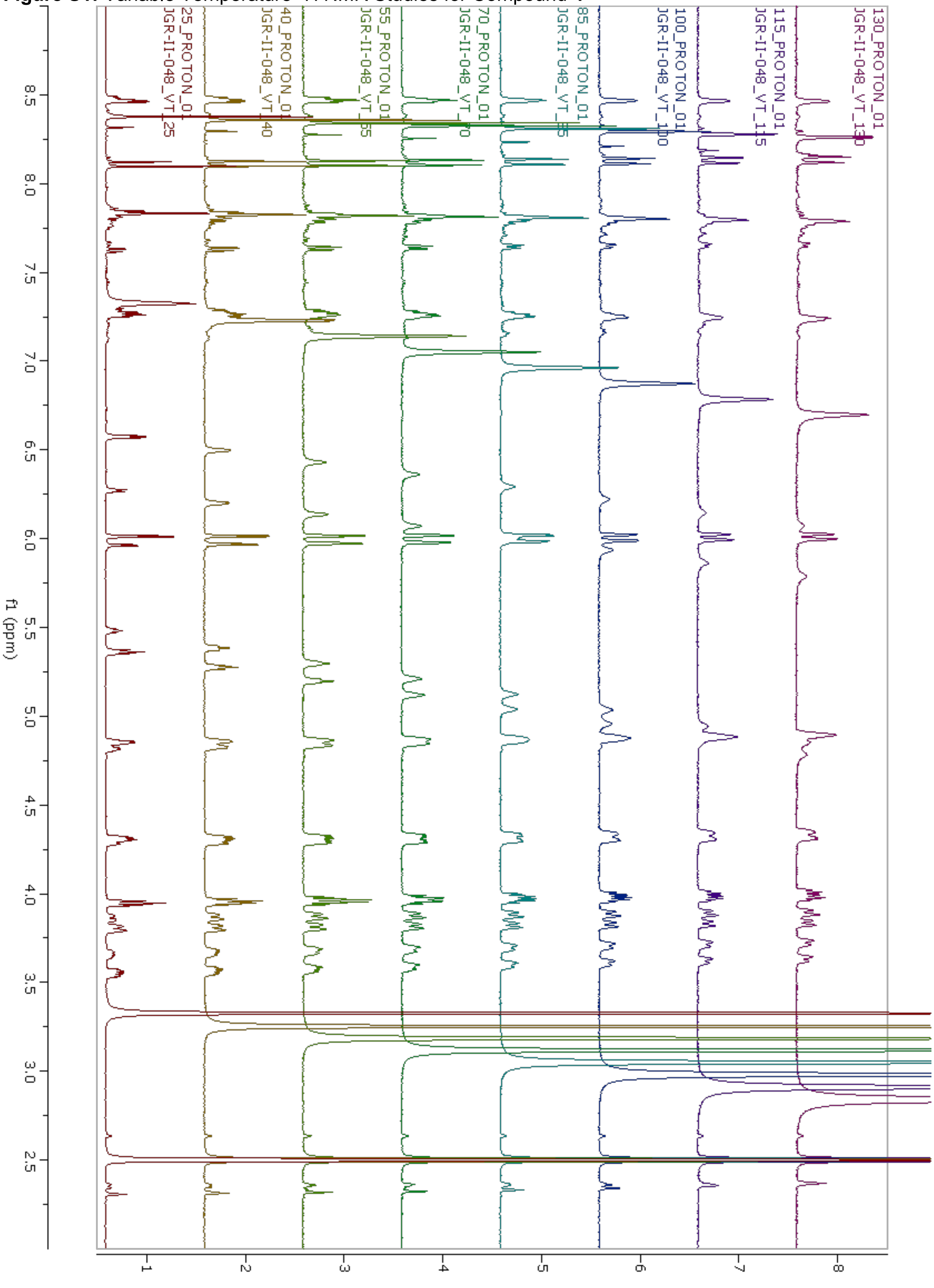

Tetrakis(triethylammonium) ((2*R*,3*S*,4*S*,5*R*)-5-(6-amino-9*H*-purin-9-yl)-4-hydroxy-3-(pyridin-2-yldisulfaneyl)tetrahydrofuran-2-yl)methyl triphosphate (5)

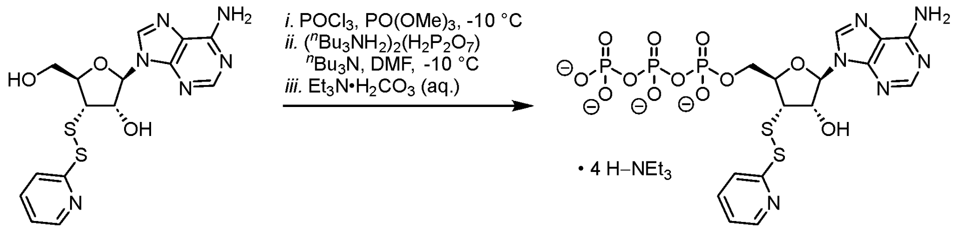

Preparation of $(^nBu_3NH_2)_2(H_2P_2O_7)$, 0.5 M in DMF:[3] Dowex® 50WX8 was added to a column and washed with MeOH until colorless and H_2O until the filtrate was pH ~7. A solution of tetrasodium pyrophosphate decahydrate (670.3 mg, 1.5 mmol, 1.0 equiv) in water (15 mL) was loaded onto the resin and allowed to slowly drip into a solution of freshly distilled $^nBu_3N$ (0.71 mL, 3.0 mmol, 2.0 equiv) and EtOH (6 mL) at 0 °C. The resin was washed with water (3 mL). The resulting mixture was concentrated *in vacuo* at 28 °C and azeotropically dried with EtOH (15 mL) thrice. The resulting solid was diluted with dry DMF (10 mL), concentrated *in vacuo* at 28 °C, immediately placed under an atmosphere of $Ar_{(g)}$, and sealed with a rubber septum. The mixture was transferred to a flame-dried pear-shaped flask charged with freshly activated 3 Å molecular sieves and diluted with DMF until the total volume was 3 mL. The solution was stored at -20 °C.

Preparation of triethylammonium bicarbonate (TEAB) buffer, 2.0 M in water:[3] A solution of triethylamine (560 mL) and water (1.0 L) was vigorously stirred at 0 °C. A separate 1 L round-bottomed flask was filled with crushed dry ice and fitted with a 90° adapter and rubber hose with a glass pipette. Gaseous CO_2 was bubbled through the solution overnight, at which point the solution was pH ~8.

A 50 mL round-bottomed flask equipped with a magnetic stir bar was charged with nucleoside **4** (39.2 mg, 0.1 mmol, 1.0 equiv), sealed with a rubber septum, pierced with a 16-gauge needle, and stored under vacuum over P_2O_5 overnight. The vessel was purged with $Ar_{(g)}$ and charged with freshly distilled trimethyl phosphate (0.25 mL). The suspension was gently warmed with a heat-gun until the starting material was dissolved. The solution was cooled to -10 °C and treated with freshly distilled $POCl_3$ (13.4 µL, 0.12 mmol, 1.2 equiv). After stirring for 2 h, a freshly prepared solution of $(^nBu_3NH_2)_2(H_2P_2O_7)$ (0.5 M in DMF, 0.6 mL, 0.3 mmol, 3.0 equiv), freshly distilled $^nBu_3N$ (0.14 mL, 0.6 mmol, 6.0 equiv), and DMF (1.0 mL) was added to the reaction vessel dropwise. After stirring at -10 °C for an additional 0.5 h, triethylammonium bicarbonate (0.1 M in water, 10 mL) was added. The mixture was warmed to room temperature, stirred for 1 h, and extracted with Et_2O (2 x 5 mL). The aqueous layer was concentrated *in vacuo* at 28 °C.

The crude material was purified by ion exchange chromatography using Sephadex A-25 resin (Solvent A: 0.05 M TEAB, Solvent B: 1.0 M TEAB; CV = 33 mL; 100% A for 1 CV, 100-0% A/B linear gradient over 10 CV, 100% B for 3 CV; flow rate = 8 mL•min^{-1}). The desired nucleotide triphosphate was the latest eluting peak and was collected to provide the title compound as a white solid (34.6 mg, 0.0334 mmol, 33% yield).

The nucleoside triphosphate was further purified by preparative HPLC, as described in the Biochemistry Methods section below.

Physicochemical Properties

HRMS (ESI): Calc. for $[C_{15}H_{18}N_6O_{12}P_3S_2\text{-}H]^-$: 630.9631; Found: 630.9622

Evidence for thioester intermediates: native chemical ligation studies

((2*R*,3*R*,4*S*,5*R*)-4-((Acetylglycyl)oxy)-5-(6-amino-9*H*-purin-9-yl)-3-(pyridin-2-yldisulfaneyl)tetrahydrofuran-2-yl)methyl acetylglycinate (6)

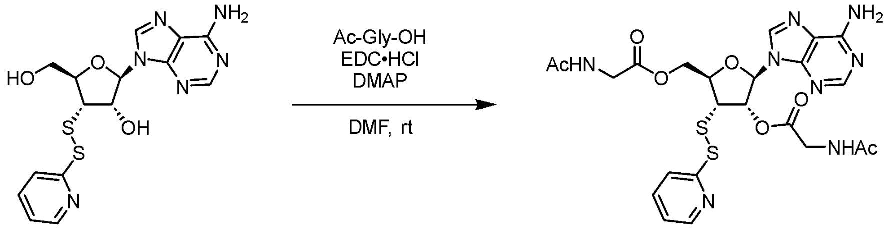

A 1-dram vial equipped with a magnetic stir bar was charged with nucleoside **4** (10.0 mg, 0.0255 mmol, 1.0 equiv), *N*-acetylglycine (7.5 mg, 0.0638 mmol, 2.5 equiv), EDC•HCl (12.2 mg, 0.0638 mmol, 2.5 equiv), DMAP (7.5 mg, 0.0638 mmol, 2.5 equiv), and DMF (0.2 mL). After stirring at room temperature for 1 h, the mixture was diluted with brine and extracted with 3:1 $CHCl_3$/*iso*-propanol (3 x 5 mL). The combined organic extracts were dried over sodium sulfate, filtered, and concentrated *in vacuo*.

The crude mixture was purified by RP-FCC (Biotage® Sfär C18 D – Duo 100 Å 30 µm, 12 g, CV = 45 mL; 0-10% MeCN/H_2O linear gradient for 1 CV, 10-40% MeCN/H_2O linear gradient over 10 CV, 40-100% MeCN/H_2O linear gradient for 2 CV, 100% MeCN for 1 CV, 5% additive of 2% (v/v) aqueous formic acid; flow rate = 25 mL•min^{-1}). A second purification (Biotage® Sfär C18 D – Duo 100 Å 30 µm, 12 g, CV = 45 mL; 0-10% MeCN/H_2O linear gradient for 1 CV, 10-45% MeCN/H_2O linear gradient over 15 CV, 45-100% MeCN/H_2O linear gradient for 1 CV, 100% MeCN for 1 CV; flow rate = 25 mL•min^{-1}) was required to provide the title compound as a white powder (4.7 mg, 0.008 mmol, 31% yield)

<u>Physicochemical Properties</u>

white powder

^{1}H NMR (600 MHz, DMSO-d6) δ 8.49 (ddd, *J* = 4.8, 1.9, 0.9 Hz, 1H), 8.41 (t, *J* = 5.8 Hz, 1H), 8.37 (s, 1H), 8.24 (t, *J* = 5.9 Hz, 1H), 8.07 (s, 1H), 7.85 (td, *J* = 7.7, 1.9 Hz, 1H), 7.76 (dd, *J* = 8.1, 1.1 Hz, 1H), 7.29 (ddd, *J* = 7.4, 4.8, 1.0 Hz, 1H), 6.20 (d, *J* = 2.3 Hz, 1H), 5.97 (dd, *J* = 6.1, 2.3 Hz, 1H), 4.57 – 4.50 (m, 2H), 4.45 (ddd, *J* = 9.6, 5.4, 2.4 Hz, 1H), 4.28 (dd, *J* = 12.3, 5.5 Hz, 1H), 4.01 (dd, *J* = 17.7, 5.9 Hz, 1H), 3.95 (dd, *J* = 17.7, 5.8 Hz, 1H), 3.70 (dd, *J* = 17.5, 5.9 Hz, 1H), 3.63 (dd, *J* = 17.5, 5.9 Hz, 1H), 1.88 (s, 3H), 1.83 (s, 3H).

^{13}C NMR (151 MHz, DMSO-d6) δ 169.9, 169.8, 169.7, 169.2, 157.8, 155.3, 151.6, 149.8, 148.6, 140.2, 137.9, 121.8, 120.3, 118.9, 87.1, 81.0, 76.8, 63.5, 51.4, 40.4, 39.9, 22.21, 22.19.

HRMS (ESI): Calc. for $[C_{23}H_{26}N_8O_7S_2+H]^+$: 591.1439; Found: 591.1451

Compound 6 (^{1}H, 600 MHz, DMSO-d6, standard view)

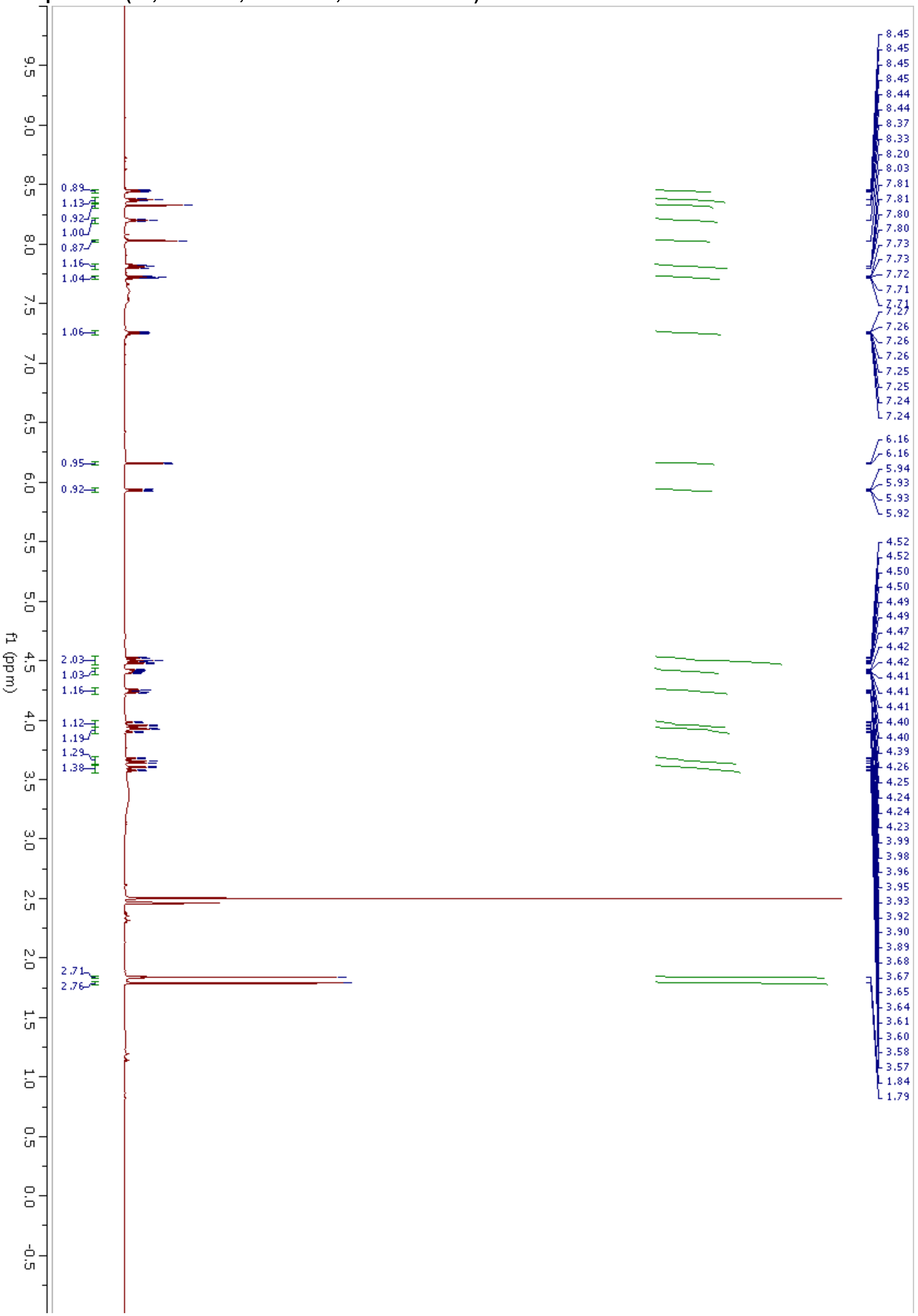

Compound 6 ($^{13}C\{^{1}H\}$, 151 MHz, DMSO-d6, standard view)

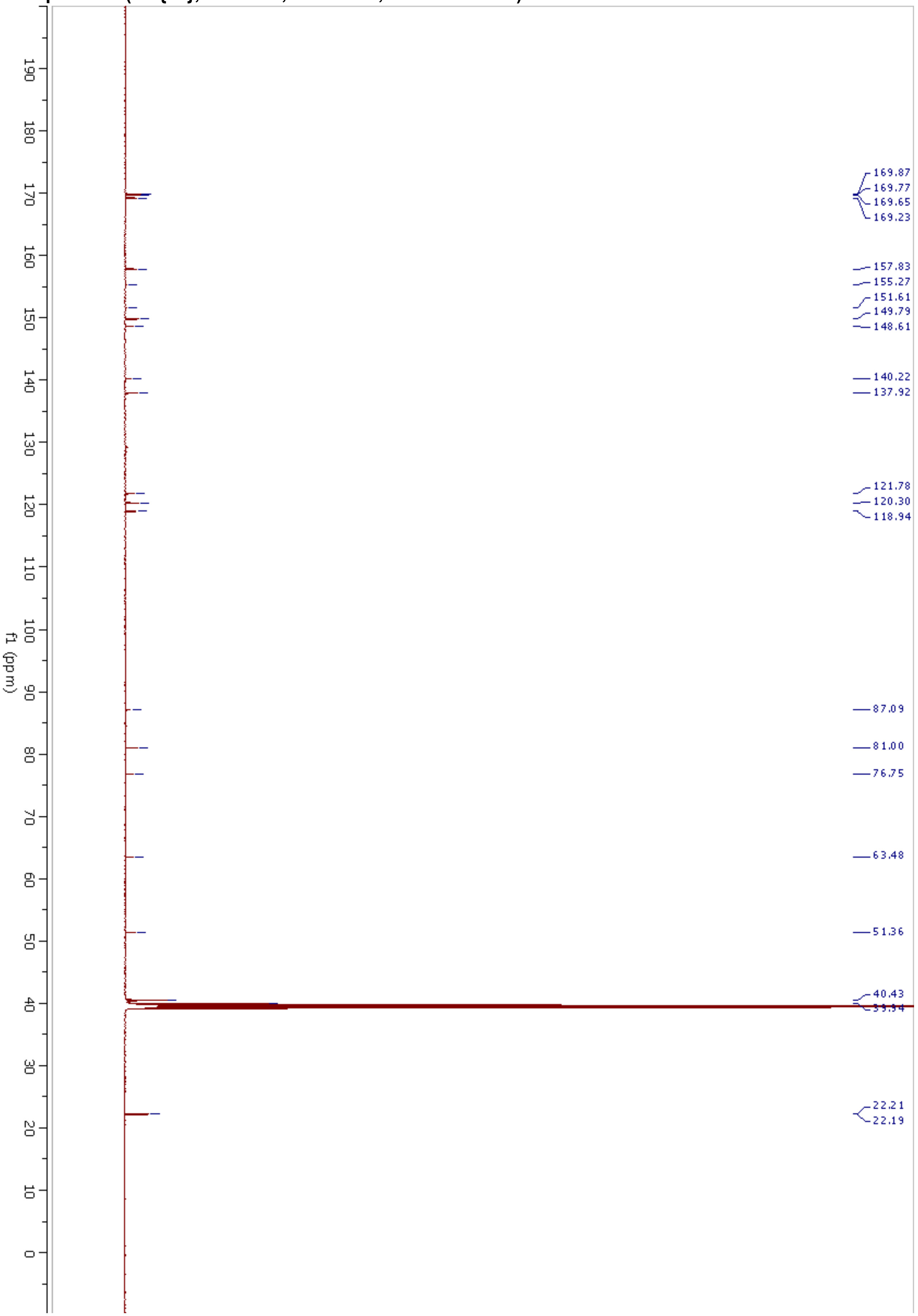

^{1}H/^{1}H COSY NMR Spectrum of Compound 6 (600 MHz, $CDCl_3$)

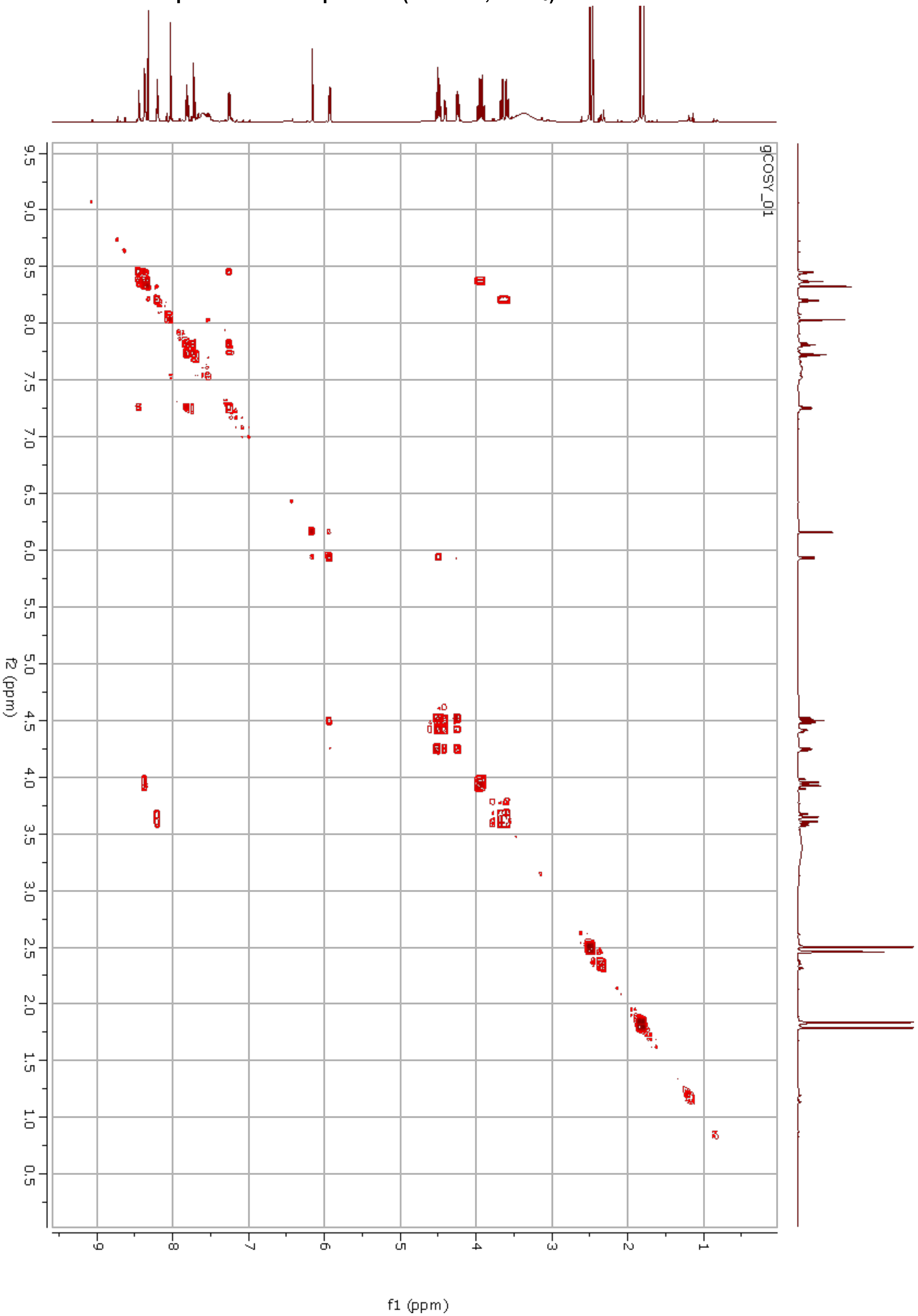

$^{1}H/^{13}C$ HSQC NMR Spectrum of Compound 6 (600/151 MHz, $CDCl_3$)

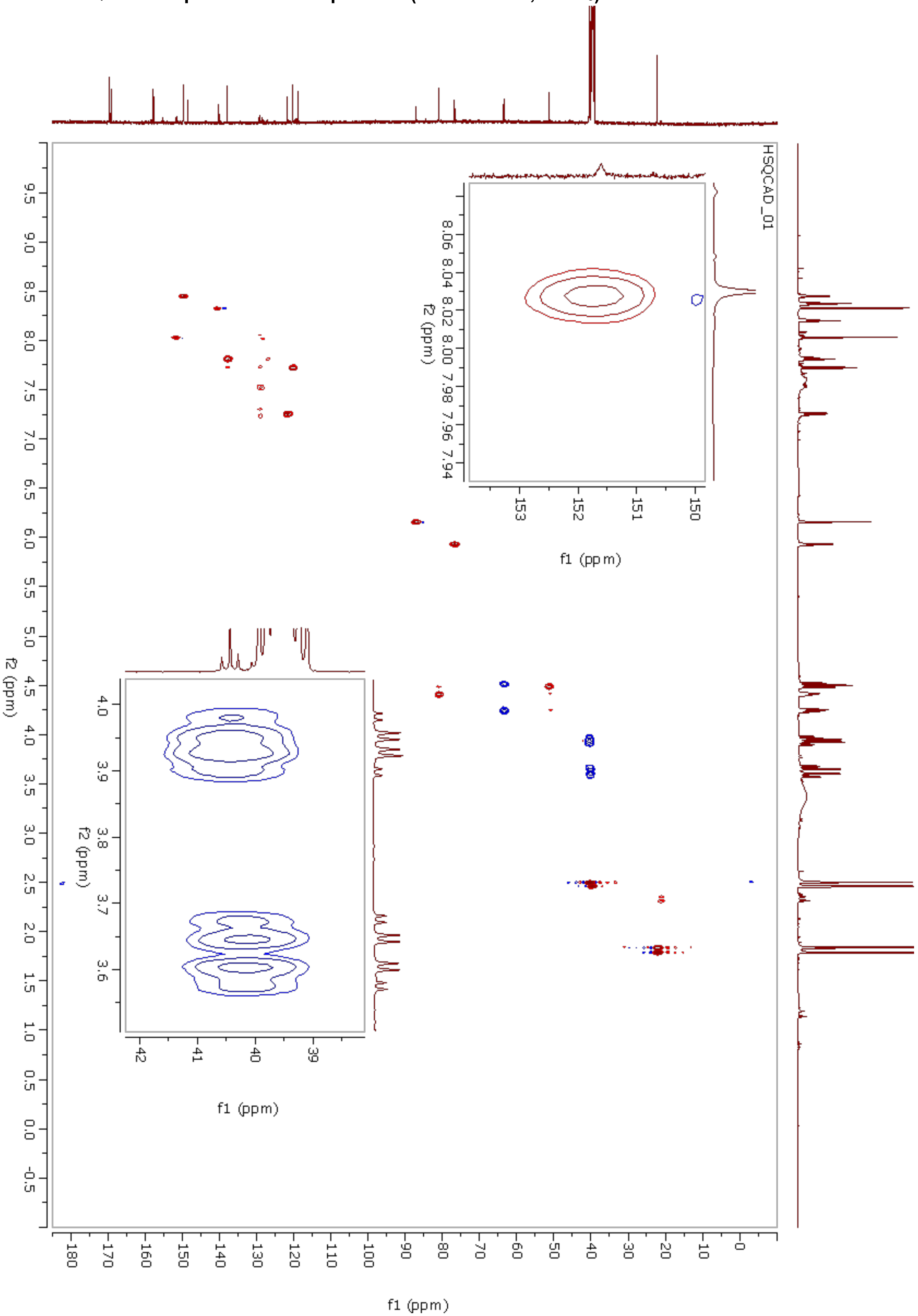

^{1}H/^{13}C HMBC NMR Spectrum of Compound 6 (600/151 MHz, $CDCl_3$)

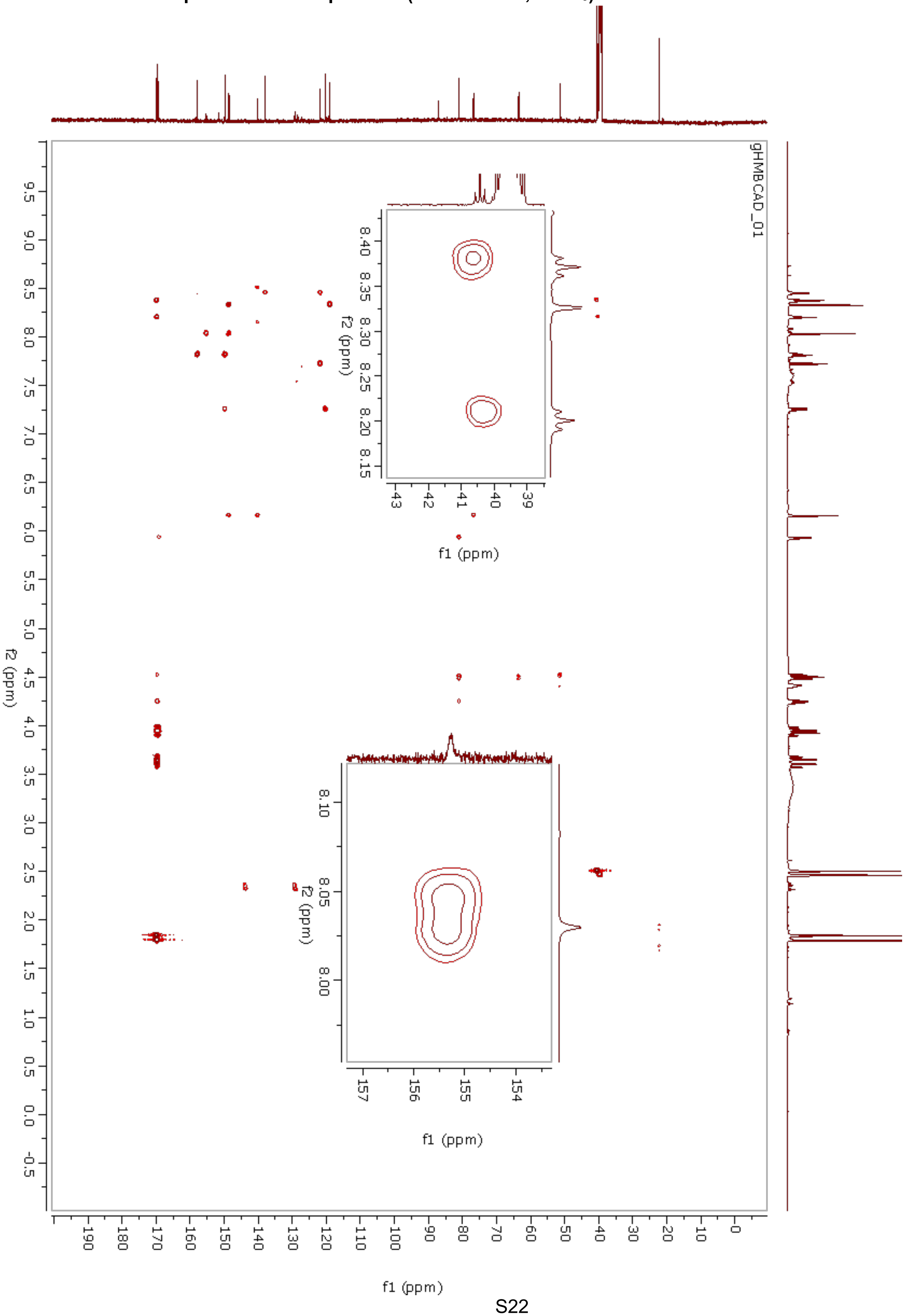

(2*R*,3*R*,4*R*,5*R*)-2-(((Acetylglycyl)oxy)methyl)-5-(6-amino-9*H*-purin-9-yl)tetrahydrofuran-3,4-diyl bis(2-acetamidoacetate) (10)

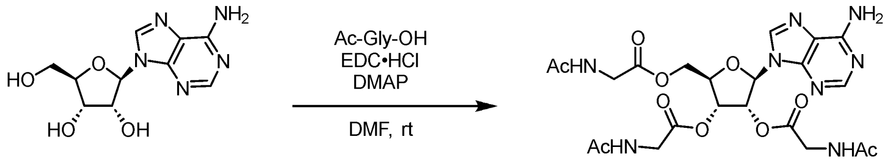

A scintillation vial equipped with a magnetic stir bar was charged with adenosine (133.6 mg, 0.5 mmol, 1.0 equiv), *N*-acetylglycine (257.6 mg, 2.2 mmol, 4.4 equiv), EDC•HCl (421.7 mg, 2.2 mmol, 4.4 equiv), DMAP (268.8 mg, 2.2 mmol, 4.4 equiv), and DMF (2.0 mL). After stirring at room temperature for 1 h, the mixture was diluted with brine and extracted with $CHCl_3$/*iso*-propanol (3:1 v/v, 3 x 10 mL). The combined organic extracts were dried over sodium sulfate, filtered, and concentrated *in vacuo*. The crude mixture was purified by RP-FCC (SNAP Ultra C18 60 g, CV = 90 mL; 0-10% MeCN/H_2O linear gradient for 10 CV; flow rate = 50 mL•min^{-1}) to provide the title compound as a white powder (71.1 mg, 0.126 mmol, 25% yield). The deliquescent material was stored in a desiccator.

<u>Physicochemical Properties</u>

hygroscopic white powder

^{1}H NMR (600 MHz, D_2O) δ 8.30 (d, *J* = 1.3 Hz, 1H), 8.25 (d, *J* = 1.4 Hz, 1H), 6.36 (dd, *J* = 5.5, 1.2 Hz, 1H), 5.94 (td, *J* = 5.5, 1.3 Hz, 1H), 5.77 (ddd, *J* = 5.7, 4.5, 1.3 Hz, 1H), 4.71 – 4.69 (m, 1H), 4.57 (dd, *J* = 12.5, 2.9 Hz, 1H), 4.51 (dd, *J* = 12.5, 3.9 Hz, 1H), 4.13 – 4.12 (m, 2H), 4.04 – 3.98 (m, 3H).

^{13}C NMR (151 MHz, D_2O) δ 174.8, 174.6, 174.5, 171.0, 170.3, 170.0, 155.6, 153.0, 148.7, 139.8, 118.7, 85.4, 79.8, 73.9, 71.0, 63.6, 41.2, 41.0, 40.9, 21.43, 21.39, 21.3

HRMS (ESI): Calc. for $[C_{22}H_{28}N_8O_{10}+H]^+$: 565.2001; Found: 565.1999

Compound 10 (^{1}H, 600 MHz, D_2O, standard view)

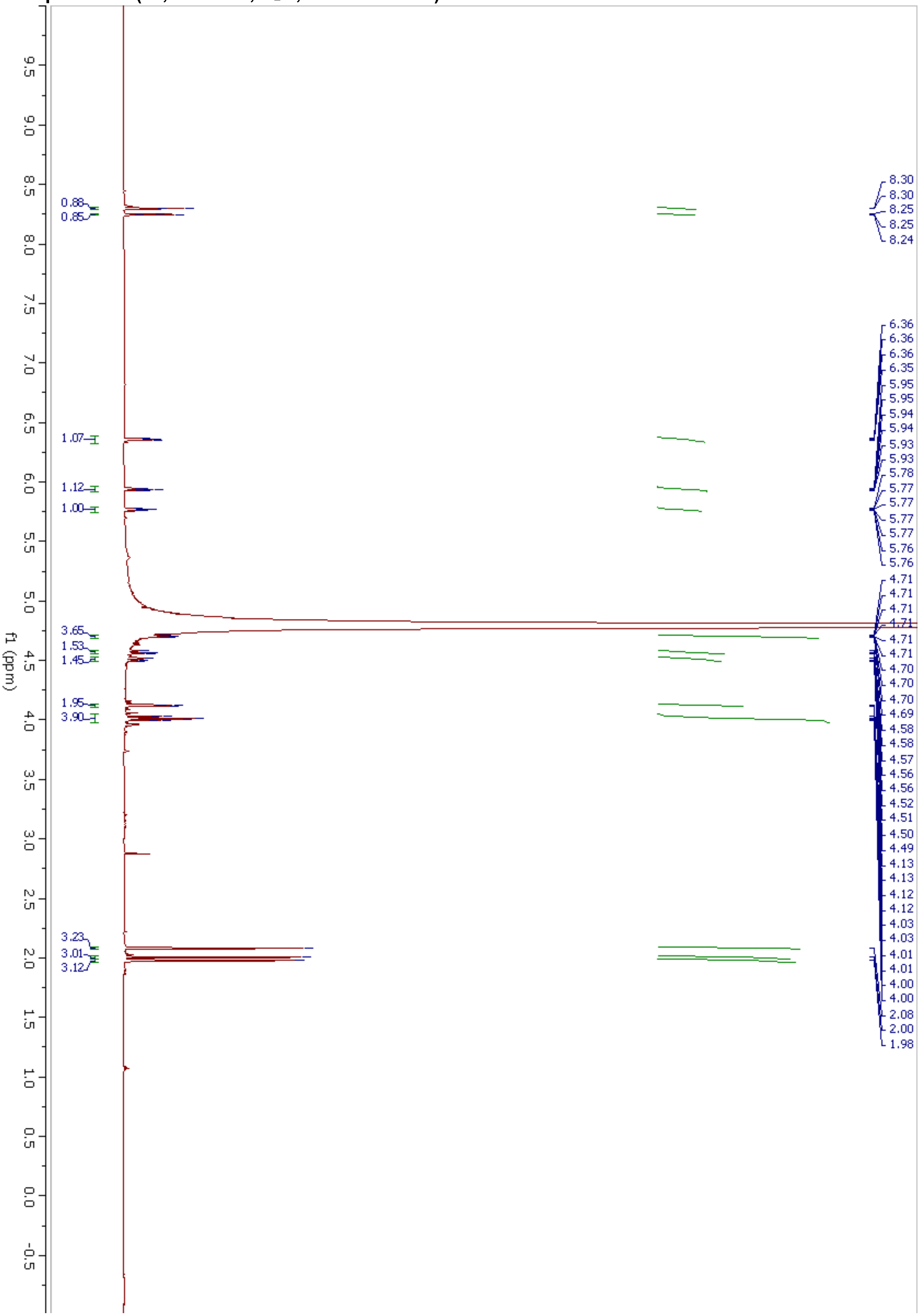

Compound 10 ($^{13}C\{^1H\}$, 151 MHz, D_2O, standard view)

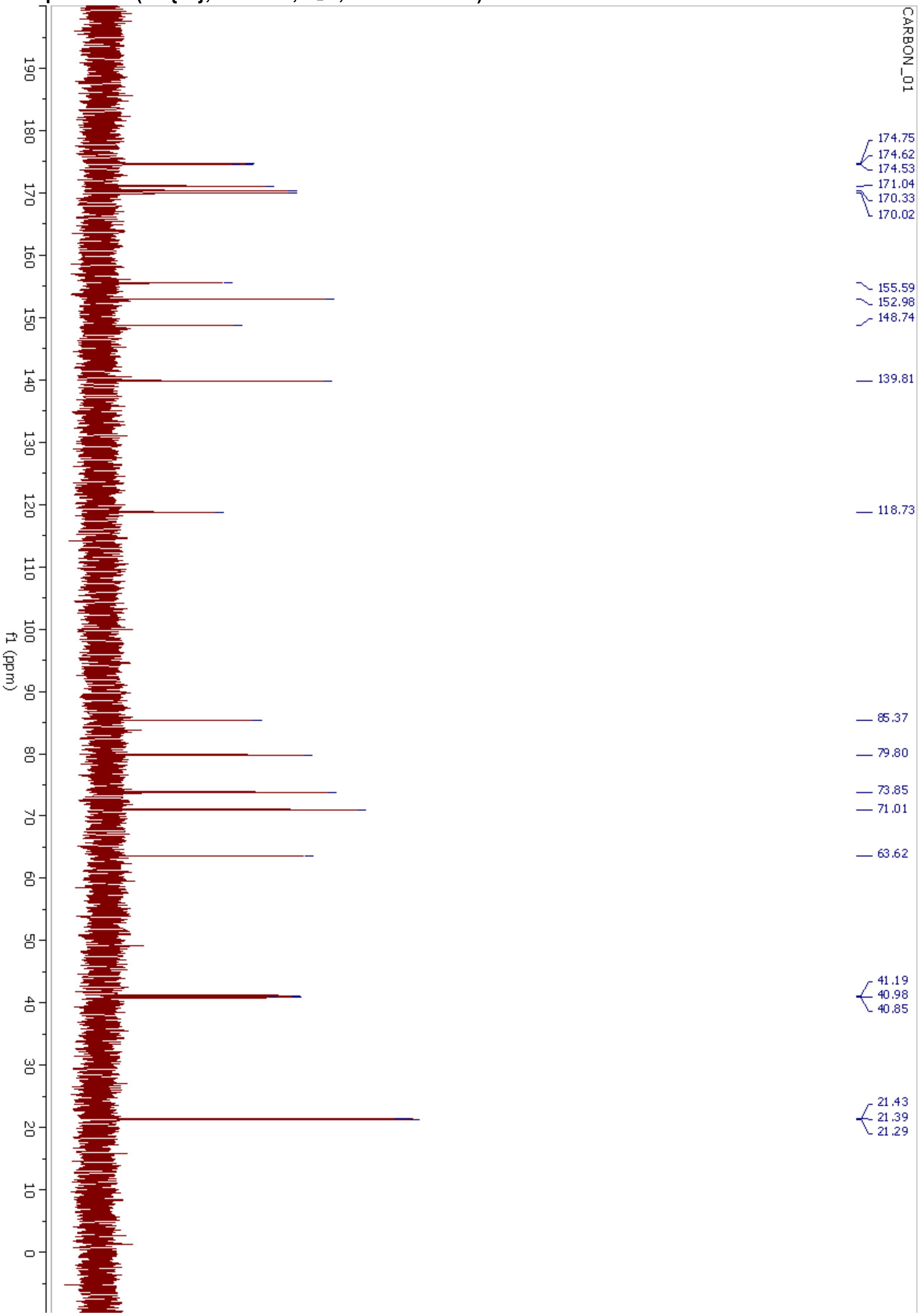

Dimethyl 3,3'-disulfanediyl(2*R*,2'*R*)-bis(2-(2-acetamidoacetamido)propanoate) (S3):

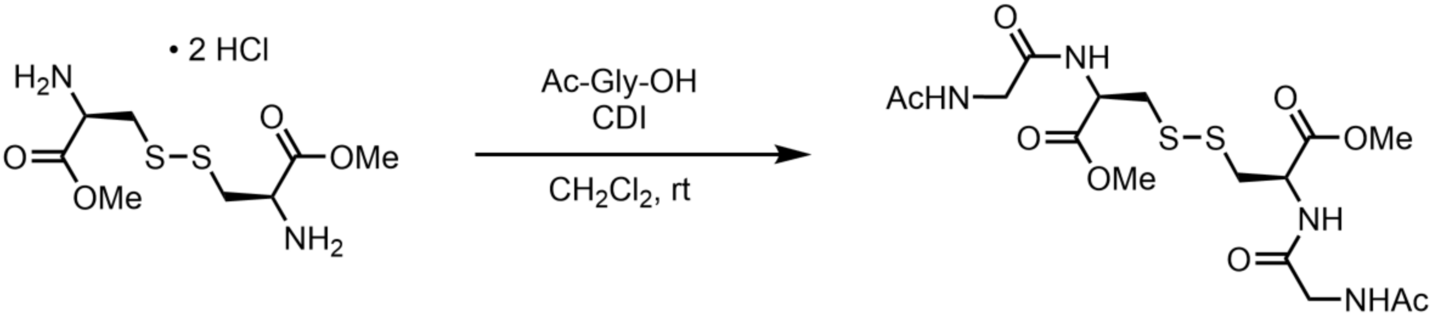

Cystine dimethyl ester bishydrochloride salt was prepared according to a literature procedure.[4]

A scintillation vial equipped with a magnetic stir bar was charged with *N*-acetylglycine (146.4 mg, 1.25 mmol, 2.5 equiv) and CH_2Cl_2 (1.5 mL). 1,1'-Carbonyldiimidazole (202.7 mg, 1.25 mmol, 2.5 equiv) was added in a single portion. The suspension was stirred at room temperature for 0.5 h, by which time the solution had clarified. Cystine dimethyl ester bishydrochloride salt (170.6 mg, 0.5 mmol, 1.0 equiv) was added in a single portion. After stirring for 1 h, the mixture was concentrated *in vacuo* and purified by RP-FCC (SNAP Ultra C18, 60 g, CV = 90 mL; 5% additive of aqueous formic acid [2% v/v] throughout; 0-5% MeCN/H_2O linear gradient for 1 CV; 5-20% MeCN/H_2O linear gradient for 10 CV, 20-100% MeCN/H_2O linear gradient for 0.5 CV, 100% MeCN for 1 CV; flow rate = 50 mL•min^{-1}) to provide the title compound as a white powder (191.8 mg, 0.4111 mmol, 82%).

Physicochemical Properties

white powder

^{1}H NMR (600 MHz, DMSO-d6) δ 8.43 (d, *J* = 7.9 Hz, 1H), 8.09 (t, *J* = 5.9 Hz, 1H), 4.58 (td, *J* = 8.3, 5.2 Hz, 1H), 3.73 (s, 1H), 3.72 (s, 1H), 3.65 (s, 3H), 3.13 (dd, *J* = 13.9, 5.2 Hz, 1H), 2.95 (dd, *J* = 13.9, 8.7 Hz, 1H), 1.85 (s, 3H).

^{13}C NMR (151 MHz, DMSO-d6) δ 170.8, 169.6, 169.3, 52.2, 51.2, 41.6, 38.9, 22.5.

HRMS (ESI): Calc. for $[C_{16}H_{26}N_4O_8S_2+H]^+$: 467.1265; Found: 467.1219

Compound S3 (^{1}H, 600 MHz, DMSO-d6, standard view)

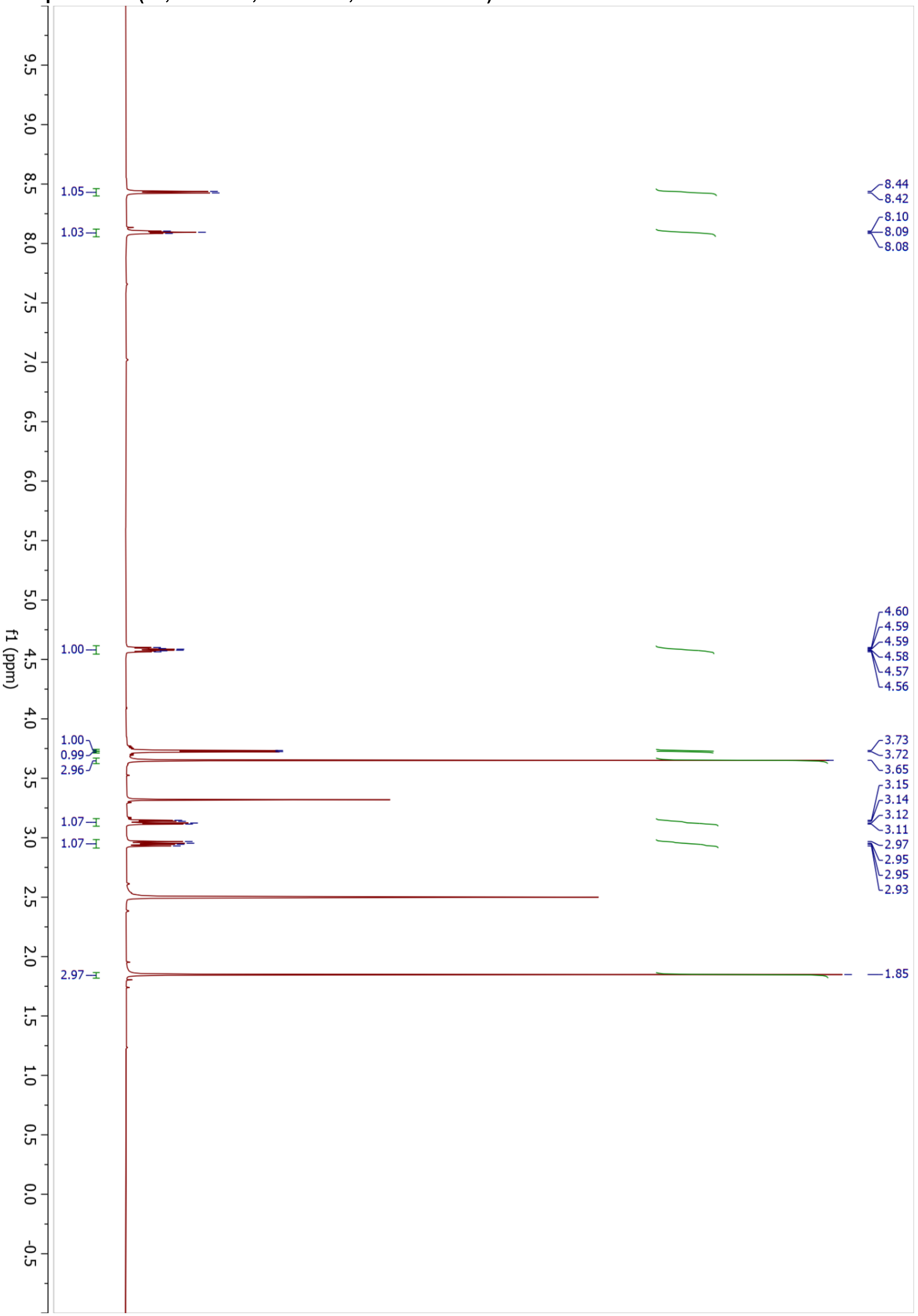

Compound S3 ($^{13}C\{^1H\}$, 151 MHz, DMSO-d6, standard view)

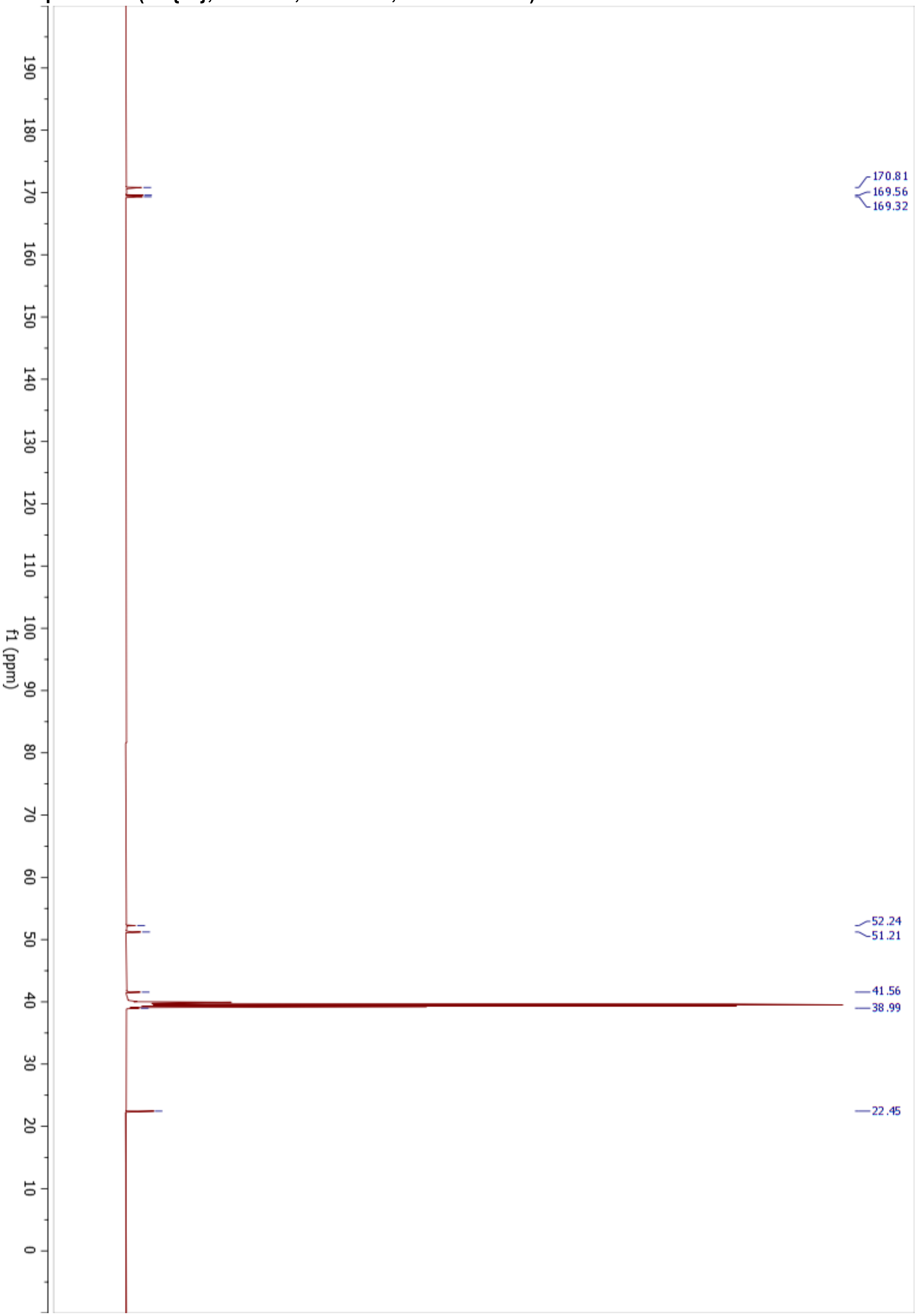

$^{1}H/^{13}C$ HSQC NMR Spectrum of Compound S3 (600/151 MHz, DMSO-d6)

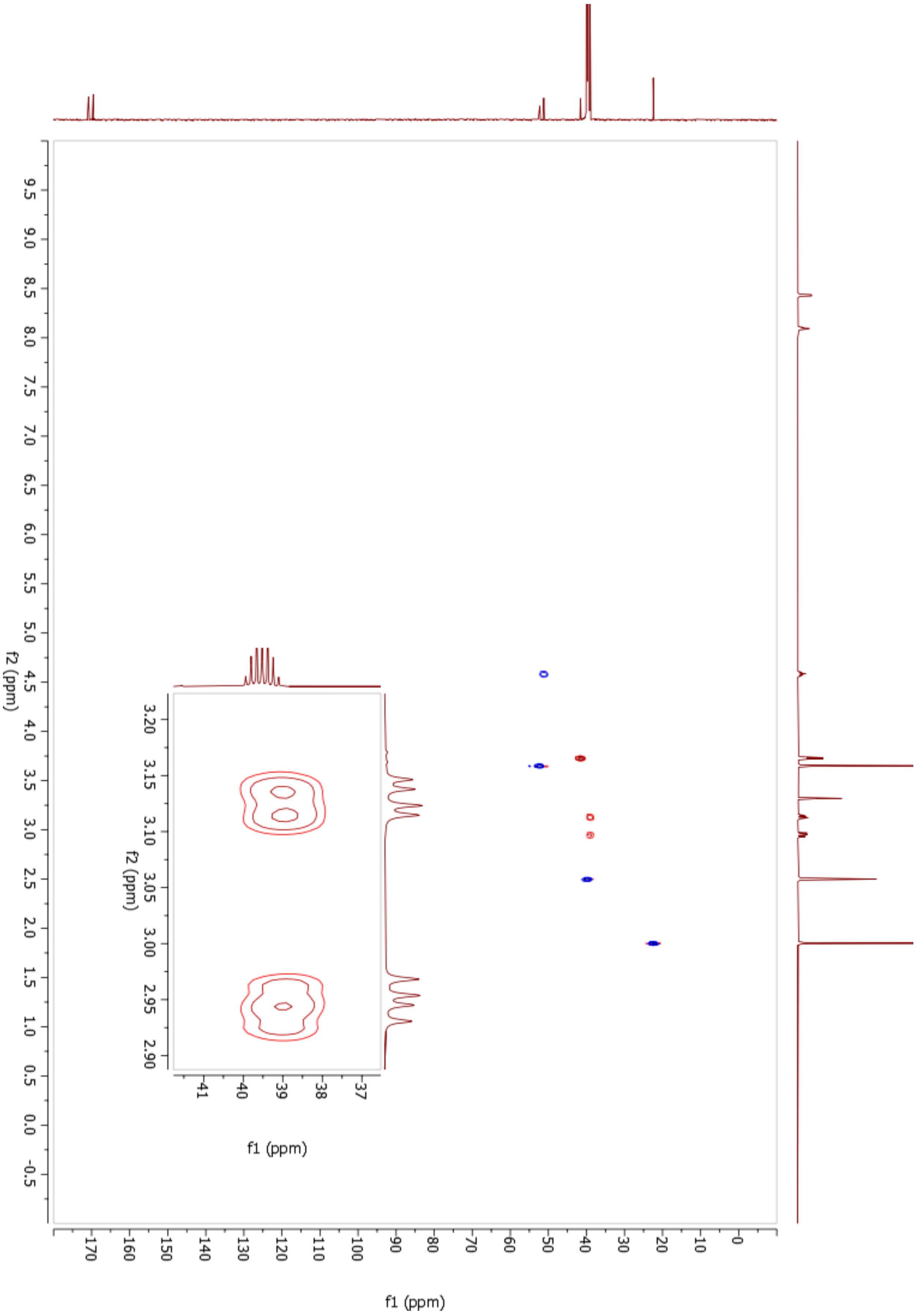

NMR studies on disulfide 6

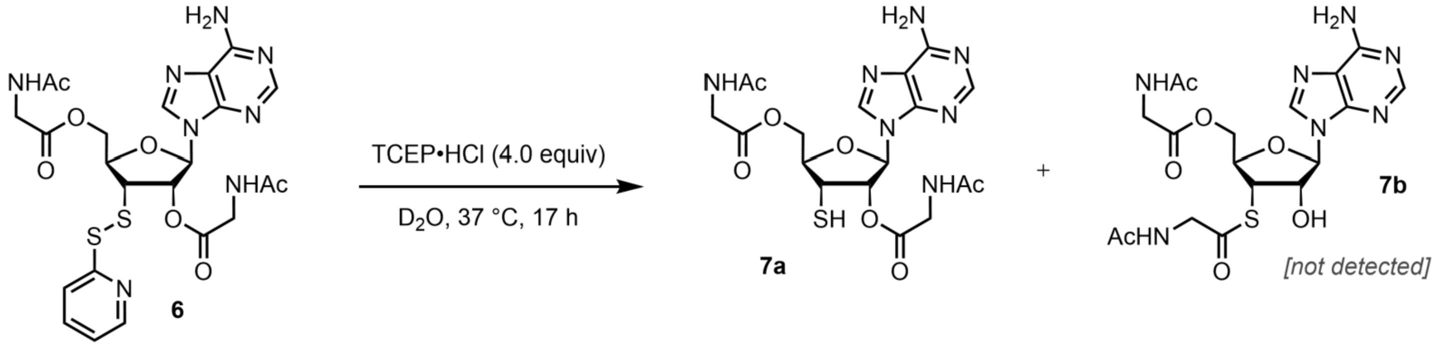

D_2O was degassed by sparging with N_2 for 20 minutes in Schlenk side arm storage flask and sealed. In a nitrogen-filled glove box, a 1-dram vial was charged with disulfide 6 (7.7 mg, 0.013 mmol, 1.0 equiv), TCEP•HCl (14.9 mg, 0.052 mmol, 4.0 equiv), and degassed D_2O. The solution was transferred to a J-Young NMR tube, removed from the glove box, heated at 37 °C for 17 h, and analyzed by NMR. No change was observed in the ^{1}H NMR after heating (Figures S2 and S3). Analysis of the ^{13}C NMR (Figure S4) and HMBC spectra (Figure S5, see FAIR data) did not reveal the presence of any thioester peaks (ca. 190-210 ppm)[5], strongly indicating that thioester **7b** was not present in detectable amounts under these conditions.

Figure S2: ^{1}H NMR (D_2O, 400 MHz, standard view) at t_0, prior to heating

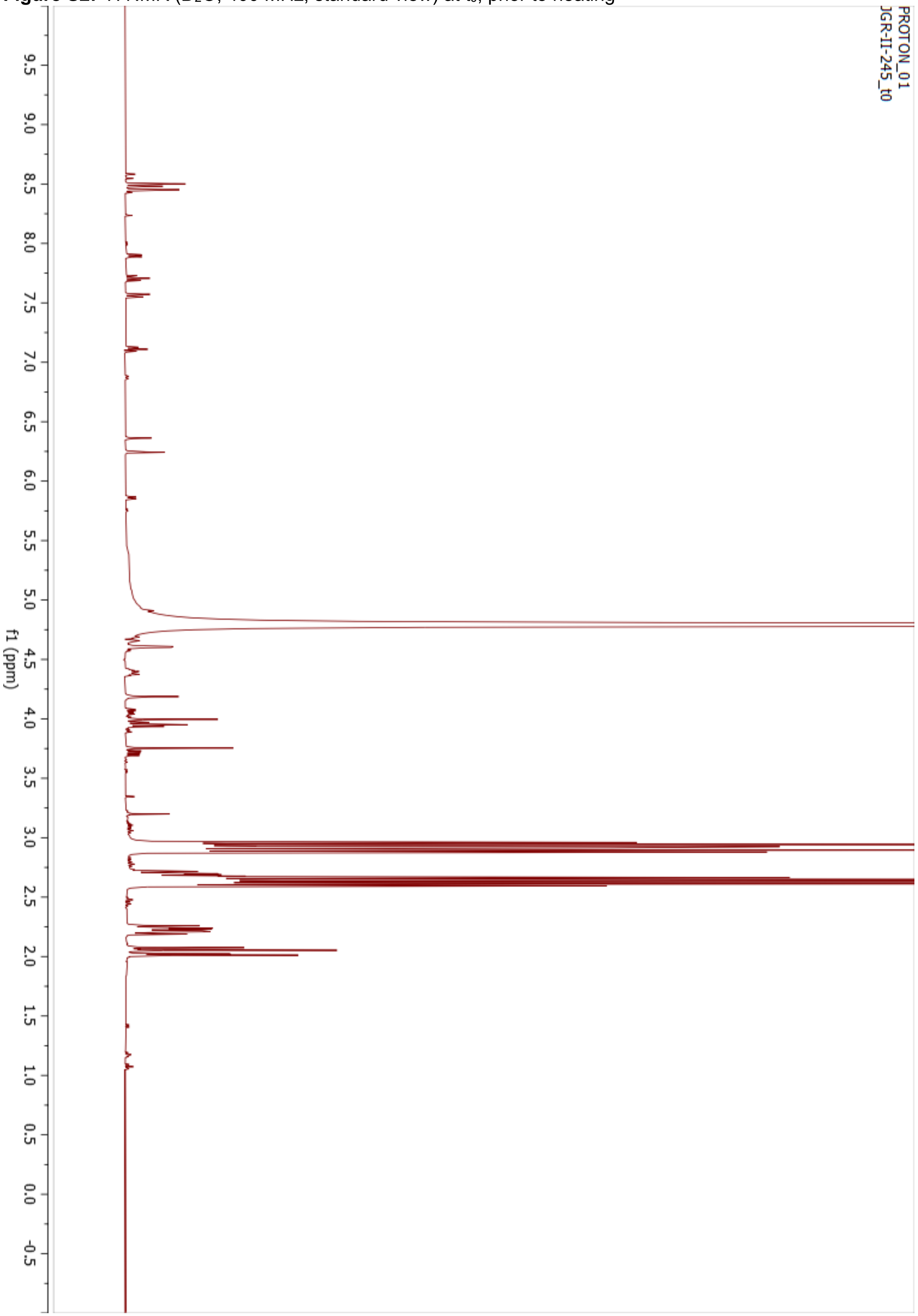

Figure S3: ^{1}H NMR (D_2O, 600 MHz, standard view) after 17 h at 37 °C

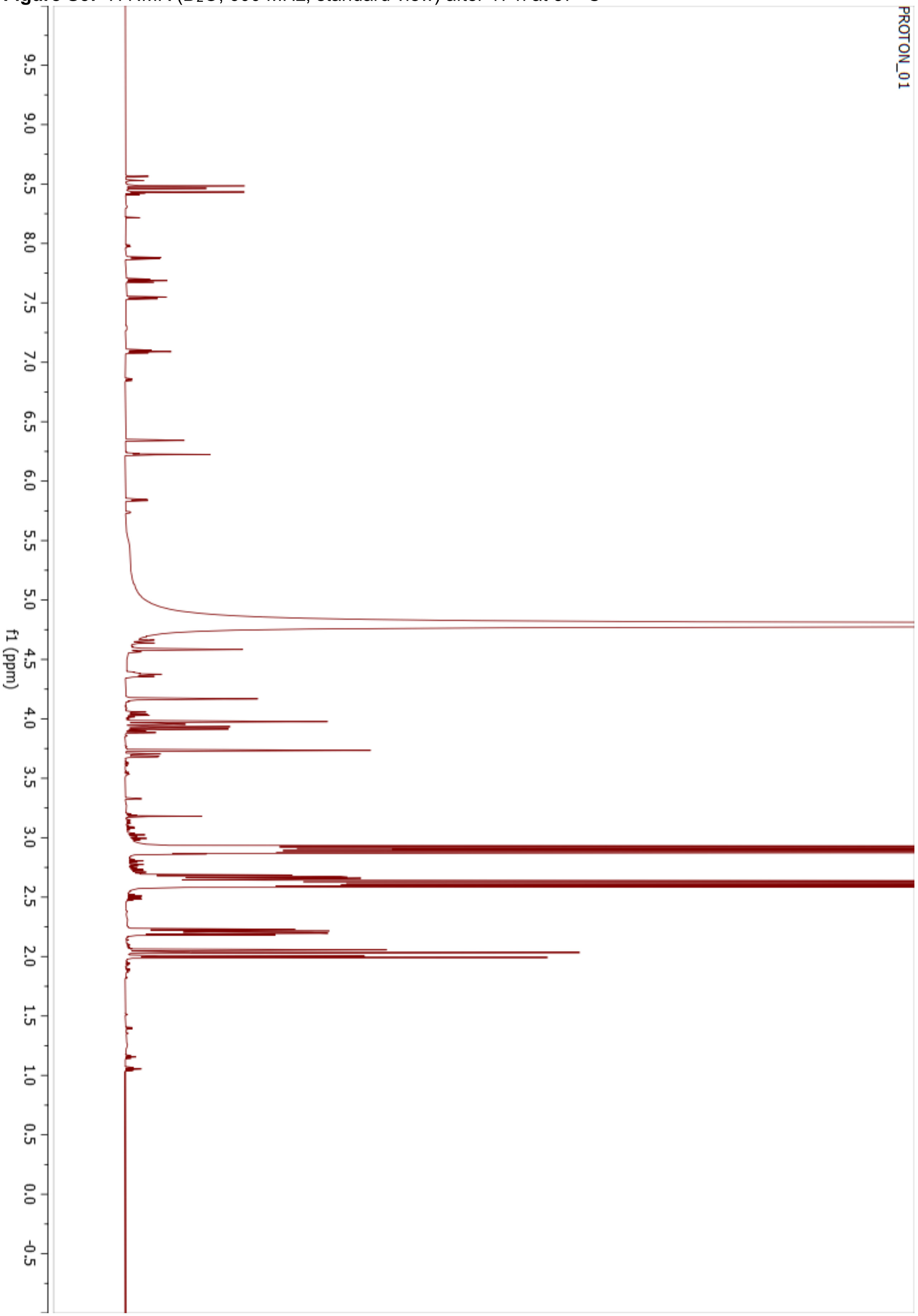

Figure S4: ^{13}C NMR (D_2O, 600 MHz, standard view) after 17 h at 37 °C

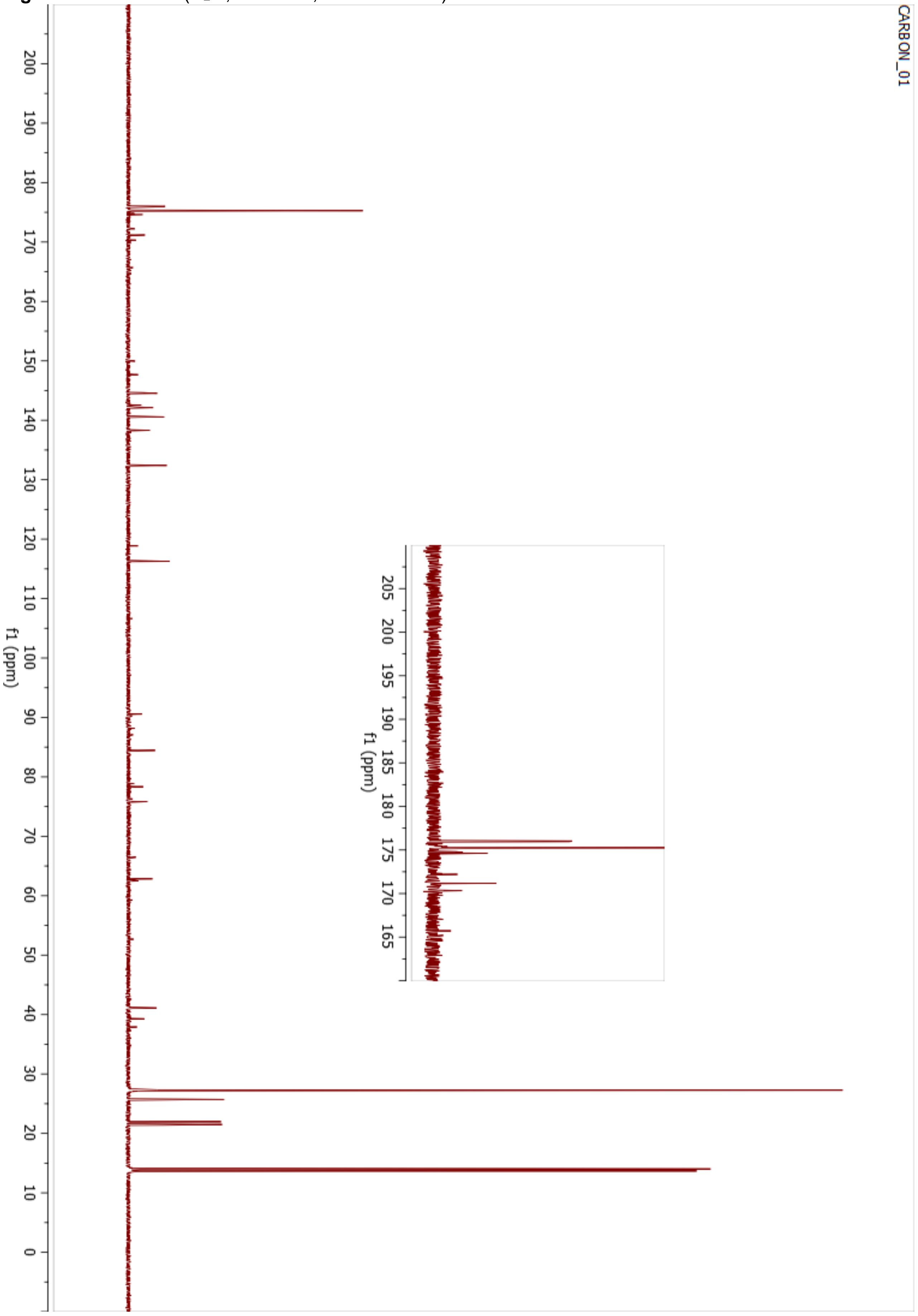

Figure S5: $^{1}H/^{13}C$ HMBC NMR Spectrum after 17 h at 37 °C (600/151 MHz, D_2O)

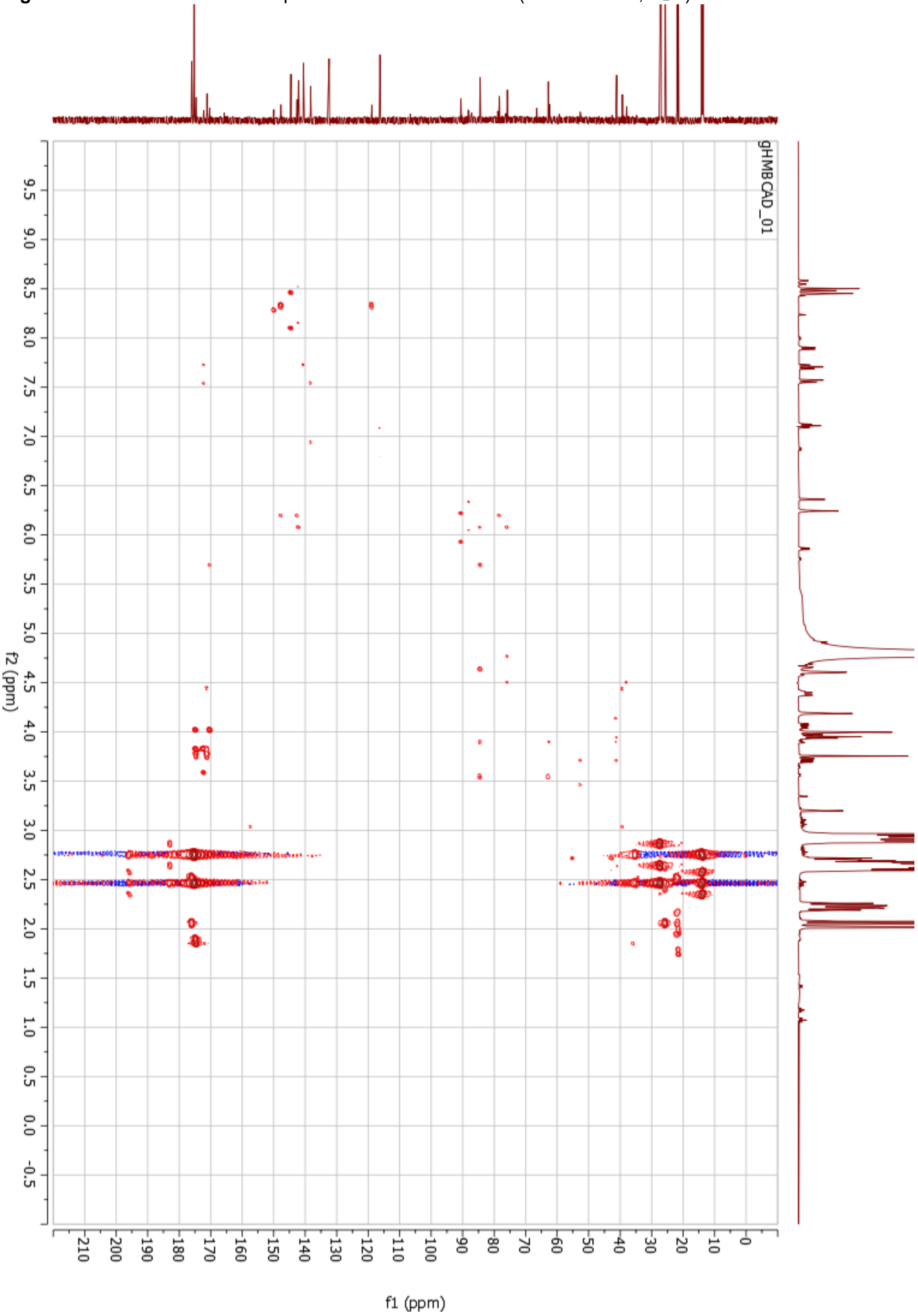

Methods for and analysis of mass spectrometry data for NCL experiment:

(see Fig. 4a,b)

The following solutions were made in 1x PBS buffer (pH 7.4):

Bisacylated disulfide **6** (500 µM)
Triacylated adenosine **10** (500 µM)
TCEP•HCl (825 µM)
H-Cys-OMe•HCl (500 µM)
(Ac-Gly-Cys-OMe)$_2$ **S3** (500 µM)

Calibration curve: An HPLC vial was charged with the solution of disuflide **S3** (200 µL, 100 µmol, 1.0 equiv), pH 7.4 PBS buffer (500 µL), and the solution of TCEP (300 µL, 248 µmol, 2.48 equiv). After standing at 23 °C for 1 h, serial dilution provided solutions of authentic product standard at 100, 25, 10, and 1.0 µM.

Negative control: An HPLC vial was charged with the solution of triacylated adenosine **10** (100 µL, 50.0 µmol, 1.0 equiv), pH 7.4 PBS buffer (500 µL), and the solution of TCEP (200 µL, 165 µmol, 1.65 equiv). After standing at 23 °C for 1 h, the solution of H-Cys-OMe (200 µL, 100 µmol, 2.0 equiv) was added and the reaction was analyzed by UHPLC/HR-MS (*vide infra*).

Test case: An HPLC vial was charged with the solution of bisacylated disulfide **6** (100 µL, 50.0 µmol, 1.0 equiv), pH 7.4 PBS buffer (500 µL), and the solution of TCEP (200 µL, 165 µmol, 1.65 equiv). After standing at 23 °C for 1 h, the solution of H-Cys-OMe (200 µL, 100 µmol, 2.0 equiv) was added and the reaction was analyzed by UHPLC/HR-MS (*vide infra*).

Mass spectrometric measurements were performed with a Shimadzu Scientific Instruments QToF 9030 LC-MS system, equipped with a Nexera LC-40D xs UHPLC, consisting of a CBM-40 Lite system controller, a DGU-405 Degasser Unit, two LC-40D XS UHPLC pumps, a SIL-40C XS autosampler and a Column Oven CTO-40S. UV data was collected with a Shimadzu Nexera HPLC/UHPLC Photodiode Array Detector SPD M-40 in the range of 190 – 800 nm. Mass spectra were subsequently recorded with the quadrupole time-of-flight (QToF) 9030 mass spectrometer.
The samples were held at 4 °C in the autosampler compartment.

Small Molecule
1 µL of each were injected into a sample loop and separated on a Shim-pack Scepter C18-120, 1.9 µm, 2.1x100 mm Column, equilibrated at 40 °C in a column oven.

A binary gradient was used:
Solvent A: Water, HPLC grade Chromasolv, with 0.1% Formic Acid
Solvent B: Acetonitrile, HPLC grade Chromasolv, with 0.1% Formic Acid

Medium:
Flow was held constant at 0.400 mL/min and the composition of the eluent was changed according to the following gradient:
0 to 2 min, held at 95% A, 5% B
2 to 8 min, change to 5% A, 95% B
8 to 10 min, held at 5% A, 95% B
10 to 10.01 min, change to 95% A, 5% B
10.01 to 12 min, held at 95% A, 5% B

The ionization source was run in "ESI" mode, with the electrospray needle held at +4.5kV.
Nebulizer Gas was at 2 L/min, Heating Gas Flow at 10 L/min and the Interface at 300 °C. Dry Gas was at 10 L/min, the Desolvation Line at 250 °C and the heating block at 400 °C.

Mass spectra were recorded in the range of 100 to 2000 m/z in positive ion mode.

Measurements and data post-processing were performed with LabSolutions 5.97 Realtime Analysis and PostRun. Peak integration was performed using the mono-isotopic mass of the protonated and sodiated species (+/-20 ppm) with fixed retention times (+/-0.5 min).

Acknowledgement: *This research made use of the Chemical and Biophysical Instrumentation Center at Yale University (RRID:SCR_021738). Equipment was purchased with funds from Yale University.*

Synthesis of *para*-iodophenylalanine cyanomethyl ester

Cyanomethyl (*S*)-2-((tert-butoxycarbonyl)amino)-3-(4-iodophenyl)propanoate (S5)

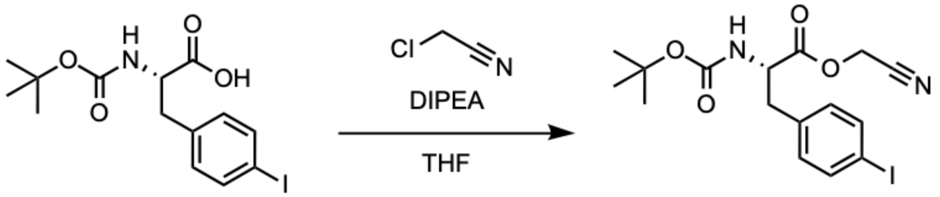

A flame-dried 2.5 mL microwave vial (Biotage) equipped with a magnetic stir bar was charged with Boc-4-iodo-L-phenylalanine **S4** (300.0 mg, 0.767 mmol, 1.0 equiv), anhydrous THF (2.56 mL), DIPEA (160 μL, 0.92 mmol, 1.2 equiv), and 2-chloroacetonitrile (72.8 μL, 0.15 mmol, 1.5 equiv). After stirring at room temperature for 16 h, the crude reaction mixture was concentrated *in vacuo* and resuspended in EtOAc. Silica was added and the solvent was removed under reduced pressure. The crude product was purified by normal phase flash column chromatography (RediSep 4 g, CV = 4.8mL, flow rate = 13 mL/min, silica gel; 0-30% EtOAc/Hex for 10 CV, 30% EtOAc/Hex for 14 CV) to afford Boc-4-iodo-L-phenylalanine cyanomethyl ester **S5** as a white solid (147.9 mg, 0.344 mmol, 45% yield).

<u>Physicochemical Properties</u>

white solid

^{1}H NMR (500 MHz, DMSO-d6) δ 7.64 (d, *J* = 8.2 Hz, 2H), 7.47 (d, *J* = 7.9 Hz, 1H), 7.08 (d, *J* = 8.1 Hz, 2H), 5.01 (s, 2H), 4.26 – 4.22 (m, 1H), 2.99 (dd, *J* = 13.8, 5.2 Hz, 1H), 2.85 (dd, *J* = 13.8, 10.1 Hz, 1H), 1.33 (s, 7H), 1.30 (s, 2H).

^{13}C NMR (126 MHz, DMSO-d6) δ 170.5, 154.9, 138.0, 134.8, 131.2, 113.7, 93.0, 80.7, 54.1, 48.9, 37.6, 28.2.

HRMS (ESI): Calc. for $[C_{16}H_{19}IN_2O_4+H]^+$: 431.0462; Found: 431.0450

Compound S5 (^{1}H, 500 MHz, DMSO, standard view)

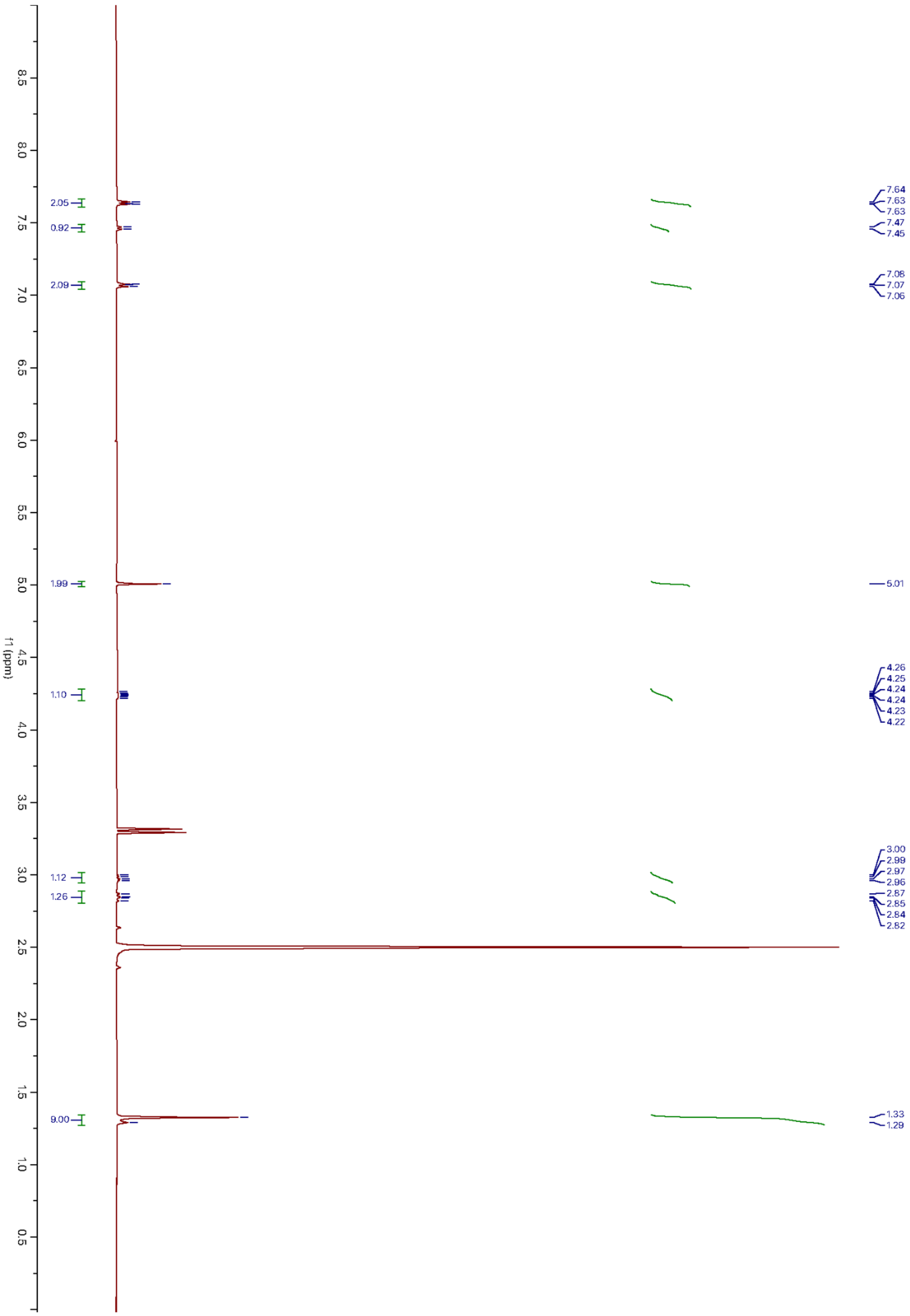

Compound S5 ($^{13}C\{^1H\}$, 126 MHz, DMSO, standard view)

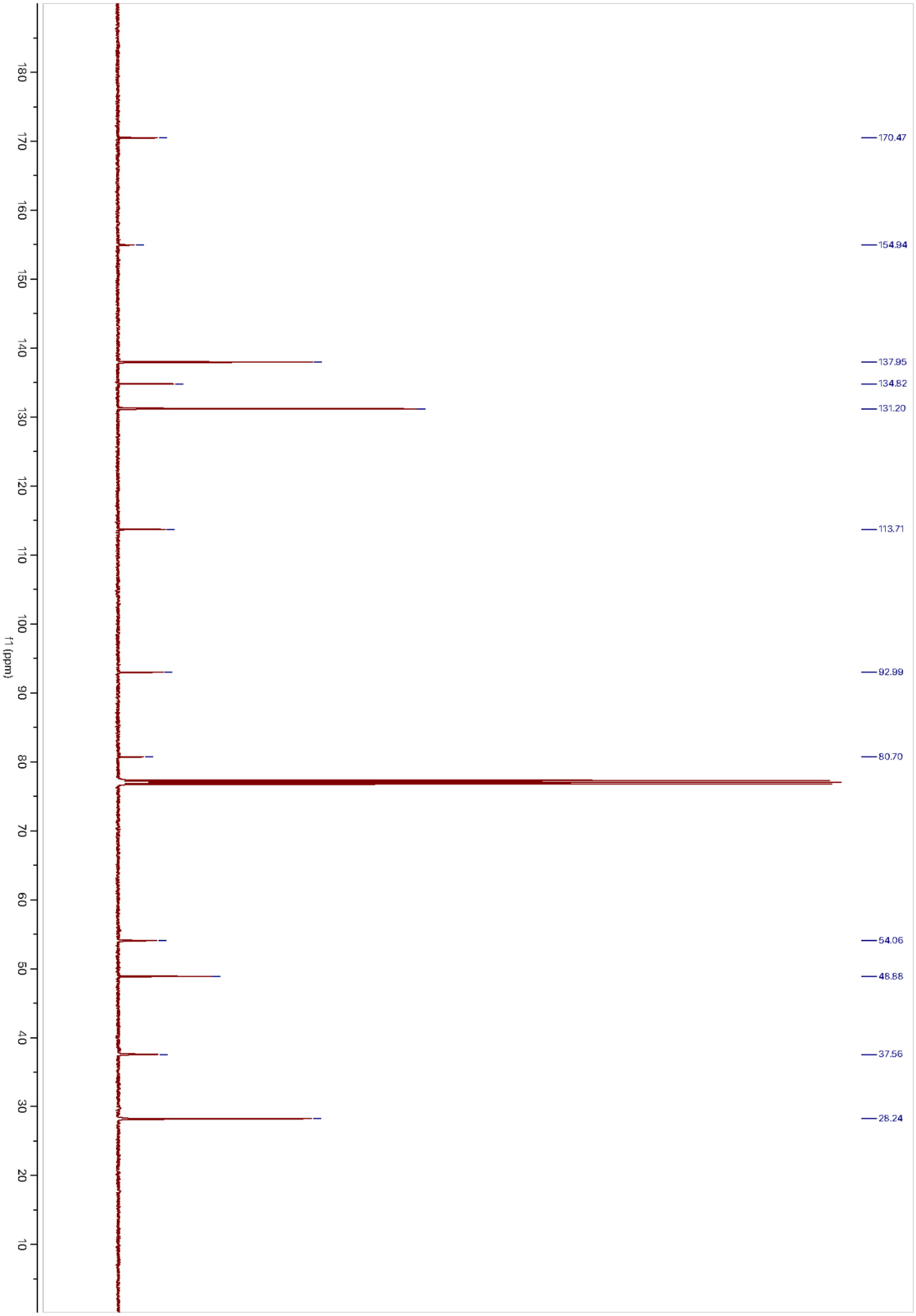

Cyanomethyl (*S*)-2-amino-3-(4-iodophenyl)propanoate hydrochloride (S6)

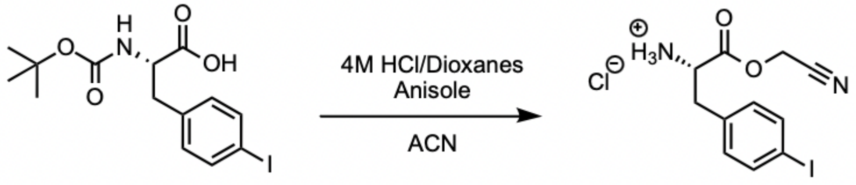

A 1 dram vial equipped with a magnetic stir bar was charged with Boc-4-iodo-L-phenylalanine cyanomethyl ester **S5** (50.0 mg, 0.12 mmol, 1.0 equiv), anhydrous acetonitrile (400 µl), anisole (6.3 µL, 0.058 mmol, 0.5 equiv), and HCl (4.0 M in dioxanes, 61 µL, 0.24 mmol, 2.1 equiv). The solution was stirred at room temperature for 2 h, at which point LC-MS indicated that the reaction was complete, and 5 mL of diethyl ether was added to precipitate the product. The sample was centrifuged at 3000 x G for 5 min and the supernatant decanted. The precipitant was dried under vacuum to provide the title compound **S6** as a white solid.

Physicochemical Properties

white solid

^{1}H NMR (500 MHz, DMSO) δ 8.40 (s, 2H), 7.59 – 7.54 (m, 2H), 6.94 (d, *J* = 8.1 Hz, 2H), 4.99 (dd, *J* = 2.6 Hz, 2H), 4.34 (dd, *J* = 6.6 Hz, 1H), 2.94 (s, 2H).

^{13}C NMR (126 MHz, DMSO) δ 167.0, 137.3, 132.4, 130.3, 113.0, 89.0, 52.5, 48.9, 34.5.

HRMS (ESI): Calc. for $[C_{11}H_{12}IN_2O_4+H]^+$: 330.9938; Found: 331.0008

Compound S6 (^{1}H, 500 MHz, DMSO, standard view)

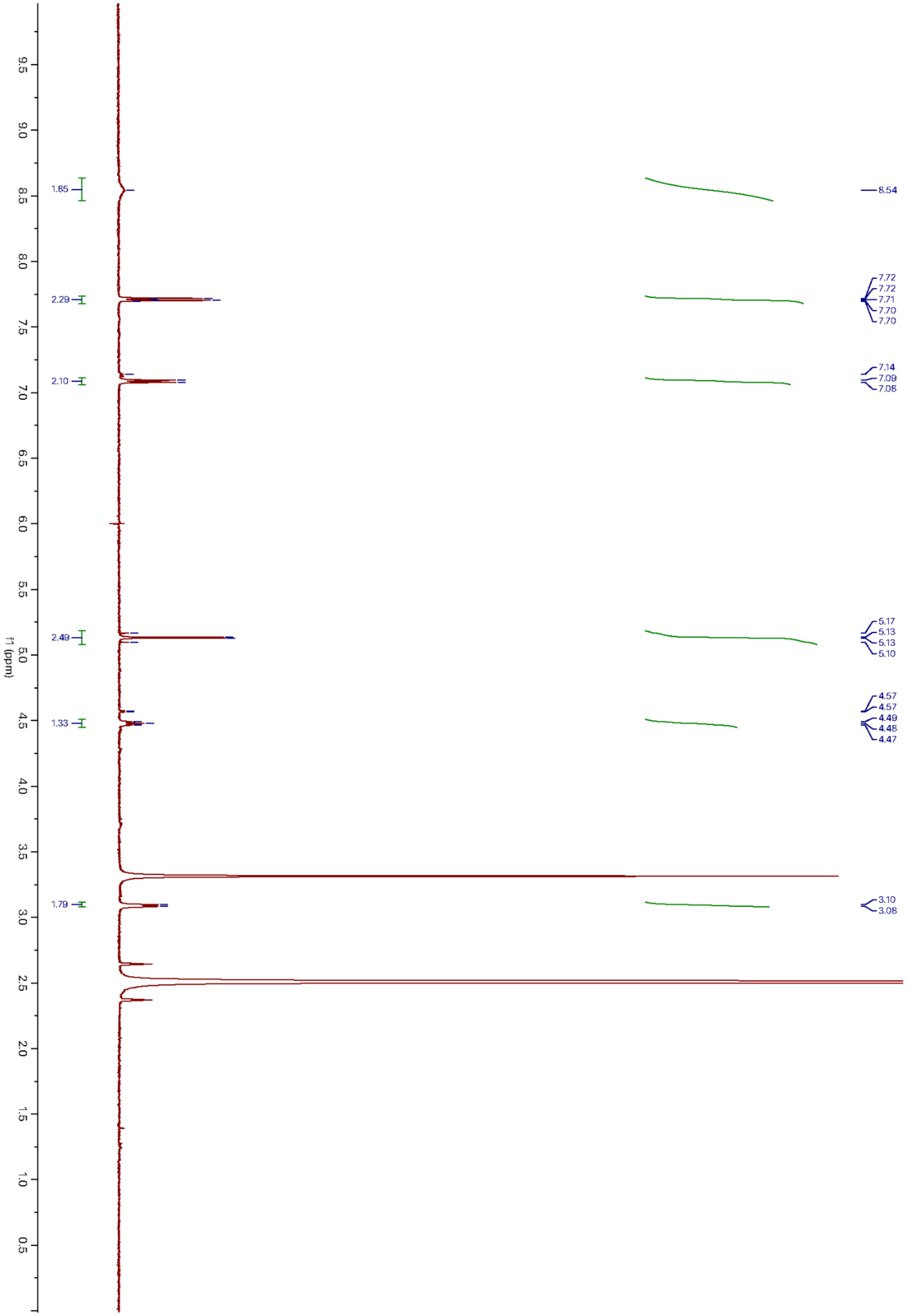

Compound S6 ($^{13}C\{^1H\}$, 126 MHz, DMSO, standard view)

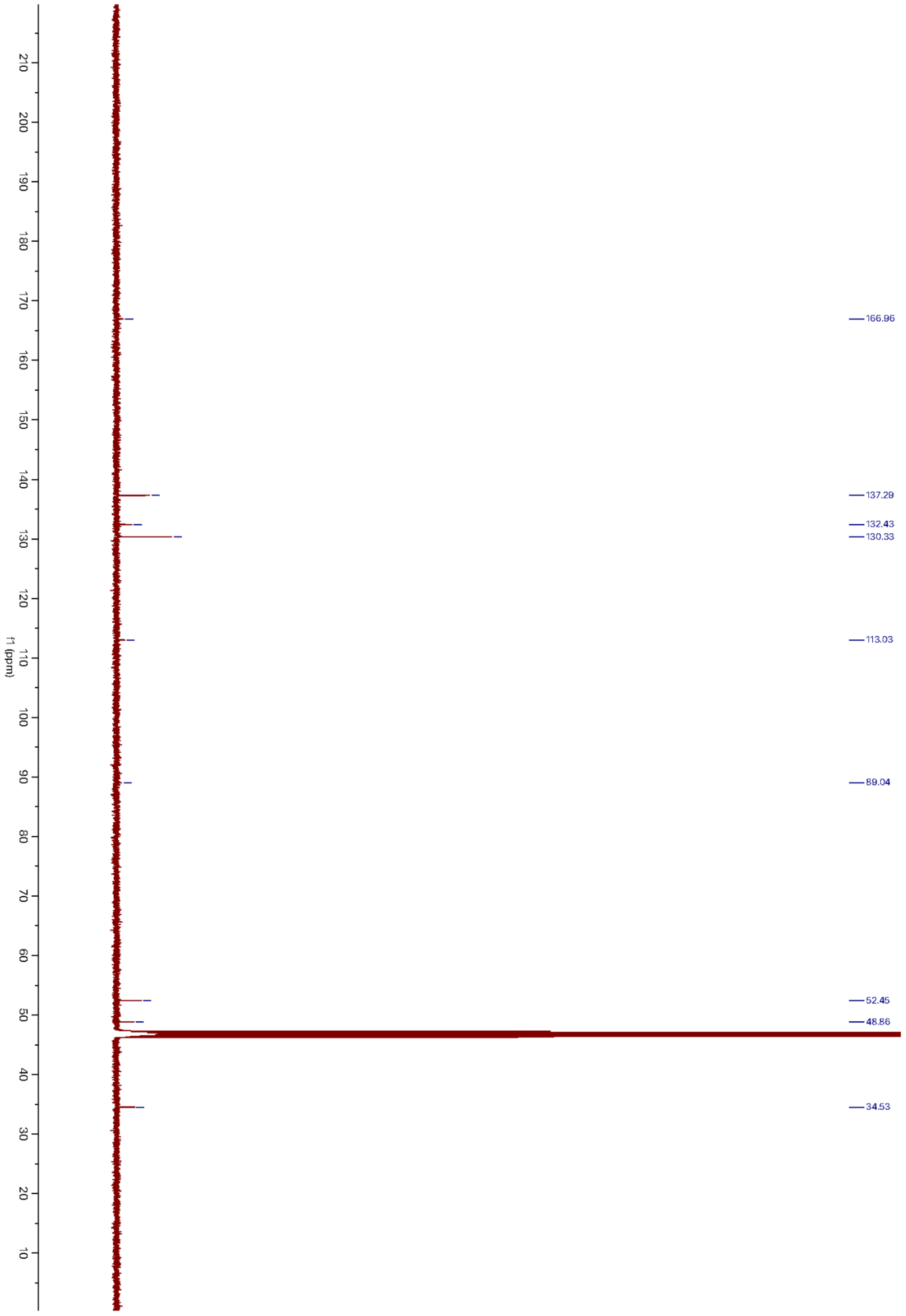

Biochemistry Methods

Transcription of tRNA^{fMet}(-A), tRNA^{Phe}(-A), tRNA^{Pyl}, and tRNA^{Pyl}(-A)

DNA templates for *E. coli* tRNA^{fMet} (C1G mutation),[6] tRNA^{Phe}, *M. alvus* tRNA^{Pyl}, and eFx (flexizyme) were obtained as Ultramers from Integrated DNA Technologies (IDT) (Table 1). Templates for use in transcription reactions were PCR amplified with the corresponding forward and reverse primers (Table 1). Reverse primers included a 2′-O-methyl modification to reduce heterogeneity on the 3′-end of the sequence during transcription.[7] PCR reactions were performed with 5 ng of template, 0.5 µM forward primer, 0.5 µM reverse primer and Q5 High-Fidelity 2X Master Mix (New England BioLabs). *In vitro* transcription of tRNAs was carried out in transcription buffer (50 mM Tris pH 7.5, 2 mM spermidine, 0.1% triton X100), 12 mM $MgCl_2$, 8 mM DTT, 2 mM each nucleotide (NEB), 1 U/µL of murine RNase inhibitor (NEB), 0.5 U/µL *E. coli* inorganic pyrophosphatase (NEB), 2 U/µL of T7 RNA polymerase (NEB) and 15% v/v of the PCR reaction containing the amplified DNA template. The reaction was incubated at 37 ºC for 4 hours after which the products were precipitated with EtOH and purified on a 10% polyacrylamide, 7 M urea, 0.5X TBE (50 mM Tris, 50 mM boric acid, 1 mM EDTA) gel. Bands corresponding to tRNA were excised from the gel and the RNA was eluted for 2 hours in 300 mM KCl after which the gel pieces were removed and the tRNA was precipitated with EtOH. The pellets were resuspended in water and stored in a -80 ºC freezer prior to use.

HPLC purification of 3′-thio-3′-deoxyadenosine triphosphate

Lyophilized 3′-thio-3′-deoxyadenosine-triphosphate (3′-thio-ATP) crude was resuspended in 100mM Triethyl Ammonium Acetate (TEAA) in water (pH 6.5) to a concentration of 10 µM. 400 pmol of 3′-thio-ATP in 40 µL was injected onto an Agilent 1290 Infinity II UHPLC (G7120AR, Agilent) equipped with a XBridge™ Premier Oligo BEH C18 Column (130Å 2.5 µm 2.1 x 100 mm, 25˚C, Waters) with mobile phases A (water with 100mM TEAA, pH adjusted to 6.5) and B (50% acetonitrile/50% mobile phase A). A flow rate of 0.3 mL/min was used throughout. After an initial hold at 0% B for two minutes, a linear gradient was used from 0-60% B over 18 minutes, and followed by a ramp to 100% B for 4 minutes. The flow was held at 100% B for 1 minutes, then brought back to 0% B over 1 minute and finally reequilibrated in 0% B for 19 minutes. It was found that this extended reequilibration period was necessary for reproducibility in separation from precursors or any contaminating ATP. Elution of 3′-thio-ATP was monitored by UV absorbance at 260 nm using a UV detector (1290 Infinity II DA detector with 60 mm flow cell ((G7117BR), Agilent). Fractions were collected (G1364C, Agilent) corresponding to the 3′-thio-ATP, lyophilized to remove solvent and volatile excess TEAA, and stored at -80˚C as a dry solid until further use.

Addition of 3′-thio-3′-deoxyadenosine triphosphate to the 3′-end of tRNAs (-A)

tRNA^{fMet} (-A), tRNA^{Phe} (-A), and tRNA^{Pyl} (-A) were modified with a 3′-thio-3′-deoxyadenosine-triphosphate (3′-thio-ATP) using an *E. coli* CCA-adding enzyme (purified using previously published protocols)[8] to produce 3′-thio-tRNA^{fMet}, 3′-thio-tRNA^{Phe}, and 3′-thio-tRNA^{Pyl} respectively. The tRNA (4-8 nmol) was refolded in 10 mM $MgCl_2$ and then added to a reaction containing 100 mM glycine pH 9.0, 10 mM $MgCl_2$, 100 µM 3′-thio-ATP, 1 mM TCEP, 0.1 U/µL of *E. coli* inorganic pyrophosphatase, 1 U/µL of murine RNase inhibitor, and an equimolar concentration of CCA-adding enzyme to the tRNA. Reactions were incubated at 37 ºC for 3 hours then the tRNA was purified using an RNA Clean and Concentrator Kit (Zymo Research).

Analysis of tRNA reactions by liquid-chromatography mass-spectrometry (LCMS)

Reactions were analyzed by liquid-chromatography mass-spectrometry (LCMS) to confirm identity.[9] Samples were resolved on an ACQUITY UPLC BEH C18 Column (130 Å, 1.7 µm, 2.1 mm x 50 mm, 60 ºC) using ACQUITY UPLC I-Class PLUS. The mobile phases were Solvent A (8 mM triethylamine, 80 mM hexafluoroisopropanol, and 5 µM EDTA in MilliQ water) and Solvent B (4 mM triethylamine, 40 mM hexafluoroisopropanol, and 5 µM EDTA in 50% MilliQ water and 50% methanol). The products were resolved using the following method with a flow rate of 0.3 mL/min: beginning with mobile phase B at 22% then increasing linearly to 40% over 10 minutes, then from 40% to 60% B over one minute and held for one minute, then mobile phase B was decreased to 22% over 0.1, and finally held at 22% B for 2.9 minutes. The mass of the RNA was obtained using a LC-HRMS with a Waters Xevo G2-XS ToF in negative ion mode with the following parameters: capillary voltage: 2000 V, sampling cone: 40, source offset: 40, source temperature: 140 °C, desolvation temperature: 20 °C, cone gas flow: 10 L/h, desolvation gas flow: 800 L/h, 1 spectrum/s. The masses were analyzed and deconvoluted using the MaxEnt software (Waters Corporation).

Enzymatic acylation of 3′-thio-tRNA^{Pyl}, tRNA^{Pyl}, and tRNA$^{Pyl(Ser)}$ with Boc-lysine

Boc-lysine (ThermoFisher Scientific) was charged enzymatically onto 3′-thio-tRNA^{Pyl}, tRNA^{Pyl}, and tRNA$^{Pyl(Ser)}$ using *M. alvus* pyrrolysyl-tRNA synthetase PylRS (obtained from the Schepartz Lab) to generate 3′-BocLys-thioester-tRNA^{Pyl}, 3′-BocLys-tRNA^{Pyl}, and 3′-BocLys-tRNA$^{Pyl(Ser)}$ containing either a thioester or ester linkage. A reaction containing tRNA (4-8 nmol), 50 mM HEPES pH 7.5, 20 mM $MgCl_2$, 10 mM KCl, 2 mM TCEP, 10 mM ATP, 1 U/µL RNase inhibitor, 5 mM Boc-lysine, and 1 µM PylRS was incubated at 37 ºC for 30 minutes. The reaction was purified using an RNA Clean and Concentrator Kit. Samples were analyzed using LCMS as described above. Percent yields were calculated by extracting the major ion corresponding to each tRNA species and integrating the subsequent extracted ion chromatogram.

Enzymatic acylation of tRNA$^{Pyl(Ser)}$ with (*R*)-β^2-OH-*N*$_ε$-BocLys

(*R*)-β^2-OH-*N*$_ε$-BocLys ((*R*)-β^2-OH) (Enamine) was enzymatically charged onto tRNA$^{Pyl(Ser)}$. A reaction containing 25 µM tRNA, 100 mM HEPES pH 7.5, 10 mM $MgCl_2$, 4 mM DTT, 10 mM ATP, 0.004 U/µL iPPase (New England Biolabs), 5 mM (*R*)-β^2-OH, and 5 µM PylRS was incubated at 37 ºC for 1 hour. The reaction was purified using an RNA Clean and Concentrator Kit. Samples were analyzed using LCMS as described above. Percent yields were calculated by extracting the major ion corresponding to each tRNA species and integrating the subsequent extracted ion chromatogram.

Enzymatic acylation of 3′-thio-tRNA^{fMet} and tRNA^{fMet} with homopropargylglycine

Homopropargylglycine (Hpg) (Millipore Sigma) was enzymatically added to 3′-thio-tRNA^{fMet} and tRNA^{fMet} using *E. coli* methionyl-tRNA synthetase MetRS (purified as previously reported) to generate 3′-Hpg-thioester-tRNA^{fMet} and 3′-Hpg-tRNA^{fMet} containing either a thioester or ester linkage. A reaction containing tRNA (4-8 nmol), 50 mM HEPES pH 7.5, 20 mM $MgCl_2$, 10 mM KCl, 2 mM TCEP, 10 mM ATP, 1 U/µL RNase inhibitor, 5 mM Hpg, and 1 µM MetRS was incubated at 37 ºC for 1 hour. The reaction was purified using an RNA Clean and Concentrator Kit. Samples were analyzed using LCMS as described above. Percent yields were calculated by extracting the major ion corresponding to each tRNA species and integrating the subsequent extracted ion chromatogram.

Enzymatic acylation of 3′-thio-tRNA^{Phe} and tRNA^{Phe} with phenylalanine

Phenylalanine (Phe) (Millipore Sigma) was enzymatically added to 3′-thio-tRNA^{Phe} and tRNA^{Phe} using *E. coli* phenylalanyl-tRNA synthetase PheRS (purified as previously reported) to generate 3′-Phe-thioester-tRNA^{Phe} and 3′-Hpg-tRNA^{Phe} containing either a thioester or ester linkage. A reaction containing tRNA (4-8 nmol), 50 mM HEPES pH 7.5, 20 mM $MgCl_2$, 10 mM KCl, 2 mM TCEP, 10 mM ATP, 1 U/µL RNase inhibitor, 5 mM Phe, and 1 µM PheRS was incubated at 37 ºC for 15 mins. The reaction was purified using an RNA Clean and Concentrator Kit. Samples were analyzed using LCMS as described above. Percent yields were calculated by extracting the major ion corresponding to each tRNA species and integrating the subsequent extracted ion chromatogram.

Flexizyme charging of 3′-thio-tRNA^{Pyl} and tRNA^{Pyl} with phenylalanine

A flexizyme (eFx) was used to promote the reaction between 3′-thio-tRNA^{Pyl} or tRNA^{Pyl} and phenylalanine cyanomethyl ester (Phe-CME). The tRNA (25 µM) was added to a reaction containing 25 µM eFx, 50 mM HEPES pH 7.5, 400 mM $MgCl_2$, and 4 mM TCEP and co-folded. Following refolding, 5 mM Phe-CME was added and reacted at 8 ºC for 4 hours. The reaction was purified using an RNA Clean and Concentrator Kit and samples were analyzed using LCMS as described above. Percent yields were calculated by extracting the major ion corresponding to each tRNA species and integrating the subsequent extracted ion chromatogram.

Flexizyme charging of 3′-thio-tRNA^{Pyl} and tRNA^{Pyl} with leucine

A flexizyme (aFx) (obtained from the Schepartz Lab) was used to promote the reaction between 3′-thio-tRNA^{Pyl} or tRNA^{Pyl} and leucine activated with an amino derivatized benzyl-thioester (Leu-ABT) (obtained from the Schepartz Lab). The tRNA (25 µM) was added to a reaction containing 25 µM aFx, 50 mM HEPES pH 7.5, 100 mM $MgCl_2$, and 4 mM TCEP and co-folded. Following refolding, 5 mM Leu-ABT was added and reacted at 8 ºC for 6 hours. The reaction was purified using an RNA Clean and Concentrator Kit and samples were analyzed using LCMS as described above. Percent yields were calculated by extracting the major ion corresponding to each tRNA species and integrating the subsequent extracted ion chromatogram.

Flexizyme charging of 3′-thio-tRNA^{Pyl} and tRNA^{Pyl} with iodo-phenylalanine

A flexizyme (eFx) (obtained from the Schepartz Lab) was used to promote the reaction between 3′-thio-tRNA^{Pyl} or tRNA^{Pyl} and iodo-phenylalanine (PheI) activated with PheI cyanomethyl ester (PheI-CME) (**S6**). The tRNA (25 µM) was added to a reaction containing 25 µM eFx, 50 mM HEPES pH 7.5, 400 mM $MgCl_2$, and 4 mM TCEP and co-folded. Following refolding,

5 mM PheI-CME was added and reacted at 8 ºC for 4 hours. The reaction was purified using an RNA Clean and Concentrator Kit and samples were analyzed using LCMS as described above. Percent yields were calculated by extracting the major ion corresponding to each tRNA species and integrating the subsequent extracted ion chromatogram.

Thioester exchange to charge 3′-thio-tRNA^{Pyl} with leucine

In the absence of aFx, 3′-thio-tRNA^{Pyl} was charged with leucine from the activated Leu-ABT through thioester exchange. The tRNA (25 µM) was added to a reaction containing 50 mM HEPES pH 7.5, 100 mM $MgCl_2$, 4 mM TCEP and 5 mM Leu-ABT and reacted at 8 ºC for 6 hours. The reaction was purified using an RNA Clean and Concentrator Kit and samples were analyzed using LCMS as described above. Percent yields were calculated by extracting the major ion corresponding to each tRNA species and integrating the subsequent extracted ion chromatogram.

In vitro translation of peptides containing a Boc-lysine originating from 3′-thio-tRNA^{Pyl} or tRNA^{Pyl}

Peptide-1 (Table 2) was generated using *in vitro* transcription/translation (IVTT) from a template containing a T7 promoter, a ribosome binding site, the coding sequence for a short peptide with a FLAG-tag, and an amber stop codon. The stop codon was decoded by either 3′-BocLys-thioester-tRNA^{Pyl} or BocLys-tRNA^{Pyl}. DNA templates were obtained as Ultramers (Table 1) from IDT and PCR amplified (5 ng of template, 0.5 µM forward primer, 0.5 µM reverse primer and Q5 High-Fidelity 2X Master Mix). Templates were purified using a DNA Clean and Concentrator Kit (Zymo Research). IVTT reactions were performed using a ΔRF Purexpress kit (NEB) where release factor 1 was omitted from the reactions. All Purexpress reagents (5 µL Reagent A, 3.25 µL Reagent B ΔRF123, 0.25 µL RF2, and 0.25 µL RF3) were added on ice, then 14-25 µM acylated tRNA (either 3′-BocLys-thioester-tRNA^{Pyl} or BocLys-tRNA^{Pyl}) was added (adjusted for acylation efficiency as determined by LCMS). Finally, the reaction was initiated by addition of 100 ng of DNA template. In control reactions, either no tRNA or 14-25 µM unacylated 3′-thio-tRNA^{Pyl} were added. Reactions were incubated at 37 ºC for 4 hours.

In vitro translation of peptides containing a PheI originating from 3′-thio-tRNA^{Pyl} or tRNA^{Pyl}

Peptide-1 (Table 2) was generated using *in vitro* transcription/translation (IVTT) from a template containing a T7 promoter, a ribosome binding site, the coding sequence for a short peptide with a FLAG-tag, and an amber stop codon. The stop codon was decoded by either 3′-PheI-thioester-tRNA^{Pyl} or PheI-tRNA^{Pyl}. DNA templates were obtained as Ultramers (Table 1) from IDT and PCR amplified (5 ng of template, 0.5 µM forward primer, 0.5 µM reverse primer and Q5 High-Fidelity 2X Master Mix). Templates were purified using a DNA Clean and Concentrator Kit (Zymo Research). IVTT reactions were performed using a ΔRF Purexpress kit (NEB) where release factor 1 was omitted from the reactions. All Purexpress reagents (5 µL Reagent A, 3.25 µL Reagent B ΔRF123, 0.25 µL RF2, and 0.25 µL RF3) were added on ice, then 14-25 µM acylated tRNA (either 3′-PheI-thioester-tRNA^{Pyl} or PheI-tRNA^{Pyl}) was added (adjusted for acylation efficiency as determined by LCMS). Finally, the reaction was initiated by addition of 100 ng of DNA template. In control reactions, either no tRNA or 14-25 µM unacylated 3′-thio-tRNA^{Pyl} were added. Reactions were incubated at 37 ºC for 4 hours.

In vitro translation of peptides containing two consecutive BocLys monomers originating from 3′-thio-tRNA^{Pyl} and tRNA$^{Pyl(Ser)}$ or tRNA^{Pyl} and tRNA$^{Pyl(Ser)}$

Peptide-2 (Table 2) was generated using *in vitro* transcription/translation (IVTT) from a template containing a T7 promoter, a ribosome binding site, the coding sequence for a short peptide with a FLAG-tag, a recoded amber stop codon, and a recoded serine codon. The stop codon was decoded by either 3′-BocLys-thioester-tRNA^{Pyl} or BocLys-tRNA^{Pyl}. The serine codon was decoded by BocLys-tRNA$^{Pyl(Ser)}$. DNA templates were obtained as Ultramers (Table 1) from IDT and PCR amplified (5 ng of template, 0.5 µM forward primer, 0.5 µM reverse primer and Q5 High-Fidelity 2X Master Mix). Templates were purified using a DNA Clean and Concentrator Kit (Zymo Research). IVTT reactions were performed using a ΔRF Purexpress kit (NEB) and Δaa, ΔtRNA Purexpress kit (NEB) where release factor 1 was omitted from the reactions. All Purexpress reagents (2.5 µL Reagent A Δaa, ΔtRNA, 3.25 µL Reagent B ΔRF123, 1.75 µL total tRNA, 0.25 µL RF2, and 0.25 µL RF3) were added on ice, 2 mM each amino acid (Met, Ala, Tyr, Lys, Asp) was added, then 14-25 µM of each acylated tRNA (either 3′-BocLys-thioester-tRNA^{Pyl} or BocLys-tRNA^{Pyl}) was added (adjusted for acylation efficiency as determined by LCMS). Finally, the reaction was initiated by addition of 100 ng of DNA template. In control reactions, either no tRNA or 14-25 µM unacylated 3′-thio-tRNA^{Pyl} were added. Reactions were incubated at 37 ºC for 4 hours.

In vitro translation of peptides containing a BocLys followed by a β-hydroxy-BocLys monomer originating from 3′-thio-tRNA^{Pyl} and tRNA$^{Pyl(Ser)}$ or tRNA^{Pyl} and tRNA$^{Pyl(Ser)}$

Peptide-2 (Table 2) was generated using *in vitro* transcription/translation (IVTT) from a template containing a T7 promoter, a ribosome binding site, the coding sequence for a short peptide with a FLAG-tag, a recoded amber stop codon, and a recoded serine codon. The stop codon was decoded by either 3′-BocLys-thioester-tRNA^{Pyl} or BocLys-tRNA^{Pyl}. The serine

codon was decoded by (*R*)-β^2-OH-$tRNA^{Pyl(Ser)}$. DNA templates were obtained as Ultramers (Table 1) from IDT and PCR amplified (5 ng of template, 0.5 µM forward primer, 0.5 µM reverse primer and Q5 High-Fidelity 2X Master Mix). Templates were purified using a DNA Clean and Concentrator Kit (Zymo Research). IVTT reactions were performed using a ΔRF Purexpress kit (NEB) and Δaa, ΔtRNA Purexpress kit (NEB) where release factor 1 was omitted from the reactions. All Purexpress reagents (2.5 µL Reagent A Δaa, ΔtRNA, 3.25 µL Reagent B ΔRF123, 1.75 µL total tRNA, 0.25 µL RF2, and 0.25 µL RF3) were added on ice, 2 mM each amino acid (Met, Ala, Tyr, Lys, Asp) was added, then 14-25 µM of each acylated tRNA (either 3′-BocLys-thioester-$tRNA^{Pyl}$ or BocLys-$tRNA^{Pyl}$ and (*R*)-β^2-OH-$tRNA^{Pyl(Ser)}$) was added (adjusted for acylation efficiency as determined by LCMS). Finally, the reaction was initiated by addition of 100 ng of DNA template. In control reactions, either no tRNA or 14-25 µM unacylated 3′-thio-$tRNA^{Pyl}$ were added. Reactions were incubated at 37 ºC for 4 hours.

Purification and analysis of FLAG-tagged peptides

Pierce Anti-DYKDDDDK Magnetic Agarose beads (ThermoFisher Scientific) or Anti-FLAG M2 Magnetic Beads (Millipore Sigma) were used to purify IVTT generated, FLAG-tagged peptides for downstream analysis. Anti-FLAG beads (12.5 µL) were washed with 100 µL wash buffer (50 mM Tris HCl pH 7.5, 150 mM NaCl) and the supernatant was removed from the beads. IVTT reactions were applied to the anti-FLAG beads and incubated at room temperature for 30 minutes. The supernatant was then removed, and the beads were washed 2X with 100 µL of wash buffer, and once with 100 µL of RNase free water. Elution buffer (12.5 µL) (100 mM glycine pH 2.8) was added to the beads and incubated at room temperature for 10 minutes. The supernatant was removed and analyzed by LCMS. Samples were resolved on an an Agilent 1290 Infinity II HPLC using an Eclipse XDB C18 Column (1.8 µm, 2.1mm x 50 mm, 25 ºC) (Agilent) with mobile phases A (water with 0.1 % [v/v] formic acid) and B (acetonitrile with 0.1 % [v/v] formic acid). The method began with a linear gradient from 0% B to 1% B for one minute, then a linear gradient was used from 1-91% B over five minutes at a flow rate of 0.5 mL/min, then subsequently washed with 91% B for 0.6 minutes, then a linear gradient decreased B from 91% to 1% for 1.1 minutes, finally the column was equilibrated 1% B for 1.3 minutes. The mass spectra of each sample was obtained on an Agilent 6530B QTOF AJS-ESI with the following settings: gas temp. 300 ºC, nebulizer 35 psi, drying gas 12 L/min, sheath gas temp. 350 ºC, sheath gas flow 12 L/min, fragmentor voltage 175 V, skimmer voltage 65 V, Vcap 3500 V, nozzle voltage 1000 V. The masses were analyzed using the MassHunter software (Agilent) and percent yields were calculated by extracting the major ion corresponding to each peptide species (± 100 ppm) and integrating the subsequent extracted ion chromatogram.

Supplementary Figures

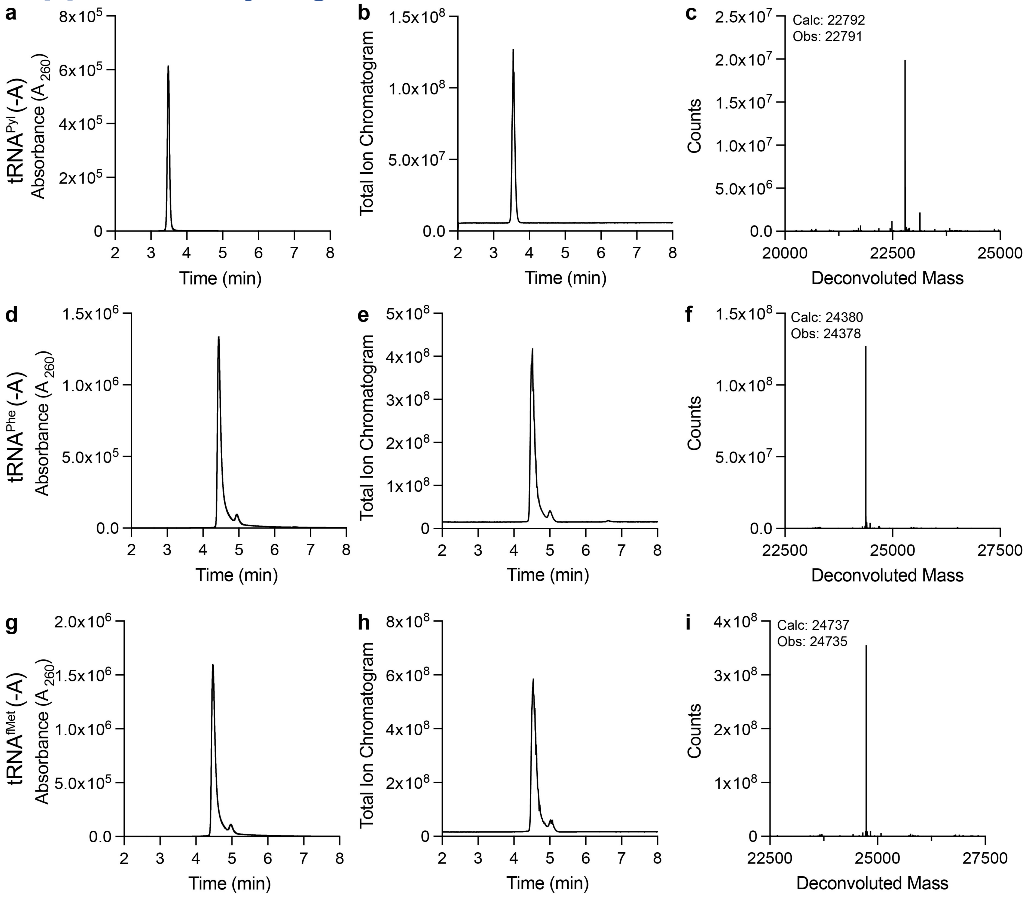

Figure S6. Intact tRNA LC-MS of truncated-tRNAs (-A).

(a) Liquid chromatogram (LC) of $tRNA^{Pyl}$(-A). (b) Total ion chromatogram (TIC) of $tRNA^{Pyl}$(-A). (c) Mass spectra of $tRNA^{Pyl}$(-A) deconvoluted from m/z data. (d) LC of $tRNA^{Phe}$(-A). (e) TIC of $tRNA^{Phe}$(-A). (f) Mass spectra of $tRNA^{Phe}$(-A) deconvoluted from m/z data. (g) LC of $tRNA^{fMet}$(-A). (h) TIC of $tRNA^{fMet}$(-A). (i) Mass spectra of $tRNA^{fMet}$(-A) deconvoluted from m/z data.

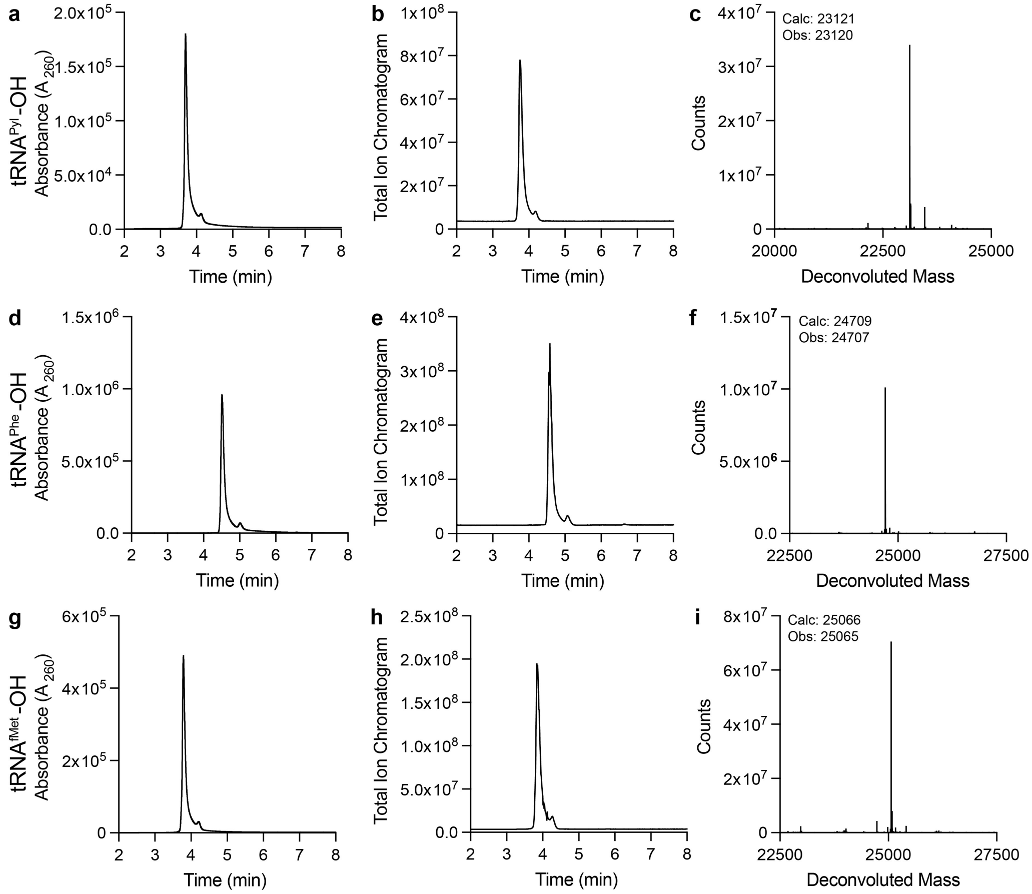

Figure S7. Intact tRNA LC-MS of full length tRNAs.

(a) Liquid chromatogram (LC) of tRNA^{Pyl}-OH. (b) Total ion chromatogram (TIC) of tRNA^{Pyl}-OH. (c) Mass spectra of tRNA^{Pyl}-OH deconvoluted from m/z data. (d) LC of tRNA^{Phe}-OH. (e) TIC of tRNA^{Phe}-OH. (f) Mass spectra of tRNA^{Phe}-OH deconvoluted from m/z data. (g) LC of tRNA^{fMet}-OH. (h) TIC of tRNA^{fMet}-OH. (i) Mass spectra of tRNA^{fMet}-OH deconvoluted from m/z data.

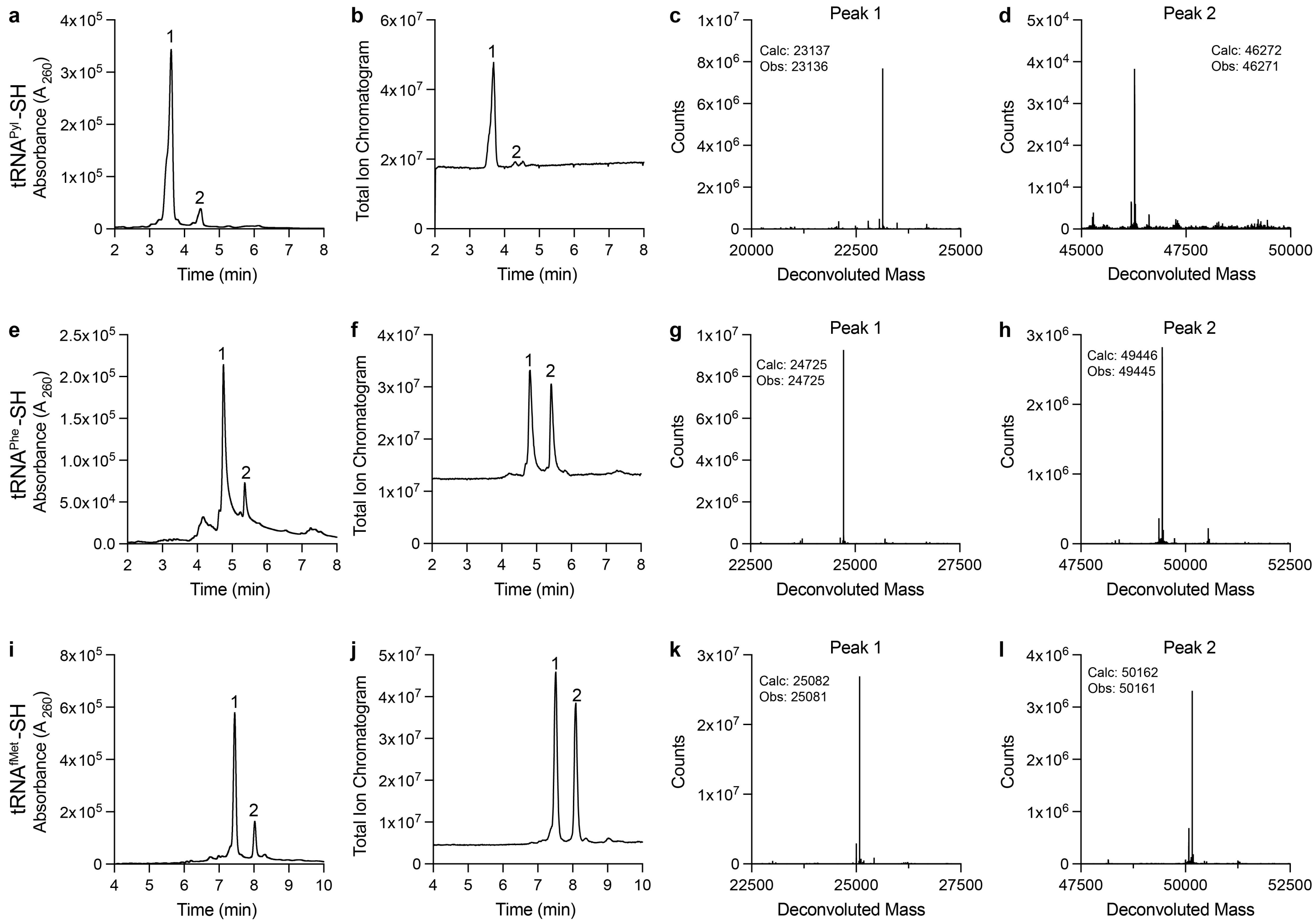

Figure S8. Intact tRNA LC-MS of 3′-thio-tRNAs.

LC-MS was used to determine that the *E. coli* CCA-adding enzyme adds 3′-thio-3′-deoxadenosine to truncated-tRNAs (-A) to produce 3′-thio-tRNAs in quantitative yield. (a) Liquid chromatogram (LC) of tRNA^{Pyl}-SH. Peak 1 corresponds to tRNA^{Pyl}-SH. Peak 2 corresponds to tRNA^{Pyl}-S-S-tRNA^{Pyl}. (b) Total ion chromatogram (TIC) of tRNA^{Pyl}-SH. (c) Mass spectra of tRNA^{Pyl}-SH peak 1 deconvoluted from m/z data. (d) Mass spectra of tRNA^{Pyl}-SH peak 2 deconvoluted from m/z data. (e) Liquid chromatogram (LC) of tRNA^{Phe}-SH. Peak 1 corresponds to tRNA^{Phe}-SH. Peak 2 corresponds to tRNA^{Phe}-S-S-tRNA^{Phe}. (f) Total ion chromatogram (TIC) of tRNA^{Phe}-SH. (g) Mass spectra of tRNA^{Phe}-SH peak 1 deconvoluted from m/z data. (h) Mass spectra of tRNA^{Pyl}-SH peak 2 deconvoluted from m/z data. (i) Liquid chromatogram (LC) of tRNA^{fMet}-SH. Peak 1 corresponds to tRNA^{fMet}-SH. Peak 2 corresponds to tRNA^{fMet}-S-S-tRNA^{fMet}. (j) Total ion chromatogram (TIC) of tRNA^{fMet}-SH. (k) Mass spectra of tRNA^{fMet}-SH peak 1 deconvoluted from m/z data. (l) Mass spectra of tRNA^{fMet}-SH peak 2 deconvoluted from m/z data. Two additional replicates of each reaction were performed and analyzed by LC-MS. These raw and graphed data are included in the fair data files.

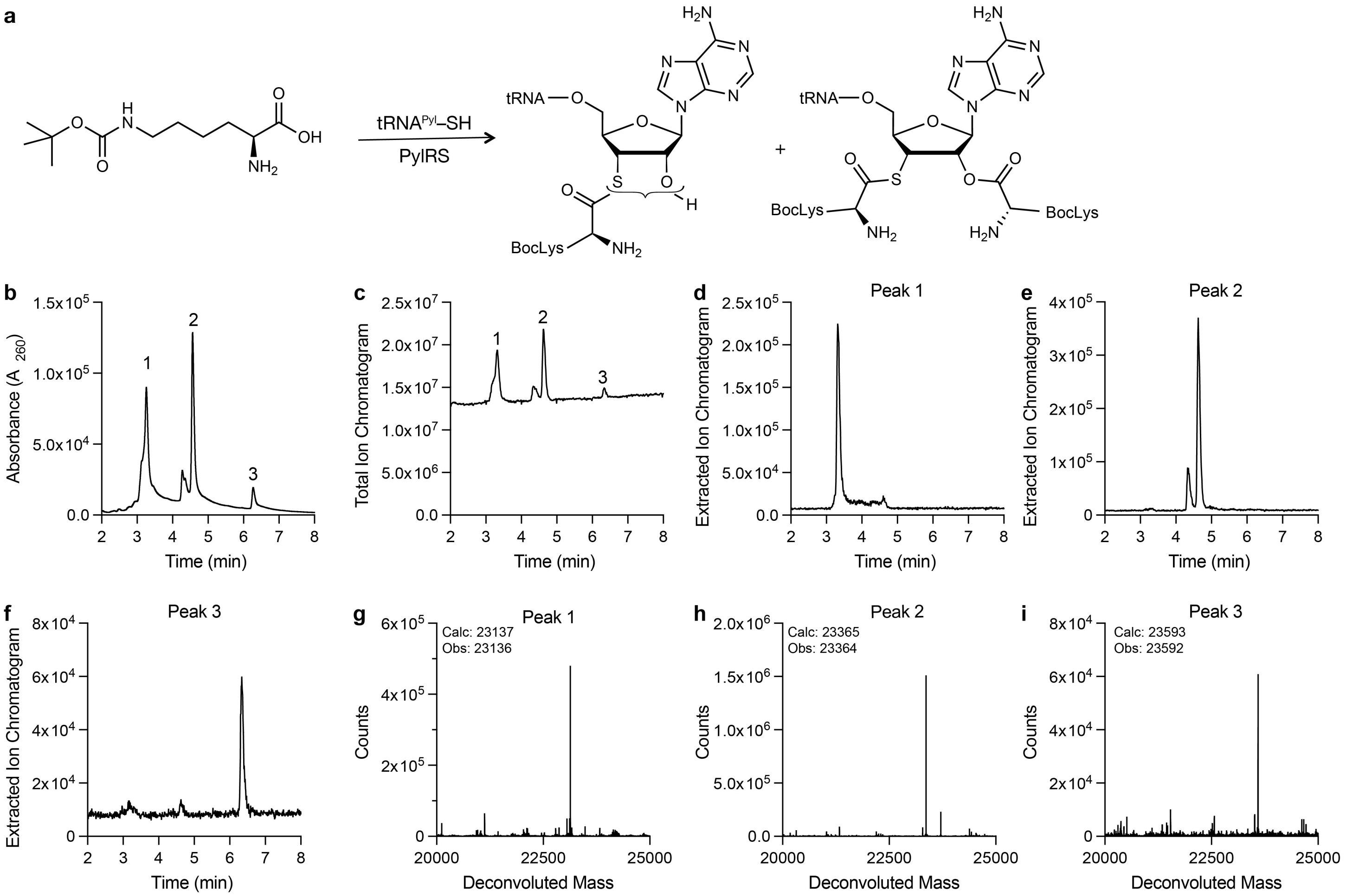

Figure S9. Intact tRNA LC-MS of N_ε-boc-L-lysine (BocLys) added to 3′-thio-tRNA^{Pyl} using PylRS.
LC-MS was used to determine the acylation efficiency of addition of BocLys to 3′-thio-tRNA^{Pyl} (tRNA-SH) using PylRS. (a) Schematic showing either mono- or di-acylation of tRNA-SH with BocLys. (b) Liquid chromatogram (LC) of acylation reaction. Peak 1 is tRNA-SH, peak 2 is tRNA-S-BocLys, and peak 3 is tRNA-S-BocLys(x2). (c) Total ion chromatogram (TIC) of acylation reaction. (d) Extracted ion chromatogram (EIC) of peak 1, tRNA-SH. (e) EIC of peak 2, tRNA-S-BocLys. (f) EIC of peak 3, tRNA-S-BocLys(x2). (g) Mass spectra of peak 1 deconvoluted from m/z data. (h) Mass spectra of peak 2 deconvoluted from m/z data. (i) Mass spectra of peak 3 deconvoluted from m/z data. Two additional replicates of each reaction were performed and analyzed by LC-MS. These raw and graphed data are included in the fair data files.

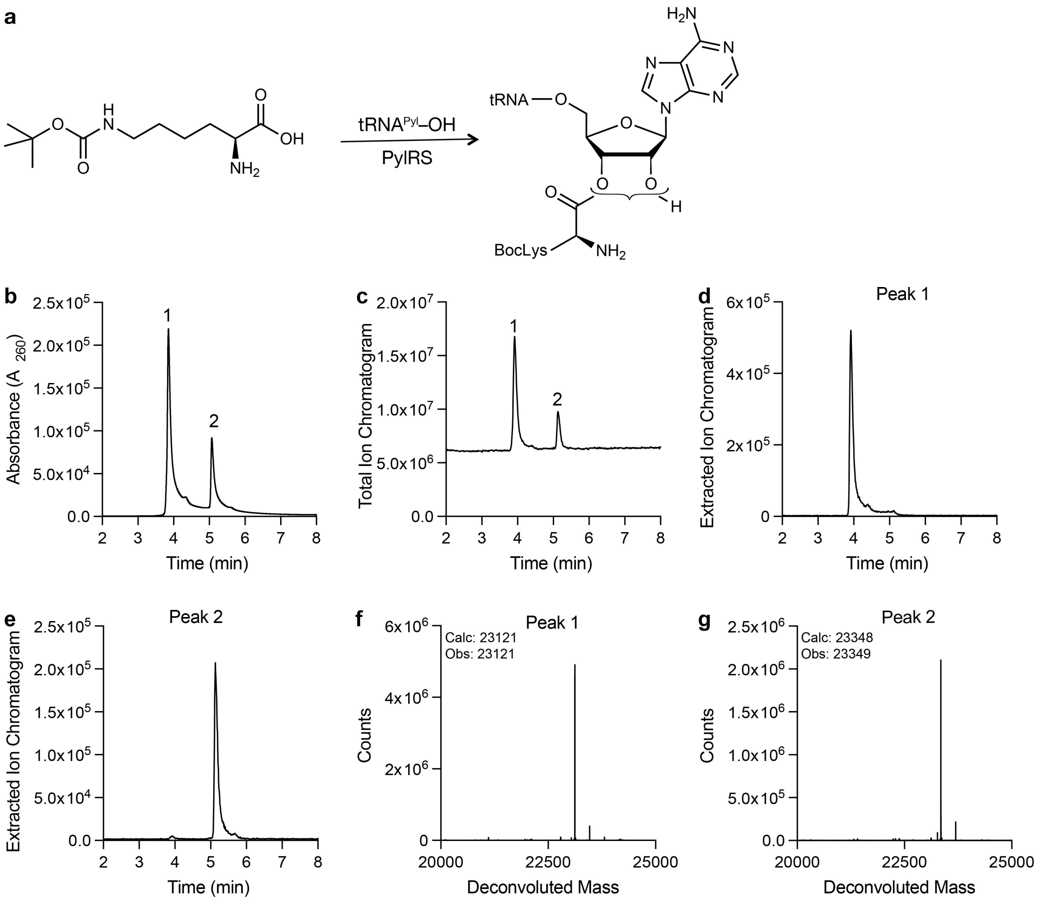

Figure S10. Intact tRNA LC-MS of *N*$_\varepsilon$-boc-L-lysine (BocLys) added to tRNA^{Pyl} using PylRS.
LC-MS was used to determine the acylation efficiency of addition of BocLys to tRNA^{Pyl} (tRNA-OH) using PylRS. (a) Schematic showing mono-acylation of tRNA-OH with BocLys. (b) Liquid chromatogram (LC) of acylation reaction. Peak 1 is tRNA-OH and peak 2 is tRNA-O-BocLys. (c) Total ion chromatogram (TIC) of acylation reaction. (d) Extracted ion chromatogram (EIC) of peak 1, tRNA-OH. (e) EIC of peak 2, tRNA-O-BocLys. (f) Mass spectra of peak 1 deconvoluted from m/z data. (g) Mass spectra of peak 2 deconvoluted from m/z data. Two additional replicates of each reaction were performed and analyzed by LC-MS. These raw and graphed data are included in the fair data files.

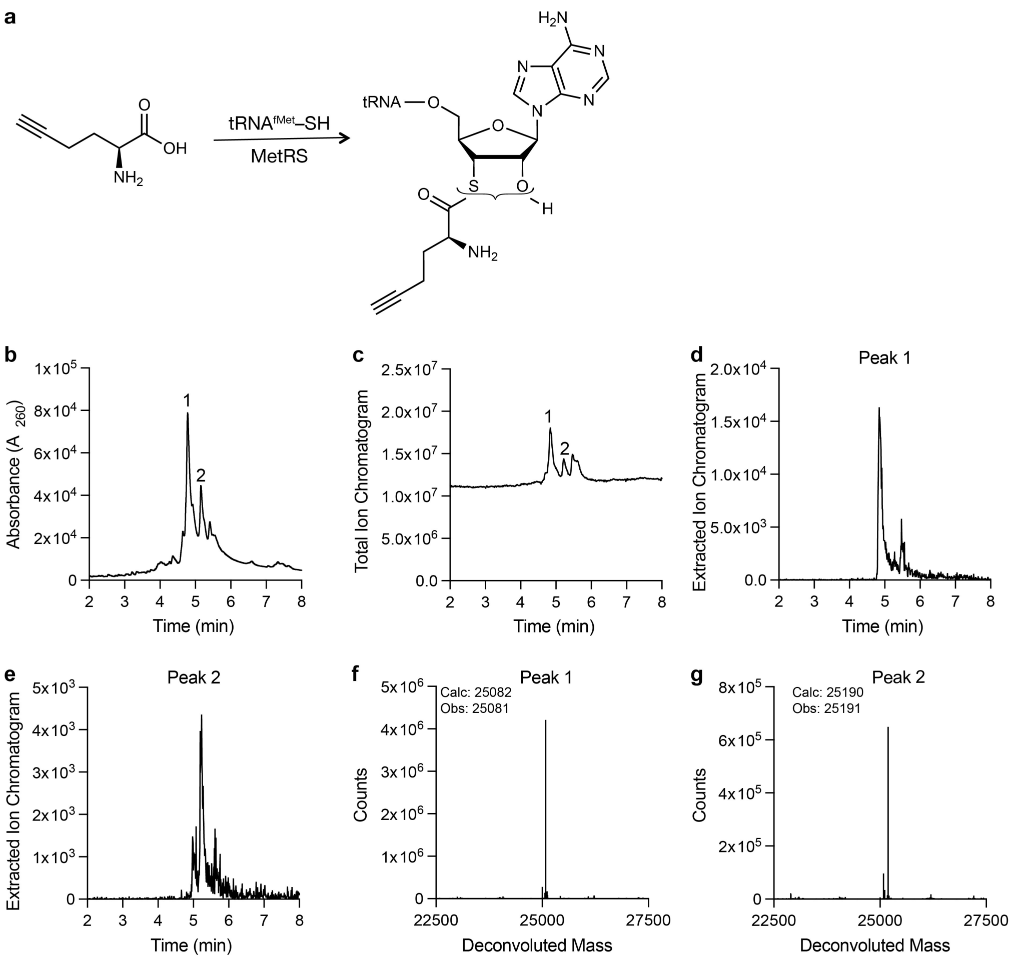

Figure S11. Intact tRNA LC-MS of homopropargylglycine (Hpg) added to 3′-thio-tRNA^{fMet} using MetRS. LC-MS was used to determine the acylation efficiency of addition of Hpg to 3′-thio-tRNA^{fMet} (tRNA-SH) using MetRS. (a) Schematic showing mono-acylation of tRNA-SH with Hpg. (b) Liquid chromatogram (LC) of acylation reaction. Peak 1 is tRNA-SH and peak 2 is tRNA-S-Hpg. (c) Total ion chromatogram (TIC) of acylation reaction. (d) Extracted ion chromatogram (EIC) of peak 1, tRNA-SH. (e) EIC of peak 2, tRNA-S-Hpg. (f) Mass spectra of peak 1 deconvoluted from m/z data. (g) Mass spectra of peak 2 deconvoluted from m/z data. Two additional replicates of each reaction were performed and analyzed by LC-MS. These raw and graphed data are included in the fair data files.

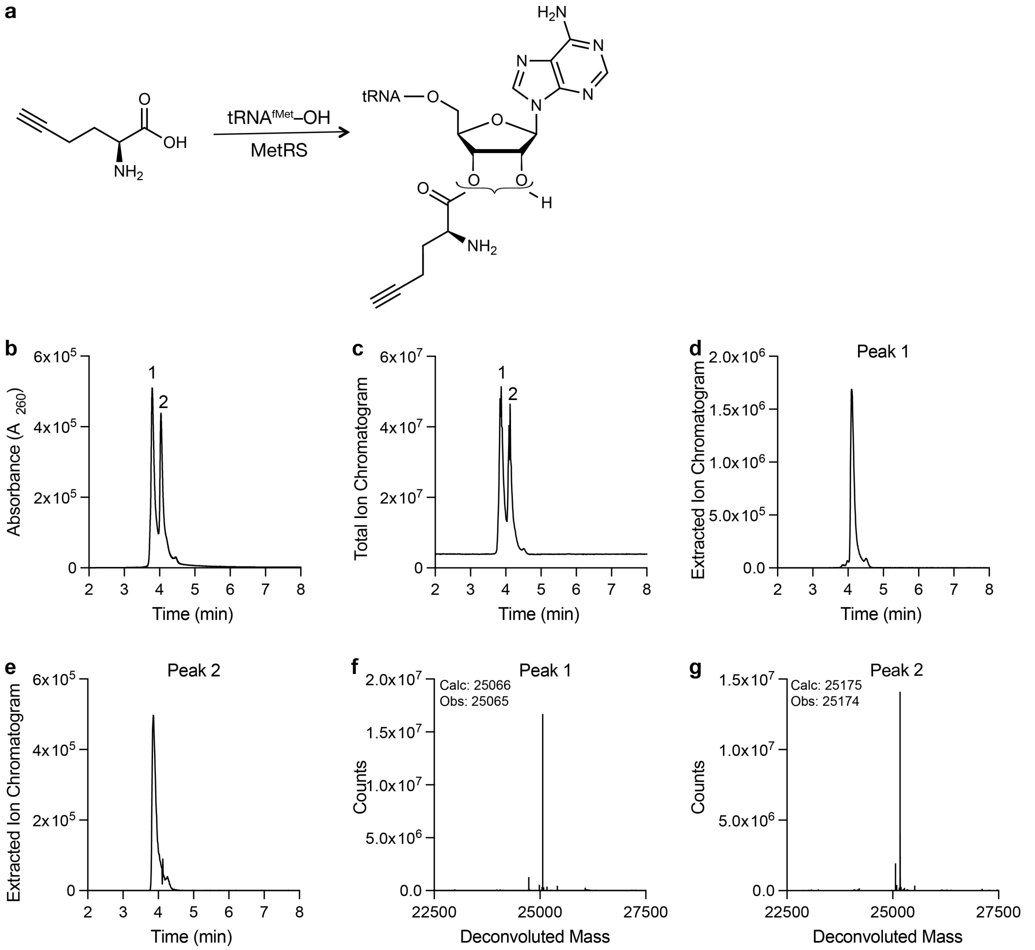

Figure S12. Intact tRNA LC-MS of homopropargylglycine (Hpg) added to tRNA^{fMet} using MetRS.
LC-MS was used to determine the acylation efficiency of addition of Hpg to tRNA^{fMet} (tRNA-OH) using MetRS. (a) Schematic showing mono-acylation of tRNA-OH with Hpg. (b) Liquid chromatogram (LC) of acylation reaction. Peak 1 is tRNA-OH and peak 2 is tRNA-O-Hpg. (c) Total ion chromatogram (TIC) of acylation reaction. (d) Extracted ion chromatogram (EIC) of peak 1, tRNA-OH. (e) EIC of peak 2, tRNA-O-Hpg. (f) Mass spectra of peak 1 deconvoluted from m/z data. (g) Mass spectra of peak 2 deconvoluted from m/z data. Two additional replicates of each reaction were performed and analyzed by LC-MS. These raw and graphed data are included in the fair data files.

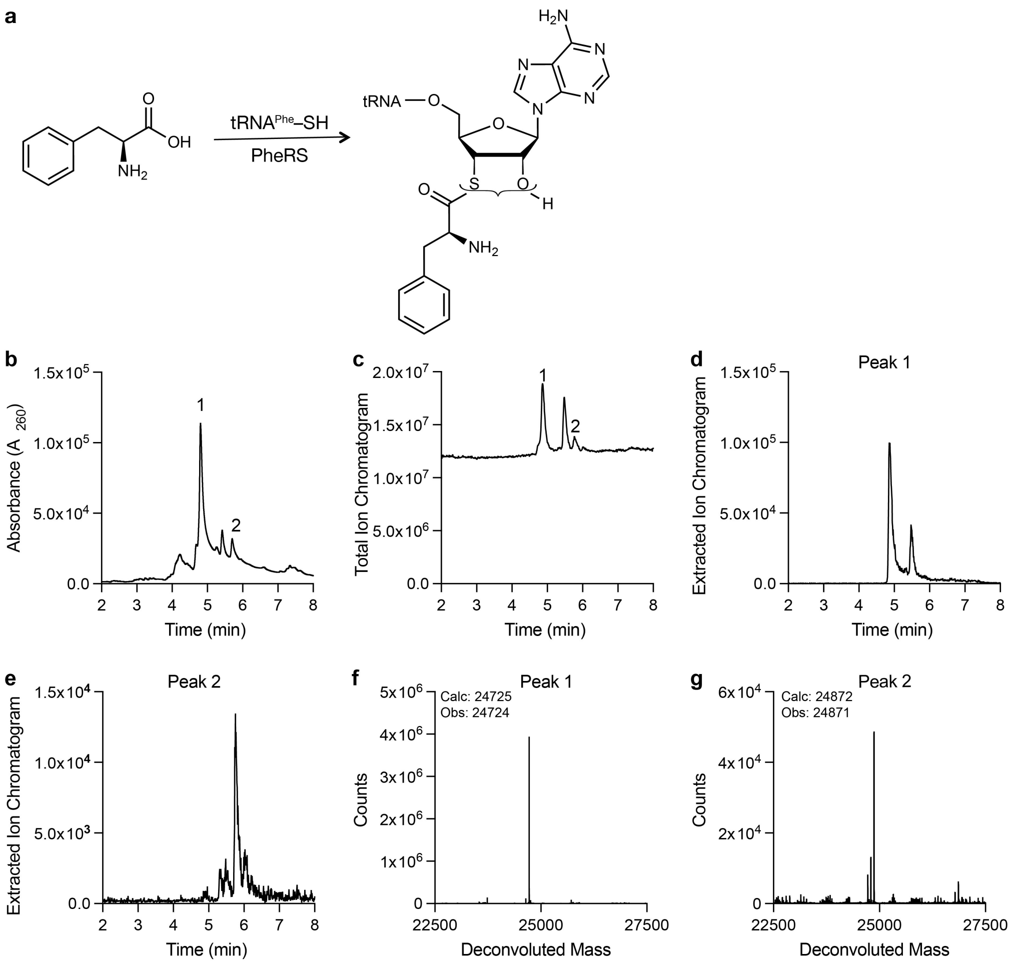

Figure S13. Intact tRNA LC-MS of phenylalanine (Phe) added to 3′-thio-tRNA^{Phe} using PheRS.
LC-MS was used to determine the acylation efficiency of addition of Phe to 3′-thio-tRNA^{Phe} (tRNA-SH) using PheRS. (a) Schematic showing mono-acylation of tRNA-SH with Phe. (b) Liquid chromatogram (LC) of acylation reaction. Peak 1 is tRNA-SH and peak 2 is tRNA-S-Phe. (c) Total ion chromatogram (TIC) of acylation reaction. (d) Extracted ion chromatogram (EIC) of peak 1, tRNA-SH. (e) EIC of peak 2, tRNA-S-Phe. (f) Mass spectra of peak 1 deconvoluted from m/z data. (g) Mass spectra of peak 2 deconvoluted from m/z data. Two additional replicates of each reaction were performed and analyzed by LC-MS. These raw and graphed data are included in the fair data files.

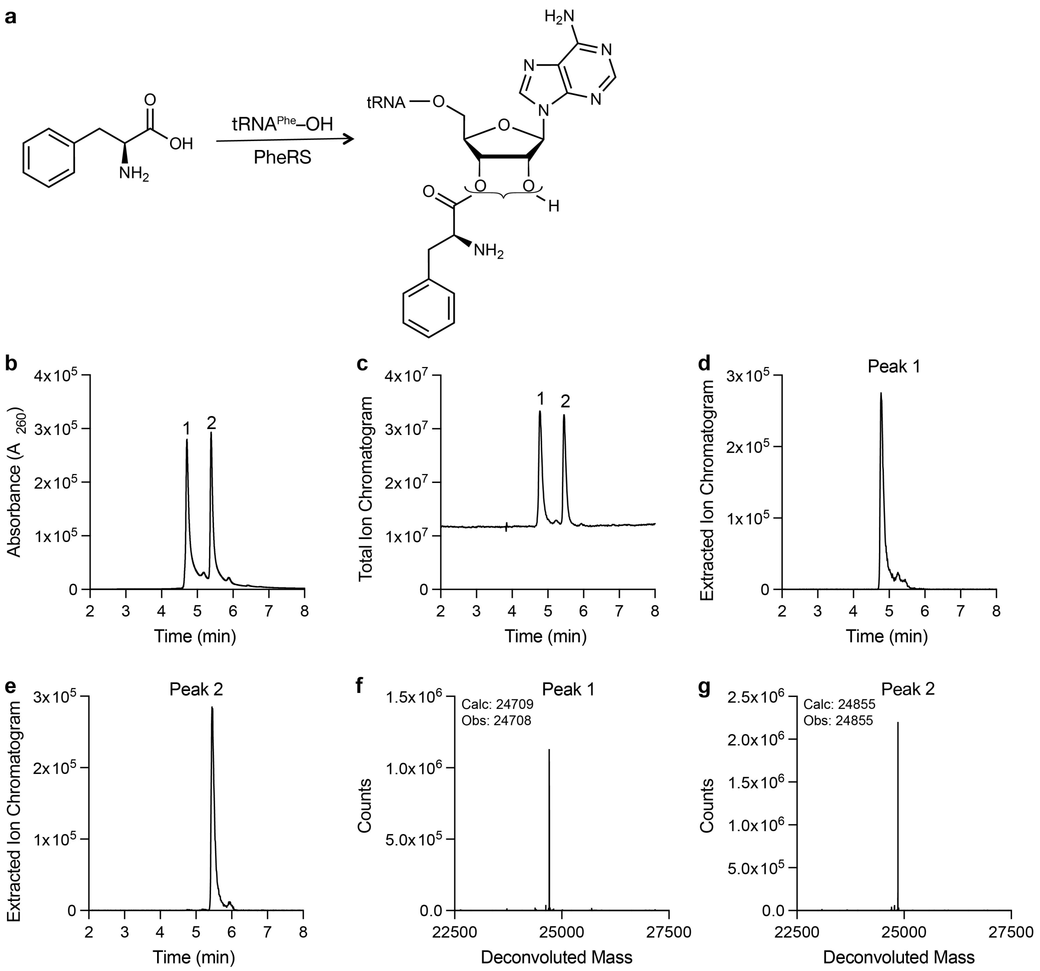

Figure S14. Intact tRNA LC-MS of phenylalanine (Phe) added to tRNA^{Phe} using PheRS.

LC-MS was used to determine the acylation efficiency of addition of Phe to tRNA^{Phe} (tRNA-OH) using PheRS. (a) Schematic showing mono-acylation of tRNA-OH with Phe. (b) Liquid chromatogram (LC) of acylation reaction. Peak 1 is tRNA-OH and peak 2 is tRNA-O-Phe. (c) Total ion chromatogram (TIC) of acylation reaction. (d) Extracted ion chromatogram (EIC) of peak 1, tRNA-OH. (e) EIC of peak 2, tRNA-O-Phe. (f) Mass spectra of peak 1 deconvoluted from m/z data. (g) Mass spectra of peak 2 deconvoluted from m/z data. Two additional replicates of each reaction were performed and analyzed by LC-MS. These raw and graphed data are included in the fair data files.

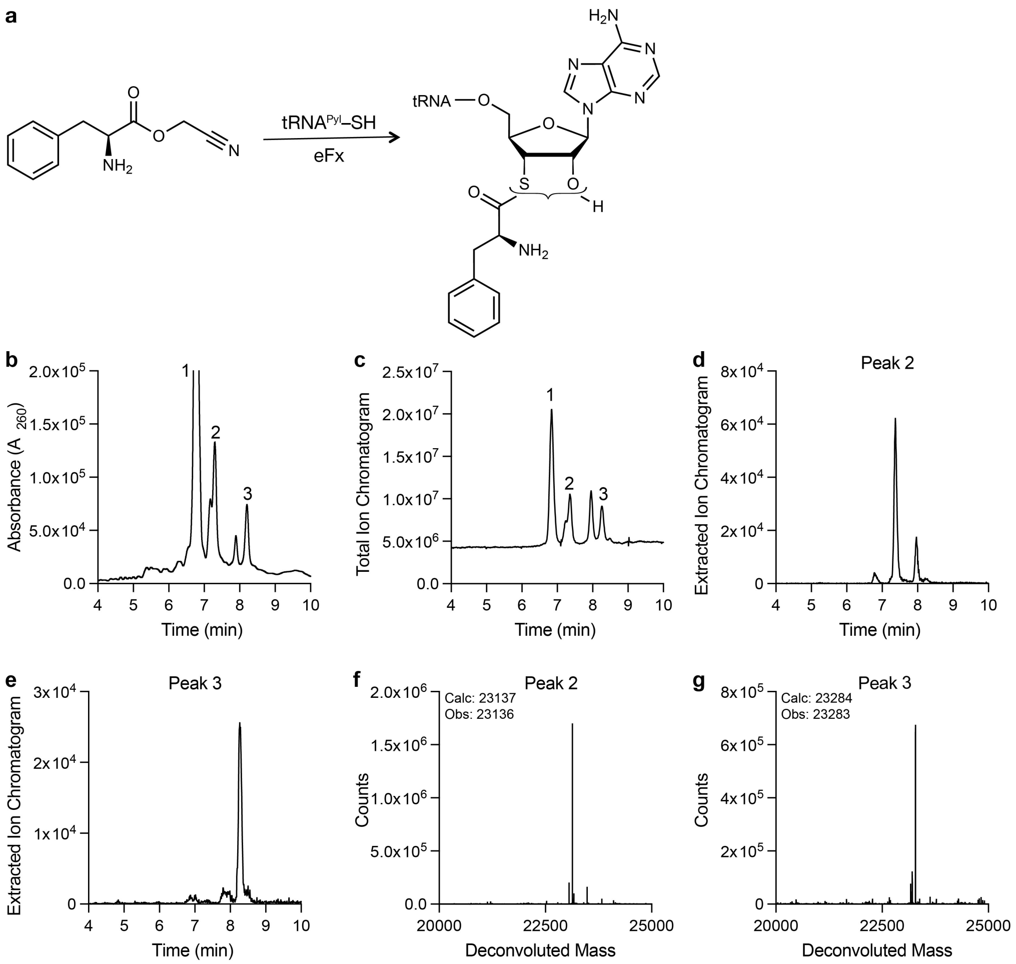

Figure S15. Intact tRNA LC-MS of phenylalanine (Phe) added to 3′-thio-tRNA^{Pyl} using eFx.
LC-MS was used to determine the acylation efficiency of addition of Phe from Phe-CME to 3′-thio-tRNA^{Pyl} (tRNA-SH) using the flexizyme eFx. (a) Schematic showing mono-acylation of tRNA-SH with Phe. (b) Liquid chromatogram (LC) of acylation reaction. Peak 1 is eFx, peak 2 is tRNA-SH and peak 3 is tRNA-S-Phe. (c) Total ion chromatogram (TIC) of acylation reaction. (d) Extracted ion chromatogram (EIC) of peak 2, tRNA-SH. (e) EIC of peak 3, tRNA-S-Phe. (f) Mass spectra of peak 2 deconvoluted from m/z data. (g) Mass spectra of peak 3 deconvoluted from m/z data. Two additional replicates of each reaction were performed and analyzed by LC-MS. These raw and graphed data are included in the fair data files.

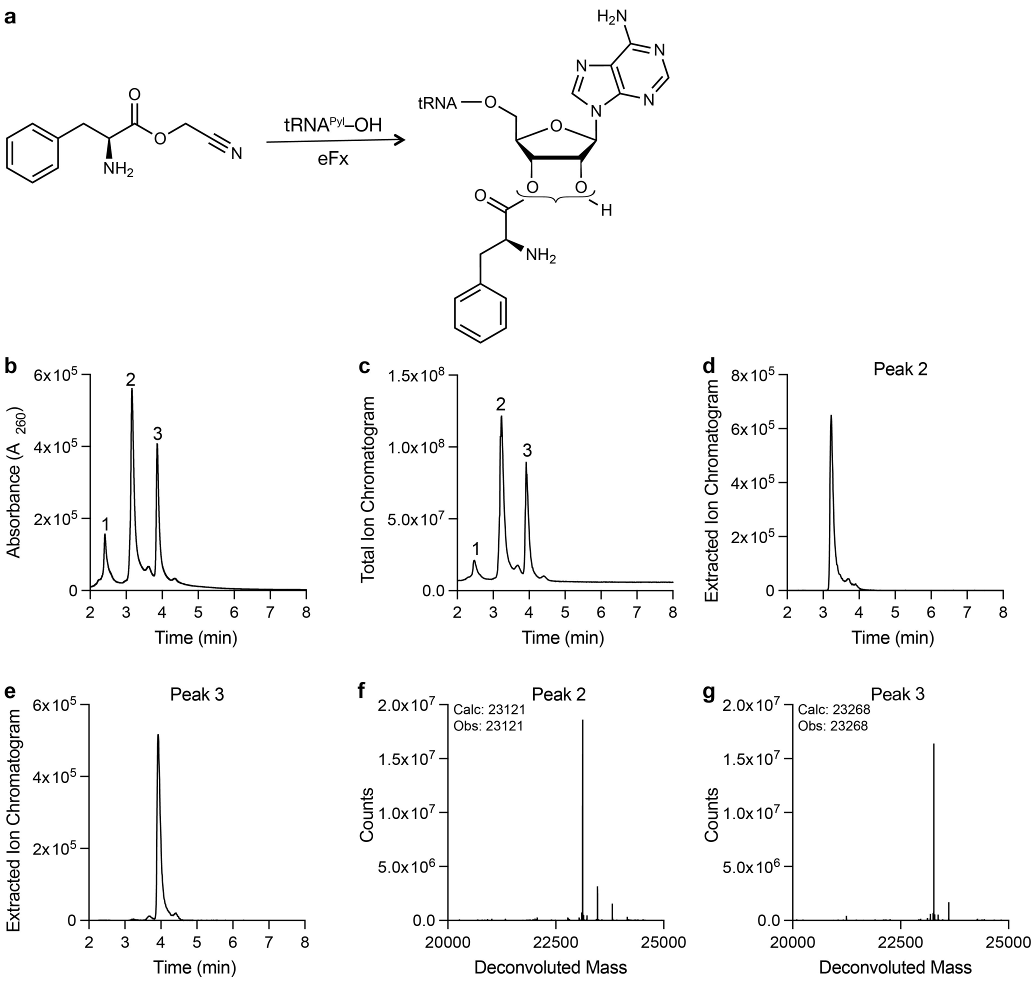

Figure S16. Intact tRNA LC-MS of phenylalanine (Phe) added to tRNA^{Pyl} using eFx.
LC-MS was used to determine the acylation efficiency of addition of Phe from Phe-CME to tRNA^{Pyl} (tRNA-OH) using the flexizyme eFx. (a) Schematic showing mono-acylation of tRNA-OH with Phe. (b) Liquid chromatogram (LC) of acylation reaction. Peak 1 is eFx, peak 2 is tRNA-OH and peak 3 is tRNA-O-Phe. (c) Total ion chromatogram (TIC) of acylation reaction. (d) Extracted ion chromatogram (EIC) of peak 2, tRNA-OH. (e) EIC of peak 3, tRNA-O-Phe. (f) Mass spectra of peak 2 deconvoluted from m/z data. (g) Mass spectra of peak 3 deconvoluted from m/z data. Two additional replicates of each reaction were performed and analyzed by LC-MS. These raw and graphed data are included in the fair data files.

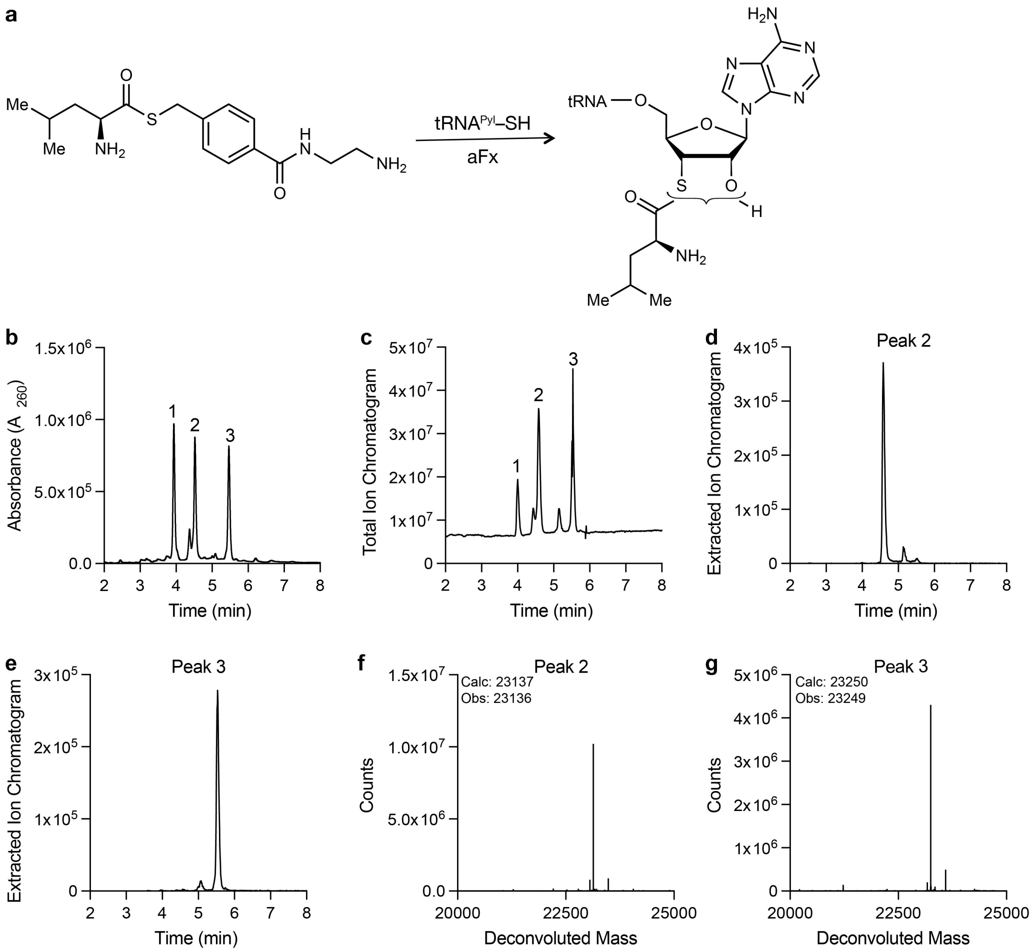

Figure S17. Intact tRNA LC-MS of leucine (Leu) added to 3′-thio-tRNA^{Pyl} using aFx.

LC-MS was used to determine the acylation efficiency of addition of Leu from Leu-ABT to 3′-thio-tRNA^{Pyl} (tRNA-SH) using the flexizyme aFx. (a) Schematic showing mono-acylation of tRNA-SH with Leu. (b) Liquid chromatogram (LC) of acylation reaction. Peak 1 is aFx, peak 2 is tRNA-SH and peak 3 is tRNA-S-Leu. (c) Total ion chromatogram (TIC) of acylation reaction. (d) Extracted ion chromatogram (EIC) of peak 2, tRNA-SH. (e) EIC of peak 3, tRNA-S-Leu. (f) Mass spectra of peak 2 deconvoluted from m/z data. (g) Mass spectra of peak 3 deconvoluted from m/z data. Two additional replicates of each reaction were performed and analyzed by LC-MS. These raw and graphed data are included in the fair data files.

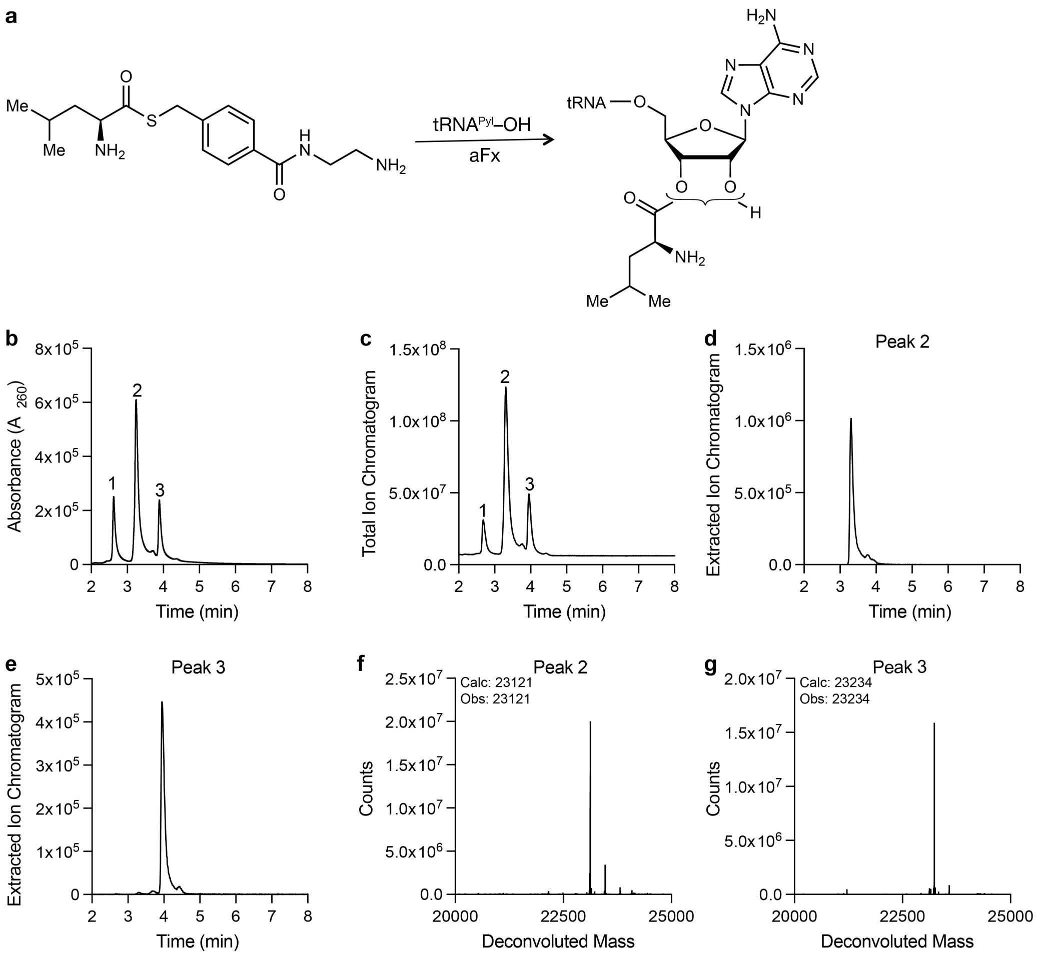

Figure S18. Intact tRNA LC-MS of leucine (Leu) added to tRNA^{Pyl} using aFx.

LC-MS was used to determine the acylation efficiency of addition of Leu from Leu-ABT to tRNA^{Pyl} (tRNA-OH) using the flexizyme aFx. (a) Schematic showing mono-acylation of tRNA-OH with Leu. (b) Liquid chromatogram (LC) of acylation reaction. Peak 1 is aFx, peak 2 is tRNA-OH and peak 3 is tRNA-O-Leu. (c) Total ion chromatogram (TIC) of acylation reaction. (d) Extracted ion chromatogram (EIC) of peak 2, tRNA-OH. (e) EIC of peak 3, tRNA-O-Leu. (f) Mass spectra of peak 2 deconvoluted from m/z data. (g) Mass spectra of peak 3 deconvoluted from m/z data. Two additional replicates of each reaction were performed and analyzed by LC-MS. These raw and graphed data are included in the fair data files.

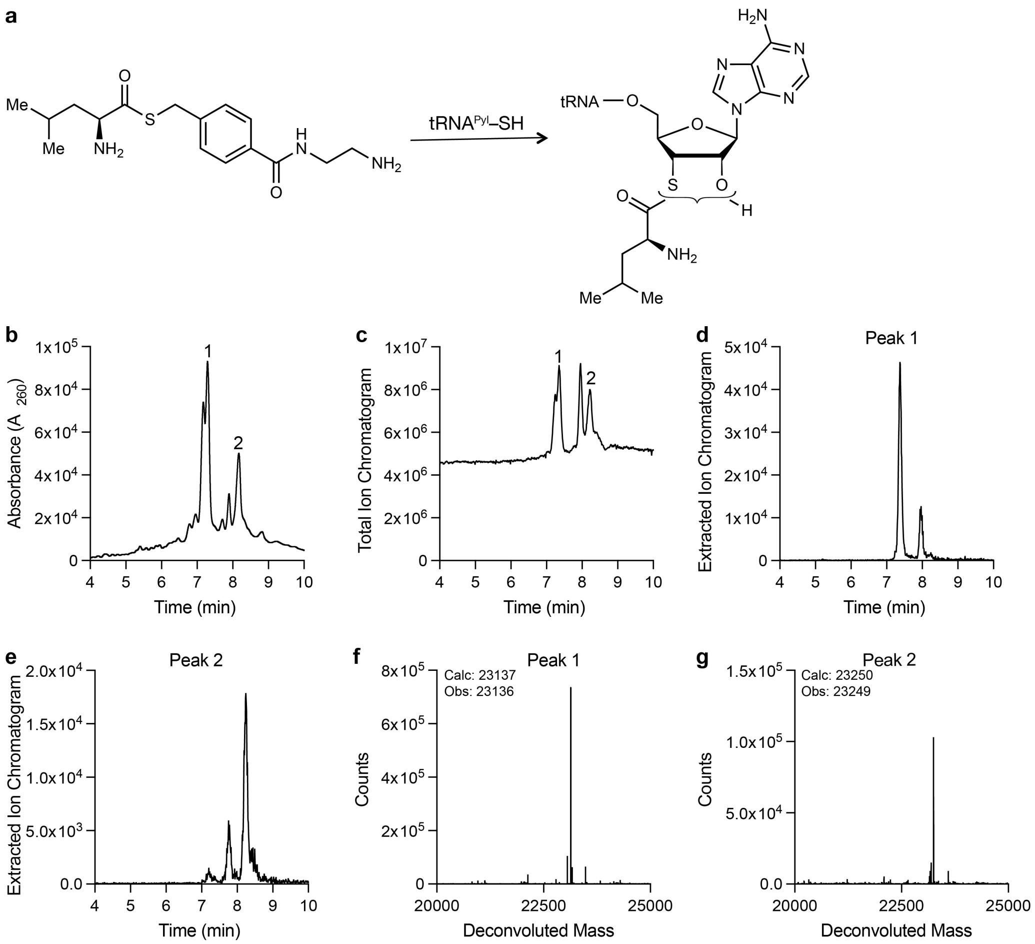

Figure S19. Intact tRNA LC-MS of leucine (Leu) added to 3′-thio-tRNA^{Pyl} using thioester exchange.
LC-MS was used to determine the acylation efficiency of addition of Leu from Leu-ABT to 3′-thio-tRNA^{Pyl} (tRNA-SH) by thioester exchange. (a) Schematic showing mono-acylation of tRNA-SH with Leu. (b) Liquid chromatogram (LC) of acylation reaction. Peak 1 is tRNA-SH and peak 2 is tRNA-S-Leu. (c) Total ion chromatogram (TIC) of acylation reaction. (d) Extracted ion chromatogram (EIC) of peak 1, tRNA-SH. (e) EIC of peak 2, tRNA-S-Leu. (f) Mass spectra of peak 1 deconvoluted from m/z data. (g) Mass spectra of peak 2 deconvoluted from m/z data. Two additional replicates of each reaction were performed and analyzed by LC-MS. These raw and graphed data are included in the fair data files.

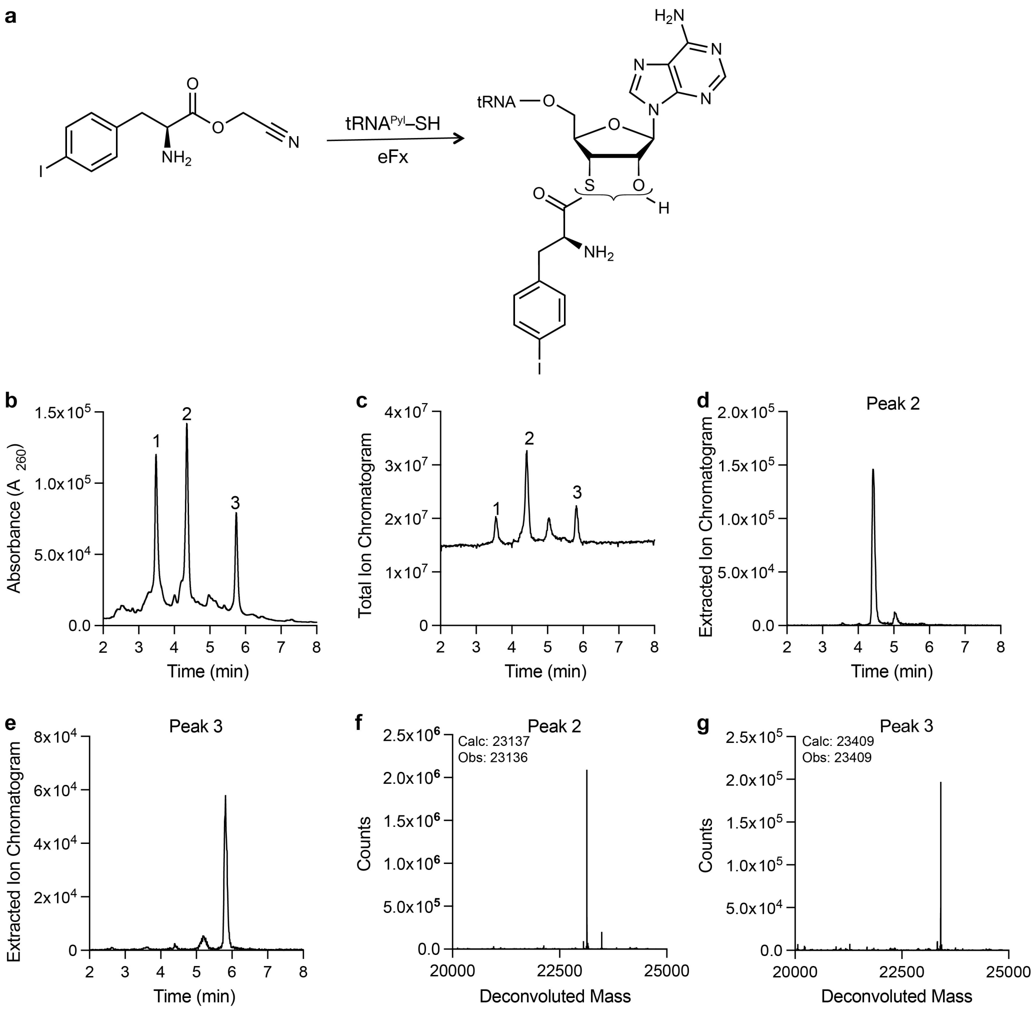

Figure S20. Intact tRNA LC-MS of iodo-phenylalanine (PheI) added to 3′-thio-tRNA^{Pyl} using eFx.
LC-MS was used to determine the acylation efficiency of addition of PheI from PheI-CME to 3′-thio-tRNA^{Pyl} (tRNA-SH) using the flexizyme eFx. (a) Schematic showing mono-acylation of tRNA-SH with PheI. (b) Liquid chromatogram (LC) of acylation reaction. Peak 1 is eFx, peak 2 is tRNA-SH and peak 3 is tRNA-S-PheI. (c) Total ion chromatogram (TIC) of acylation reaction. (d) Extracted ion chromatogram (EIC) of peak 2, tRNA-SH. (e) EIC of peak 3, tRNA-S-PheI. (f) Mass spectra of peak 2 deconvoluted from m/z data. (g) Mass spectra of peak 3 deconvoluted from m/z data. Two additional replicates of each reaction were performed and analyzed by LC-MS. These raw and graphed data are included in the fair data files.

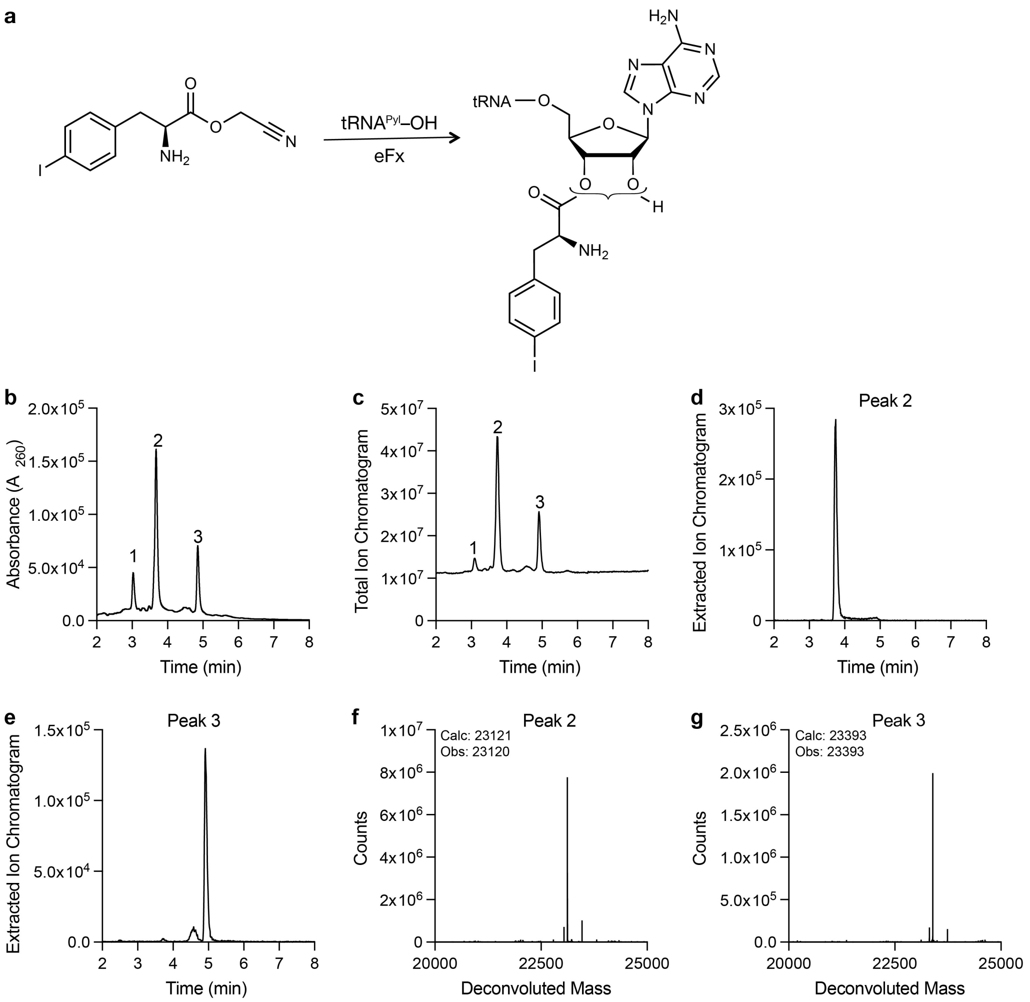

Figure S21. Intact tRNA LC-MS of iodo-phenylalanine (PheI) added to tRNA^{Pyl} using eFx.
LC-MS was used to determine the acylation efficiency of addition of PheI from PheI-CME to tRNA^{Pyl} (tRNA-OH) using the flexizyme eFx. (a) Schematic showing mono-acylation of tRNA-OH with PheI. (b) Liquid chromatogram (LC) of acylation reaction. Peak 1 is eFx, peak 2 is tRNA-OH and peak 3 is tRNA-O-PheI. (c) Total ion chromatogram (TIC) of acylation reaction. (d) Extracted ion chromatogram (EIC) of peak 2, tRNA-OH. (e) EIC of peak 3, tRNA-O-PheI. (f) Mass spectra of peak 2 deconvoluted from m/z data. (g) Mass spectra of peak 3 deconvoluted from m/z data. Two additional replicates of each reaction were performed and analyzed by LC-MS. These raw and graphed data are included in the fair data files.

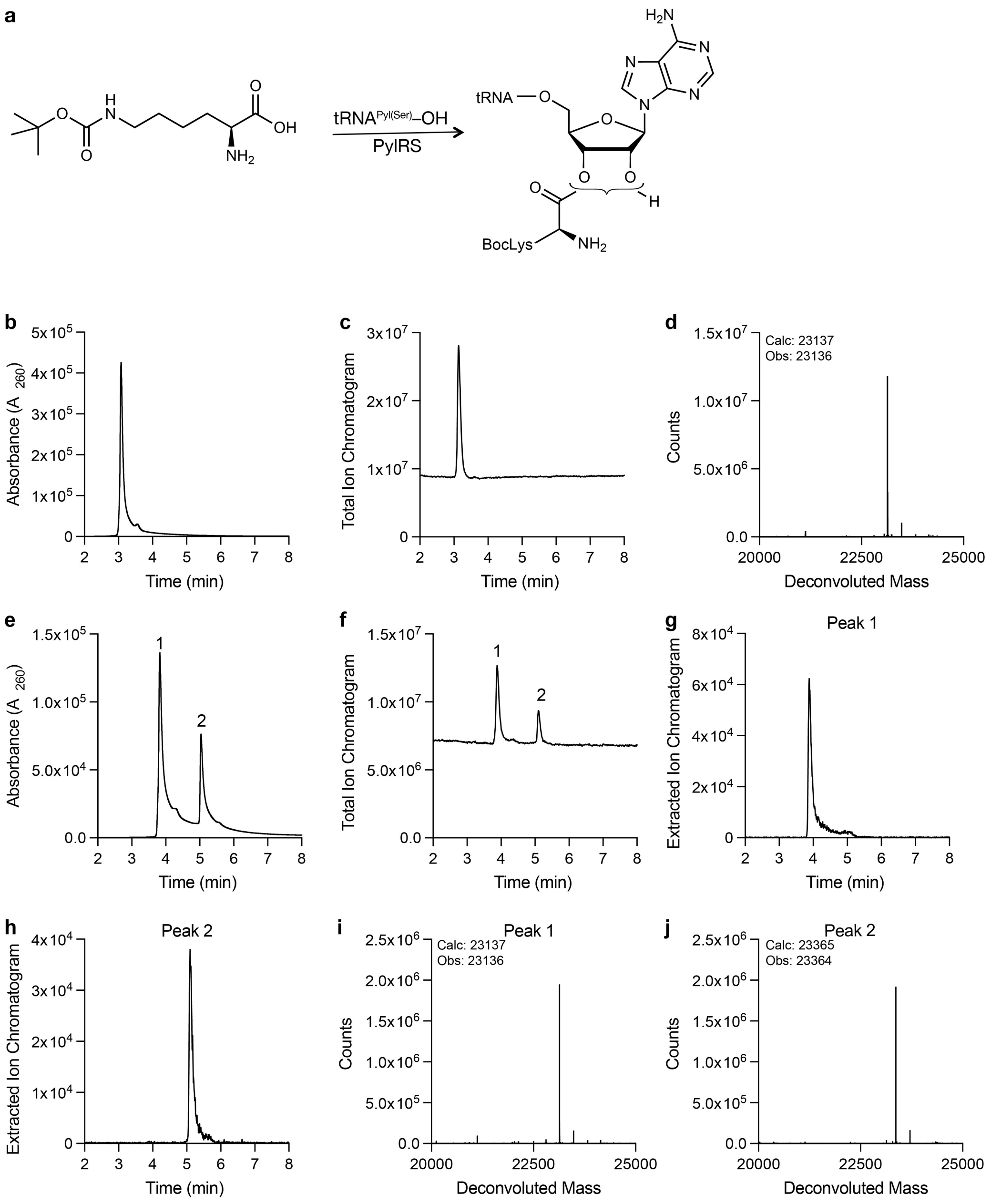

Figure S22. Intact tRNA LC-MS of N_ε-boc-L-lysine (BocLys) added to tRNA$^{Pyl(Ser)}$ using PylRS.

LC-MS was used to determine the acylation efficiency of addition of BocLys to a tRNA^{Pyl} containing a serine anticodon (tRNA$^{Pyl(Ser)}$, tRNA-OH) using PylRS. (a) Schematic showing mono-acylation of tRNA-OH with BocLys. (b) Liquid chromatogram (LC) of tRNA^{Pyl}-OH. (c) Total ion chromatogram (TIC) of tRNA^{Pyl}-OH. (d) Mass spectra of tRNA^{Pyl}-OH deconvoluted from m/z data. (e) Liquid chromatogram (LC) of acylation reaction. Peak 1 is tRNA-OH and peak 2 is tRNA-O-BocLys. (f) Total ion chromatogram (TIC) of acylation reaction. (g) Extracted ion chromatogram (EIC) of peak 1, tRNA-OH. (h) EIC of peak 2, tRNA-O-BocLys. (i) Mass spectra of peak 1 deconvoluted from m/z data. (j) Mass spectra of peak 2 deconvoluted from m/z data.

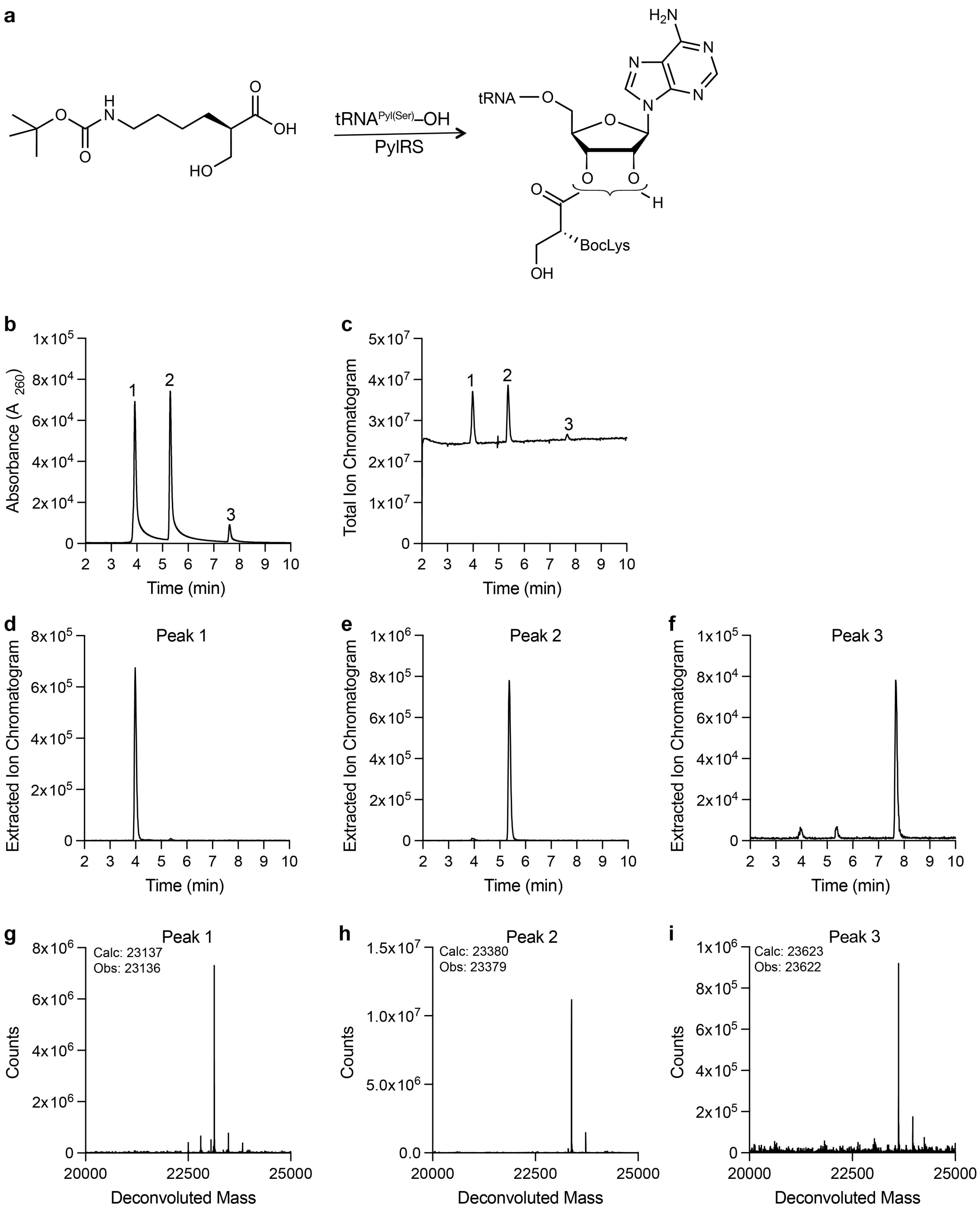

Figure S23. Intact tRNA LC-MS of (*R*)-β^2-OH-N_ε-BocLys ((*R*)-β^2-OH) added to tRNA$^{Pyl(Ser)}$ using PylRS. LC-MS was used to determine the acylation efficiency of addition of (*R*)-β^2-OH to a tRNA^{Pyl} containing a serine anticodon (tRNA$^{Pyl(Ser)}$, tRNA-OH) using PylRS. (a) Schematic showing mono-acylation of tRNA-OH with (*R*)-β^2-OH. (b) Liquid chromatogram (LC) of tRNA-O-(*R*)-β^2-OH. Peak 1 is tRNA-OH, peak 2 is tRNA-O-(*R*)-β^2-OH, and peak 3 is tRNA-O-(*R*)-β^2-OH (x2). (c) Total ion chromatogram (TIC) of tRNA^{l}-O-(*R*)-β^2-OH. (d) Extracted ion chromatogram (EIC) of peak 1, tRNA-OH. (e) EIC of peak 2, tRNA-O-(*R*)-β^2-OH. (f) EIC of peak 3, tRNA-O-(*R*)-β^2-OH(x2). (g) Mass spectra of peak 1 deconvoluted from m/z data. (h) Mass spectra of peak 2 deconvoluted from m/z data. (i) Mass spectra of peak 3 deconvoluted from m/z data.

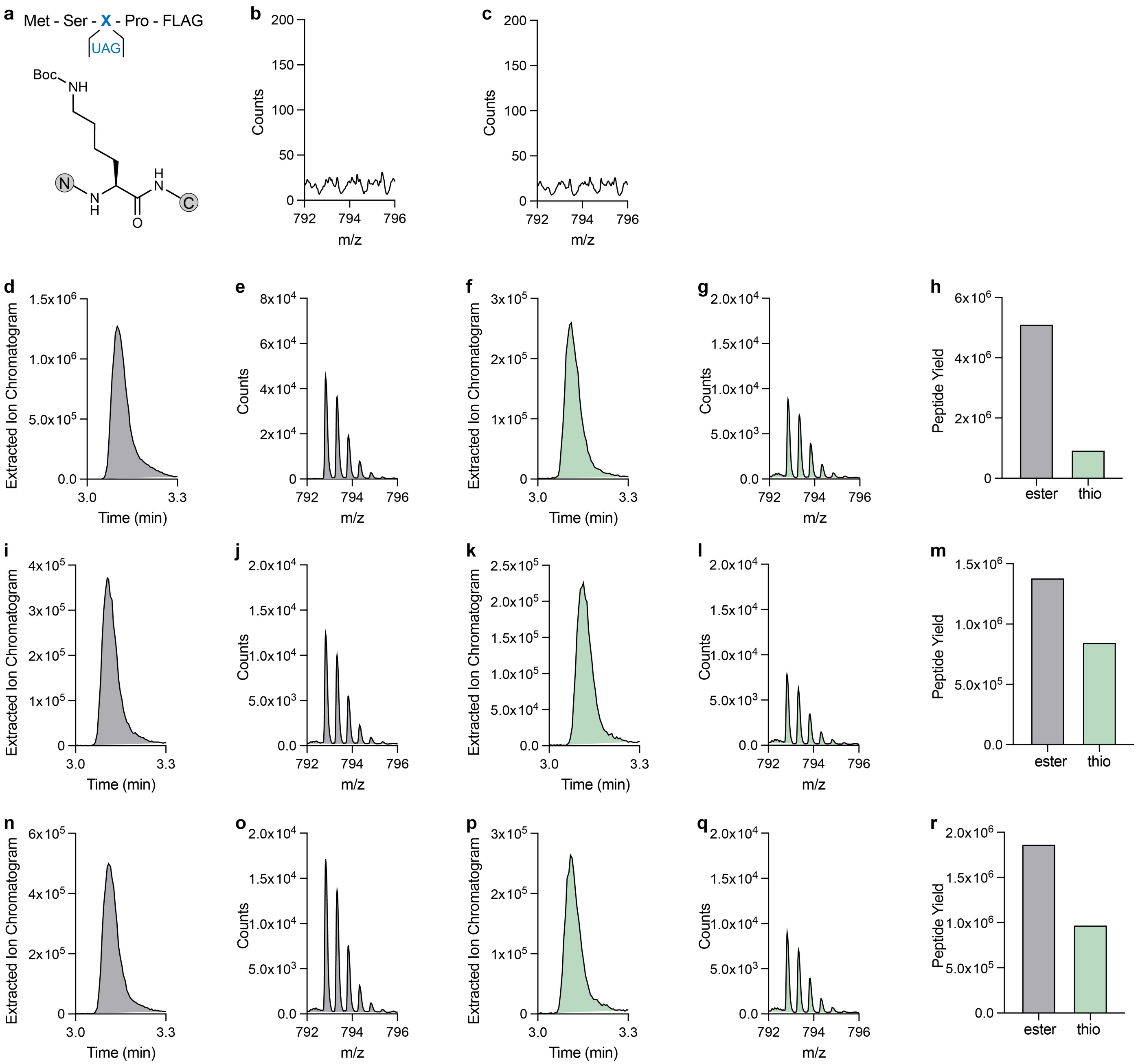

Figure S24. Incorporation of BocLys into a peptide from either 3′-thio-$tRNA^{Pyl}$ or $tRNA^{Pyl}$.

LC-MS was used to determine the incorporation of BocLys into a peptide (Met-Ser-BocLys-Pro-Asp-Tyr-Lys-Asp-Asp- Asp-Asp-Lys) from either a 3′-thio-$tRNA^{Pyl}$ or $tRNA^{Pyl}$. Extended and replicate data for Figure 4c. (a) Sequence of the peptide being made by IVTT with the recoded stop codon highlighted in blue. The structure of BocLys with (N) and (C) indicating the N- and C-termini of the peptide. (b) The m/z spectra of a no-$tRNA^{Pyl}$ control IVTT reaction showing no presence of the desired peptide. (c) The m/z spectra of an uncharged $tRNA^{Pyl}$ control IVTT reaction showing no presence of the desired peptide. (d) Extracted Ion Chromatogram (EIC) of encoded peptide with BocLys originating from $tRNA^{Pyl}$-O-BocLys. (e) The m/z spectra of the peptide in d. (f) EIC of encoded peptide with BocLys originating from $tRNA^{Pyl}$-S-BocLys. (g) The m/z spectra of the peptide in f. (h) EIC integration values quantifying the relative yield of the peptides. (i-r) Two replicate data sets of d-h.

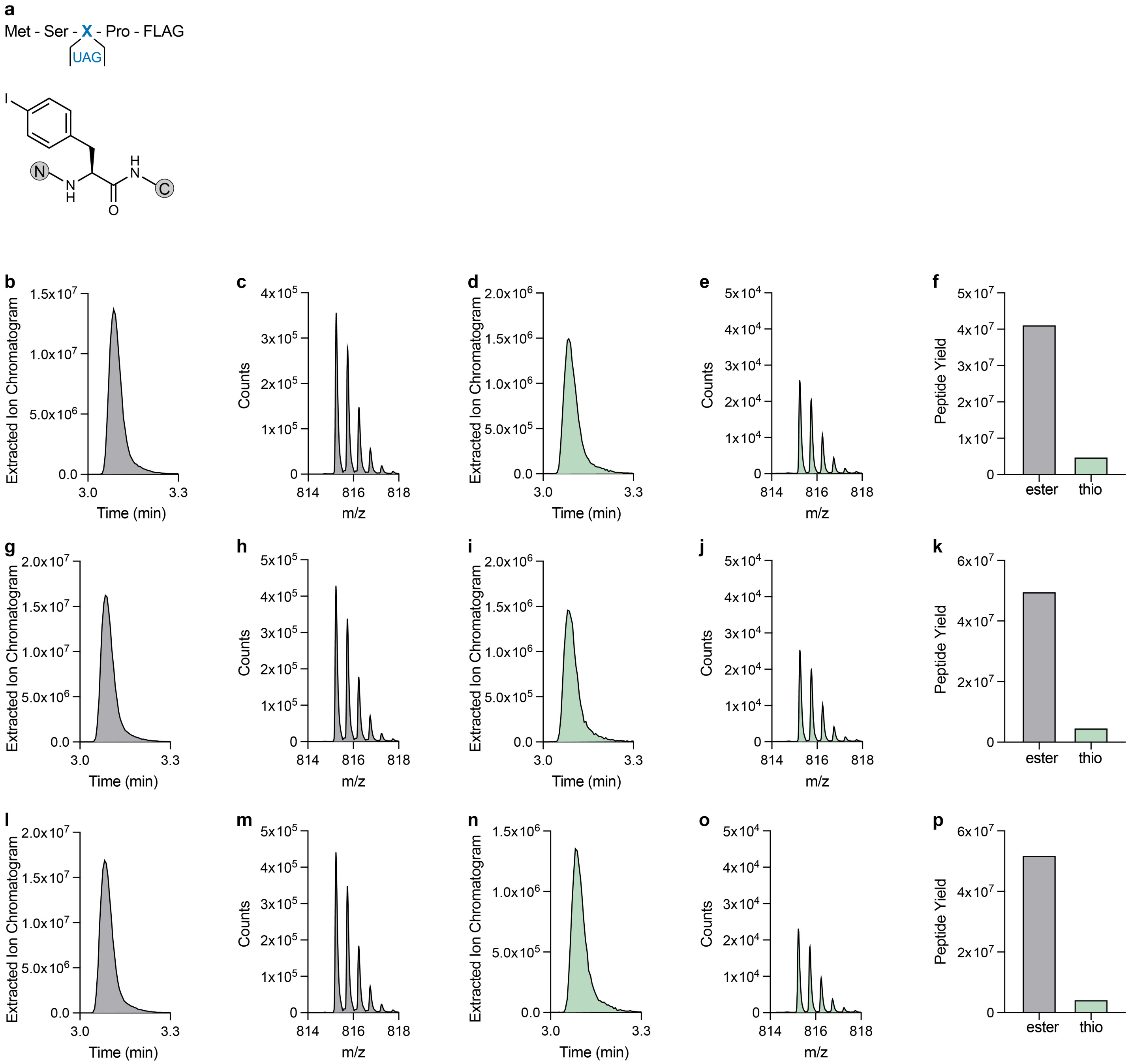

Figure S25. Incorporation of PheI into a peptide from either 3′-thio-tRNA^{Pyl} or tRNA^{Pyl}.
LC-MS was used to determine the incorporation of PheI into a peptide (Met-Ser-PheI-Pro-Asp-Tyr-Lys-Asp-Asp-Asp-Asp-Lys) from either a 3′-thio-tRNA^{Pyl} or tRNA^{Pyl}. Extended and replicate data for Figure 4d. (a) Sequence of the peptide being made by IVTT with the recoded stop codon highlighted in blue. The structure of PheI with (N) and (C) indicating the N- and C-termini of the peptide. (b) Extracted Ion Chromatogram (EIC) of encoded peptide with PheI originating from tRNA^{Pyl}-O-PheI. (c) The m/z spectra of the peptide in b. (d) EIC of encoded peptide with PheI originating from tRNA^{Pyl}-S-PheI. (e) The m/z spectra of the peptide in d. (f) EIC integration values quantifying the relative yield of the peptides. (g-p) Two replicate data sets of b-f.

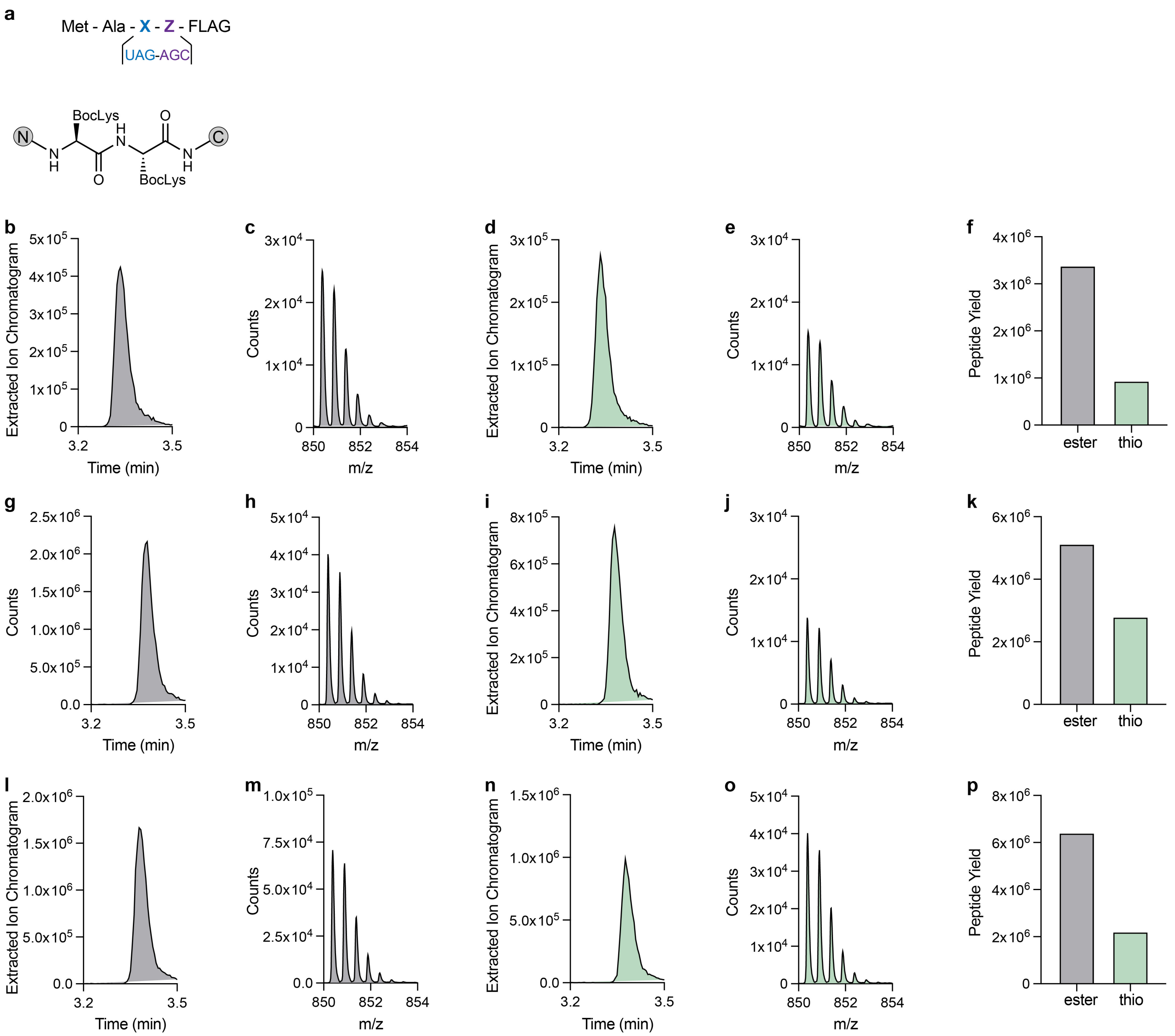

Figure S26. Incorporation of two BocLys monomers into a peptide from either 3′-thio-tRNA^{Pyl} or tRNA^{Pyl}.

LC-MS was used to determine the incorporation of two BocLys unnatural amino acids into a peptide (Met-Ala-BocLys-BocLys-Asp-Tyr-Lys-Asp-Asp-Asp-Asp-Lys) from either a 3′-thio-tRNA^{Pyl} or tRNA^{Pyl} for the first BocLys and a tRNA$^{Pyl(Ser)}$ for the second BocLys. Extended and replicate data for Figure 4e. (a) Sequence of the peptide being made by IVTT with the recoded stop codon highlighted in blue and recoded serine codon highlighted in purple. The structure of the peptide with (N) and (C) indicating the N- and C-termini. (b) Extracted Ion Chromatogram (EIC) of encoded peptide with the first BocLys originating from tRNA^{Pyl}-O-BocLys. (c) The m/z spectra of the peptide in b. (d) EIC of encoded peptide with the first BocLys originating from tRNA^{Pyl}-S-BocLys. (e) The m/z spectra of the peptide in d. (f) EIC integration values quantifying the relative yield of the peptides. (g-p) Two replicate data sets of b-f.

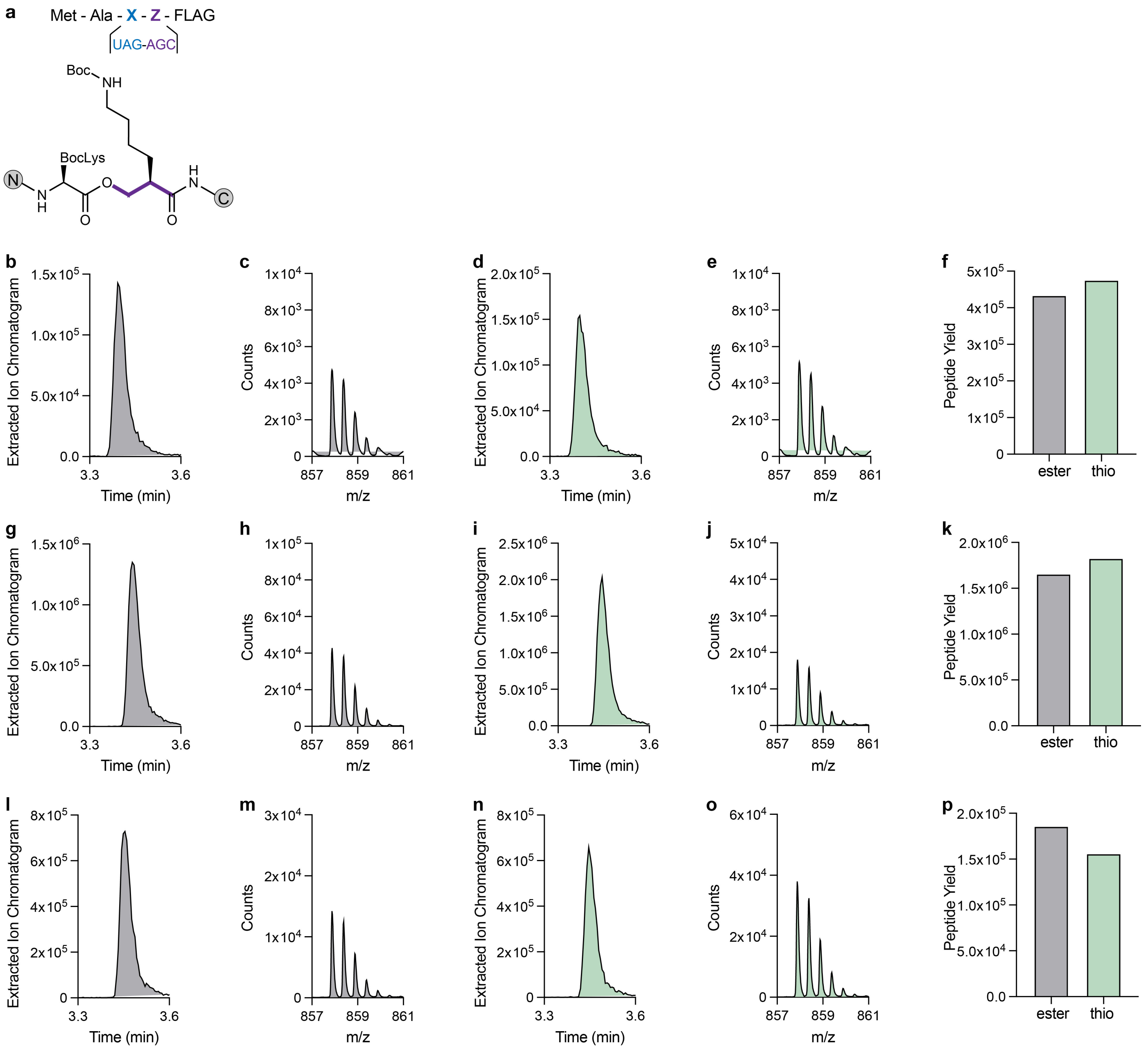

Figure S27. Incorporation of (*R*)-β²-OH into a peptide following BocLys incorporated from either 3′-thio-tRNA^{Pyl} or tRNA^{Pyl}.

LC-MS was used to determine the incorporation of BocLys and (*R*)-β²-OH unnatural amino acids into a peptide (Met-Ala-BocLys-(*R*)-β²-O-Asp-Tyr-Lys-Asp-Asp-Asp-Asp-Lys) from either a 3′-thio-tRNA^{Pyl} or tRNA^{Pyl} for BocLys and a tRNA$^{Pyl(Ser)}$ for (*R*)-β²-OH. Extended and replicate data for Figure 4f. (a) Sequence of the peptide being made by IVTT with the recoded stop codon highlighted in blue and recoded serine codon highlighted in purple. The structure of the peptide with (N) and (C) indicating the N-and C-termini and the β-backbone highlighted in purple. (b) Extracted Ion Chromatogram (EIC) of encoded peptide with BocLys originating from tRNA^{Pyl}-O-BocLys. (c) The m/z spectra of the peptide in b. (d) EIC of encoded peptide with BocLys originating from tRNA^{Pyl}-S-BocLys. (e) The m/z spectra of the peptide in d. (f) EIC integration values quantifying the relative yield of the peptides. (g-p) Two replicate data sets of b-f.

Supplementary Tables

Table 1: DNA sequences.

Name	Sequence 5'-->3'
E. coli tRNA-fMet	AATTCCTGCAGTAATACGACTCACTATAGGCGGGGTGGAGCAGCCTGGTAGCTCGTCGGGCTCATAACCCGAAGGTCGTCGGTTCAAATCCGGCCCCCGCAACCA
E. coli tRNA-Phe	AATTCCTGCAGTAATACGACTCACTATAGCCCGGATAGCTCAGTCGGTAGAGCAGGGGATTGAAAATCCCCGTGTCCTTGGTTCGATTCCGAGTCCGGGCACCA
M. alvus tRNA-Pyl	AATTCCTGCAGTAATACGACTCACTATAGGGGGACGGTCCGGCGACCAGCGGGTCTCTAAAACCTAGCCAGCGGGGTTCGACGCCCCGGTCTCTCGCCA
M. alvus tRNA-Pyl^{Ser}	ATTCCTGCAGTAATACGACTCACTATAGGGGGACGGTCCGGCGACCAGCGGGTCTGCTAAACCTAGCCAGCGGGGTTCGACGCCCCGGTCTCTCGCCA
Peptide-1 Template	GCGAATTAATACGACTCACTATAGGGTTAACTTTAACAAGGAGAAAAACATGAGCTAGCCGGACTACAAAGACGATGACGACAAGTAACTAGCATAACCCCTCTCTAAACGGAGGGGTTTAGTCA
Peptide-2 Template	GCGAATTAATACGACTCACTATAGGGTTAACTTTAACAAGGAGAAAAACATGGCGTAGAGCGACTACAAAGACGATGACGACAAGTAACTAGCATAACCCCTCTCTAAACGGAGGGGTTTAGTCA
tRNA Fwd Primer	AATTCCTGCAGTAATACGACTCAC
tRNA-fMet (-A) Rev Primer	mGGTTGCGGGGGCCG
tRNA-Phe (-A) Rev Primer	mGGTGCCCGGACTCG
tRNA-Pyl (-A) Rev Primer	mGGCGAGAGACCGGGC
tRNA-Pyl Rev Primer	TmGGCGAGAGACCGGGC
tRNA-fMet Rev Primer	TmGGTTGCGGGGGCCG
tRNA-Phe Rev Primer	TmGGTGCCCGGACTCG
Templates Fwd Primer	GCGAATTAATACGACTCACTATAGGGTTAACTTTAAC
Templates Rev Primer	TGACTAAACCCCTCCGTTTAGAGAGG

m: 2′-O-methyl modified

Table 2: Peptide Sequences.

Name	Sequence
Peptide-1	M-S-X-P-D-Y-D-D-D-D-K
Peptide-2	M-A-X-Z-D-Y-D-D-D-D-K

References

1. Sun, S., Yoshida, A. & Piccirilli, J. A. Synthesis of 3’-thioribonucleosides and their incorporation into oligoribonucleotides via phosphoramidite chemistry. *RNA* **3**, 1352–1363 (1997).
2. Choi, K. W. & Brimble, M. A. Synthesis of spiroacetal-nucleosides as privileged natural product-like scaffolds. *Org. Biomol. Chem.* **7**, 1424–1436 (2009).
3. Williams, D. M. & Harris, V. H. Phosphorous Methods in Nucleotide Chemistry. in *Organophosphorus Reagents: A Practical Approach in Chemistry* (ed. Murphy, P. J.) 237–272 (Oxford University Press, Oxford, New York, 2004).
4. Busnel, O. *et al.* Synthesis and evaluation of new ω-borono-α-amino acids as rat liver arginase inhibitors. *Bioorg. Med. Chem.* **13**, 2373–2379 (2005).
5. Pretsch, E., Bühlmann, P. & Badertscher, M. *Structure Determination of Organic Compounds: Tables of Spectral Data*. (Springer-Verlag, Berllin Heidelberg, 2009).
6. Nissley, A. J., Penev, P. I., Watson, Z. L., Banfield, J. F. & Cate, J. H. D. Rare ribosomal RNA sequences from archaea stabilize the bacterial ribosome. *Nucleic Acids Res.* **51**, 1880–1894 (2023).
7. Kao, C., Zheng, M. & Rüdisser, S. A simple and efficient method to reduce nontemplated nucleotide addition at the 3 terminus of RNAs transcribed by T7 RNA polymerase. *RNA* **5**, 1268–1272 (1999).
8. Majumdar, C., Walker, J. A., Francis, M. B., Schepartz, A. & Cate, J. H. D. Aminobenzoic Acid Derivatives Obstruct Induced Fit in the Catalytic Center of the Ribosome. *ACS Cent. Sci.* **9**, 1160–1169 (2023).
9. Fricke, R. *et al.* Expanding the substrate scope of pyrrolysyl-transfer RNA synthetase enzymes to include non-α-amino acids in vitro and in vivo. *Nat. Chem.* **15**, 960–971 (2023).
