# Supplementary material for: Thioesters Support Efficient Protein Biosynthesis by the Ribosome
Source: ACS Cent Sci. 2025 Jan 30;11(3):404–12. doi: 10.1021/acscentsci.4c01698 (PMC11950863; doi:10.1021/acscentsci.4c01698)
Supplement: Supplementary file 2 — oc4c01698_si_002.pdf [file oc4c01698_si_002.pdf]

oc-2024-01698a.R1

Name: Peer Review Information for "Thioesters support efficient protein biosynthesis by the ribosome"

First Round of Reviewer Comments

Reviewer: 1

Comments to the Author

The study titled “Thioesters support efficient protein biosynthesis by the ribosome” by Alexandra Kent et al explores the use of thioesters in incorporating several natural and non-natural amino acids into proteins by ribosomal synthesis. They replaced oxo-esters with thioesters in acyl-tRNA substrates and used E coli ribosomal machinery to evaluate their substrate specificity in generating oligopeptides during in vitro translation. For this purpose, they have synthesized 3’-thio-3’-deoxyadenosine triphosphate and showed that it supports all the 3 steps in protein translation: formation of 3’-thio-tRNA using a CCA-adding enzyme, aminoacylation of 3’-thio-tRNA using enzymes/flexizymes and peptide bond formation using ribosome. Despite differences from oxo-esters in bond lengths, hydrogen bonding capacity and electrophilicity, efficient translation using thioester-tRNA substrates were observed. This suggests that changes in hydrogen bonding potential are not disruptive enough to inhibit translation. This work opens up the possibility of employing thioesters to incorporate natural and non-natural amino acids by the translational machinery for sequence-defined polypeptide synthesis. Overall, the work is well planned and well-executed, experiments were carefully performed, and data was presented lucidly. This study could be recommended for publication in ACS Central Science after addressing a few minor comments.

A few minor observations are:

1. Although the 3’-thio tRNAs acylated with various amino acids were recognized and utilized by the E. coli ribosomal machinery to form oligopeptides in comparable yields as of native oxo-ester tRNAs, I was wondering whether it affects the kinetics of the translation reaction? Authors may want to comment on this fact as the ribosomal protein synthesis is an incredibly optimized process with oxo-ester tRNAs and whether this O to S change has any impact on the overall efficiency of the reaction in synthesizing longer proteins.

2. Please include ppm errors in the mass spectrums provided in the text.

3. Is there any experimental or theoretical evidence for the proposed role EF-Tu in preferential binding to 3'-thioester during translation?

Thank you

Reviewer: 2

Comments to the Author

This is an excellent paper from Cate and co-workers that describes the synthesis 3'-thio-3'deoxy-adenosine and incorporation into tRNAs for ribosomal translation. IN a series of well-laid out experiments, they convince this reviewer that the ribosomal synthesis using these 3'-thio-tRNAs proceeds via intermediacy of the corresponding tRNA-thioester. The work opens doors and certainly begs additional questions, but it is substantive and complete in its demonstration of the ability of these unnatural sugars to mediate ribosomal translation. This work would make an appropriate addition to the pages of ACS Central Science. I have only a few minor concerns:

1. Yields for the 3'-thio-3'deoxy-adenosine should be presented in Figure 1d (or below) in the main text, not just SI. It would also be helpful to report the authors yield for compound 2 from 1 (according to the literature protocol.

2. The authors seem to eschew the more quantitative translation assays that have recently been reported by Genentech (Chan, A. I. et al. ACS Chem. Biol. 18, 81–90, 2023) and, more recently, Matt Hartmans (Kerestesy, G. N., et al., ACS Synth. Biol. 13, 119–128, 2024) group. This reviewer would prefer if the authors results were accompanied by such assay data. In lieu of this, please add a rationale for the choice of MS over these more quantitative assays. Additionally, please include all MS parameters used for analysis here (injection volume, gas flow and temp, nebulizer pressure, VCap voltage, fragmentor voltage, and skimmer voltage). This would help improve reproducibility of these types of results.

3. In Figure 4, it would be more helpful to see the integrated EIC scale represented as counts (absolute units) as they are in the SI. This does not change intensity of the overlays and authors can keep the ratio bars on the side, but allows the reader to see differences in the translation efficiencies between the substrates (e.g. the alpha-hydroxy substrate).

4. At the end of the manuscript, the authors allude to the idea that these 3'-thio-tRNAs might enable additional nucleophiles. Several additional examples in Figure 4 would help solidify the results. Since the authors are already using Flexizyme, 1-2 more alpha-hydroxy acids could help prove the pattern. Also, an N-methyl amino acid would be significantly appreciated here and would go a long way in cementing interest in this paper for the broader community. The present substrate scope is far too limited.

Reviewer: 3

Comments to the Author

Thioesters are more reactive than oxygen esters, resulting in faster hydrolysis rates under standard biochemical conditions (neutral pH, 37°C, physiological buffer). Their hydrolysis rates vary depending on their structure and environmental factors. For instance, certain alkyl-thioesters have half-lives of approximately 40 hours at pH 7 and room temperature, though this rate can shift with changes in temperature or pH. In contrast, peptide bonds (amides), chosen by nature as the backbone for proteins, are significantly more stable, with estimated half-lives of around 1,000 years under the same conditions. This inherent reactivity of thioesters rationalizes their widespread role as intermediates in metabolic processes, rather than as chemical linkages in biological polymers.

Nonetheless, thioesters such as thiol esters of amino acids, play a significant role in peptide biosynthesis as intermediates, mimicking aspects of canonical translation. They are speculated to be used as intermediates in non-canonical protein synthesis, even by ribosomes under controlled conditions. This is possible because thioesters are highly reactive acyl donors, owing to their polarizable sulfur atom, which weakens the bond between the carbonyl carbon (which is more electrophilic) and the leaving group—a feature that underpins their role in metabolism.

Non-ribosomal peptide synthetases (NRPSs) is the best documented example of efficient use thioesters as intermediates in peptide biosynthesis. This open-up the possibilities to transfer this chemistry to ribosomal peptide and protein synthesis, leveraging their efficient coupling capabilities. Engineered aminoacyl-tRNA synthetases could theoretically activate thioester derivatives to load non-canonical amino acids (ncAAs) onto tRNAs.

The activation of amino acids in both ribosomal and NRPS systems involves the formation of aminoacyl-adenylates. However, the transfer mechanism differs in NRPSs, the aminoacyl-adenylate is transferred to the thiol group of a peptidyl carrier protein, forming a thioester linkage. In contrast, ribosomal synthesis involves transfer to tRNA, resulting in a classical ester linkage.

In this context, the manuscript of Kent et al., reports an interesting innovation that demonstrate that tRNAs acylated with a thioester, instead of the traditional ester linkage, to both cAAa and ncAAs can be utilized by the existing translation machinery for the synthesis of sequence-defined polymers. In particular, the authors synthesized 3'-thio-3'-deoxyadenosine triphosphate from xylose and demonstrated its incorporation into truncated tRNAs via the E. coli CCA-adding enzyme, enabling the generation of 3'-thio-tRNAs acylated with α- and non-α-amino acids. These 3'-thio-tRNAs were successfully utilized by wild-type E. coli ribosomes during in vitro translation.

In particular, the 3'-OH on the 3'-terminal adenosines of a tRNA were replaced with a 3'-SH, and its performance was evaluated in tRNA extension, aminoacylation, and ribosome-mediated peptide bond formation. All three reactions were fully supported by thioesters, with yields comparable to those of oxo-ester tRNAs. The 3'-thio-ATP is a substrate for E. coli tRNA nucleotidyltransferase, a

CCA-adding enzyme, to add 3'-thio-3'-deoxyadenosine to the 3'-end of truncated tRNAs generated using in vitro transcription.

The central novelty of the paper is the use of native chemical ligation as evidence for the presence of thioesters. The authors investigated the dynamic interconversion between 3'-thioesters and 2'-oxo-esters on acylated tRNAs, finding that while the 2'-oxo-ester is thermodynamically favored, transient formation of the 3'-thioester occurs under kinetic control. This transient state was confirmed through native chemical ligation, resulting in dipeptide formation, despite the predominance of the 2'-oxo-ester observed via NMR. Translation efficiency in the ribosomal PTC was assessed using tRNAs with thioester or oxoester linkages, revealing significantly higher peptide yields with oxoester-linked tRNAs compared to thioester-linked tRNAs, expectedly demonstrating the superior compatibility of oxoesters in translation. In particular, in vitro ribosomal translation was shown to tolerate a single O-to-S substitution at the P-site tRNA when incorporating non-canonical α-amino acids, with oxoester-linked tRNAs producing higher yields of peptides containing successive BocLys residues. However, when incorporating a β2-hydroxy acid monomer, yields were equivalent regardless of the linkage type, though overall efficiency was reduced compared to incorporating two BocLys monomers.

In conclusion, thioesters were explored as replacements for esters in aminoacyl-tRNAs, leveraging their increased electrophilicity and potential role in peptide (and possibly fatty acid) formation. The key contribution of this approach lies in demonstrating that ribosomal translation machinery can tolerate the substitution of the ester bond, thereby facilitating the incorporation of less nucleophilic ncAAs via thioesters.

Technically, the chemical syntheses and aminoacylations of 3'-thio-tRNAs were thoroughly documented and performed with appropriate citation of relevant literature. However, the manuscript does not clearly delineate the novelty or advantages of this method compared to established approaches, such as native chemical ligation, expressed protein ligation, or the use of flexizymes. Furthermore, while evolutionary implications are discussed, the practical applications of this approach are insufficiently addressed, with only broad, unspecific statements regarding utility in synthetic biology.

Finally, the relevance of this chemistry for synthesizing biological polymers with alternative backbones - such as proline-rich sequences like collagen or foldamers based on beta or gamma amino acids - remains unclear. These critical points, alongside other relevant considerations, should be thoughtfully addressed in the revised manuscript to enhance its impact and clarity.

Author's Response to Peer Review Comments:

Reviewer(s)' Comments to Author:

Reviewer: 1

Recommendation: Publish in ACS Central Science after minor revisions noted.

Comments:

The study titled “Thioesters support efficient protein biosynthesis by the ribosome” by Alexandra Kent et al explores the use of thioesters in incorporating several natural and non-natural amino acids into proteins by ribosomal synthesis. They replaced oxo-esters with thioesters in acyltRNA substrates and used E coli ribosomal machinery to evaluate their substrate specificity in generating oligopeptides during in vitro translation. For this purpose, they have synthesized 3’thio-3’-deoxyadenosine triphosphate and showed that it supports all the 3 steps in protein translation: formation of 3’-thio-tRNA using a CCA-adding enzyme, aminoacylation of 3’-thiotRNA using enzymes/flexizymes and peptide bond formation using ribosome. Despite differences from oxo-esters in bond lengths, hydrogen bonding capacity and electrophilicity, efficient translation using thioester-tRNA substrates were observed. This suggests that changes in hydrogen bonding potential are not disruptive enough to inhibit translation. This work opens up the possibility of employing thioesters to incorporate natural and non-natural amino acids by the translational machinery for sequence-defined polypeptide synthesis. Overall, the work is well planned and well-executed, experiments were carefully performed, and data was presented lucidly. This study could be recommended for publication in ACS Central Science after addressing a few minor comments.

We appreciate the positive feedback that the reviewer has expressed, and we address the comments and observations below.

A few minor observations are:

1. Although the 3’-thio tRNAs acylated with various amino acids were recognized and utilized by the E. coli ribosomal machinery to form oligopeptides in comparable yields as of native oxoester tRNAs, I was wondering whether it affects the kinetics of the translation reaction? Authors may want to comment on this fact as the ribosomal protein synthesis is an incredibly optimized process with oxo-ester tRNAs and whether this O to S change has any impact on the overall efficiency of the reaction in synthesizing longer proteins.

This is an astute observation regarding the rate of the reaction and how that may affect translation. Although we hypothesize that having a thioester rather than an ester linking the amino acid to tRNA may increase the rate of peptide bond formation in some cases based on the physicochemical properties of esters and thioesters, we cannot with certainty assert what that difference may be without direct measurement. We think these measurements would be an interesting follow-up study to the proof-of-principle work presented here. Additionally, although there may be a difference in rate between ester and thioesters, peptide bond formation is not the rate limiting step for translation using natural amino acids. Rather, it is tRNA accommodation during mRNA decoding. For unnatural monomers where peptide bond formation may become the limiting step, a thioester may be critical for peptide production and even bond formation.

Because we employ the thioester linkage to replace the natural ester linkage for incorporation of one amino acid in the peptide, we don’t expect that the replacement would have a significant

impact on translation of longer peptides. We have added additional text to the discussion addressing the differences in rate.

2. Please include ppm errors in the mass spectrums provided in the text.

The Figure 4 caption text has been modified to include ppm errors.

3. Is there any experimental or theoretical evidence for the proposed role EF-Tu in preferential binding to 3'-thioester during translation?

From structural evidence, it is known that EF-Tu binds 3'-aminoacylated tRNAs. We make this point more explicitly in the revised Discussion. Our evidence for the role of EF-Tu in substrate delivery is presently indirect. But by analogy to peptide bond formation on the ribosome, although the 2'-ester is favored over the 3'-thioester at equilibrium, the exchange between the 2' and 3' positions is likely sufficient to allow EF-Tu to bind the 3'-thioester form for alpha-amino acids. We raise this hypothesis in the Discussion for completeness. However, a more detailed analysis of EF-Tu binding would need to be performed such as that reported in *Cruz-Navarrete, F. A.; et al. ACS Cent. Sci. 2024, 10(6), 1262-1275* (ref. 56), an avenue we are interested in pursuing.

Thank you

Additional Questions:

Quality of experimental data, technical rigor: Top 5%

Significance to chemistry researchers in this and related fields: Top 5%

Broad interest to other researchers: High

Novelty: High

Is this research study suitable for media coverage or a First Reactions (a News & Views piece in the journal)?: Yes

Reviewer: 2

Recommendation: Publish in ACS Central Science after minor revisions noted.

Comments:

This is an excellent paper from Cate and co-workers that describes the synthesis 3'-thio3'deoxy-adenosine and incorporation into tRNAs for ribosomal translation. IN a series of welllaid out experiments, they convince this reviewer that the ribosomal synthesis using these 3'thio-tRNAs proceeds via intermediacy of the corresponding tRNA-thioester. The work opens doors and certainly begs additional questions, but it is substantive and complete in its demonstration of the ability of these unnatural sugars to mediate ribosomal translation. This work would make an appropriate addition to the pages of ACS Central Science. I have only a few minor concerns:

We appreciate the positive feedback from the reviewer and address the concerns below.

1. Yields for the 3'-thio-3'deoxy-adenosine should be presented in Figure 1d (or below) in the main text, not just SI. It would also be helpful to report the authors yield for compound 2 from 1 (according to the literature protocol.

We thank the reviewer for pointing out this missing information. The manuscript has been modified to include the yields in the Figure 1 caption. We have included the yield for compound 2 as well.

2. The authors seem to eschew the more quantitative translation assays that have recently been reported by Genentech (Chan, A. I. et al. ACS Chem. Biol. 18, 81–90, 2023) and, more recently, Matt Hartmans (Kerestesy, G. N., et al., ACS Synth. Biol. 13, 119–128, 2024) group. This reviewer would prefer if the authors results were accompanied by such assay data. In lieu of this, please add a rationale for the choice of MS over these more quantitative assays. Additionally, please include all MS parameters used for analysis here (injection volume, gas flow and temp, nebulizer pressure, VCap voltage, fragmentor voltage, and skimmer voltage). This would help improve reproducibility of these types of results.

We agree with the reviewer that there are alternative methods to monitor non-canonical amino acid incorporation. Indeed, we regularly employ the HiBit assay described by the Genentech publication the reviewer cited. While we agree that the bioluminescence and fluorescence based assays can provide insight into non-canonical amino acid incorporation, we wanted to be absolutely sure that the product we obtained in these reactions was the result of incorporation of the unnatural amino acid and was not the result of nonspecific stop codon readthrough. This was a critical step in ensuring the translated peptide made use of the thioester linked noncanonical monomers and for this reason we focused our efforts on acquiring LC-MS data. In our hands, we have seen that stop codon readthrough can result in false positive signals from the HiBit assay. We also note that because we are interested in the ratio of incorporation of a canonical or non-canonical amino acid from an ester vs. a thioester, we did not need to calculate the absolute yield of the peptide by way of a standard curve. In the future, we agree fluorescence or bioluminescence based assays could be useful in follow up studies to screen incorporation of a wider variety of different non-canonical substrates, since these assays have utility for high throughput experiments.

Additional detail has been included in the Supporting Information on the parameters used during LC-MS analysis of the peptides to aid in reproducibility.

3. In Figure 4, it would be more helpful to see the integrated EIC scale represented as counts (absolute units) as they are in the SI. This does not change intensity of the overlays and authors can keep the ratio bars on the side, but allows the reader to see differences in the translation efficiencies between the substrates (e.g. the alpha-hydroxy substrate).

We appreciate that the reviewer has highlighted a point that we considered and discussed extensively. We note that all the EIC scales for all replicates are reported in the Supporting Information. We decided not to use this information in the main figures, because although the ratio of peptide produced remains constant between experiments performed in the same batch (as all the ester vs. thioester experiments were) the EIC scale can vary between replicates and between different peptide sequences. In the case of replicates, this is a result of typical experimental error introduced primarily in downstream sample preparation steps, i.e. the antiFLAG affinity purification step. For peptides with different identities, there can be a difference in ionization efficiency. Thus a direct comparison between the different peptides produced is not quantitative and does not provide additional experimental insight in the main figures of the manuscript.

4. At the end of the manuscript, the authors allude to the idea that these 3'-thio-tRNAs might enable additional nucleophiles. Several additional examples in Figure 4 would help solidify the results. Since the authors are already using Flexizyme, 1-2 more alpha-hydroxy acids could help prove the pattern. Also, an N-methyl amino acid would be significantly appreciated here and would go a long way in cementing interest in this paper for the broader community. The present substrate scope is far too limited.

We agree with the reviewer that this is the logical next step to the project and one we are very interested in pursuing. However, the intent of this work is to primarily provide a proof of concept that thioesters can be used in translation to incorporate non-canonical amino acids and monomers. We believe that the data currently presented are sufficient to demonstrate that intent. We changed the wording at the end of the manuscript to indicate that these are ideas that can now be explored, now that we know protein biosynthesis can utilize 3'-thio-tRNAs.

Additional Questions:

Quality of experimental data, technical rigor: High

Significance to chemistry researchers in this and related fields: High

Broad interest to other researchers: High

Novelty: High

Is this research study suitable for media coverage or a First Reactions (a News & Views piece in the journal)?: No

Reviewer: 3

Recommendation: Reconsider after major revisions noted.

Comments:

Thioesters are more reactive than oxygen esters, resulting in faster hydrolysis rates under standard biochemical conditions (neutral pH, 37°C, physiological buffer). Their hydrolysis rates vary depending on their structure and environmental factors. For instance, certain alkylthioesters have half-lives of approximately 40 hours at pH 7 and room temperature, though this rate can shift with changes in temperature or pH. In contrast, peptide bonds (amides), chosen by nature as the backbone for proteins, are significantly more stable, with estimated half-lives of around 1,000 years under the same conditions. This inherent reactivity of thioesters rationalizes their widespread role as intermediates in metabolic processes, rather than as chemical linkages in biological polymers.

Nonetheless, thioesters such as thiol esters of amino acids, play a significant role in peptide biosynthesis as intermediates, mimicking aspects of canonical translation. They are speculated to be used as intermediates in non-canonical protein synthesis, even by ribosomes under controlled conditions. This is possible because thioesters are highly reactive acyl donors, owing to their polarizable sulfur atom, which weakens the bond between the carbonyl carbon (which is more electrophilic) and the leaving group—a feature that underpins their role in metabolism. Non-ribosomal peptide synthetases (NRPSs) is the best documented example of efficient use thioesters as intermediates in peptide biosynthesis. This open-up the possibilities to transfer this chemistry to ribosomal peptide and protein synthesis, leveraging their efficient coupling capabilities. Engineered aminoacyl-tRNA synthetases could theoretically activate thioester derivatives to load non-canonical amino acids (ncAAs) onto tRNAs.

The activation of amino acids in both ribosomal and NRPS systems involves the formation of aminoacyl-adenylates. However, the transfer mechanism differs in NRPSs, the aminoacyladenylate is transferred to the thiol group of a peptidyl carrier protein, forming a thioester linkage. In contrast, ribosomal synthesis involves transfer to tRNA, resulting in a classical ester linkage.

In this context, the manuscript of Kent et al., reports an interesting innovation that demonstrate that tRNAs acylated with a thioester, instead of the traditional ester linkage, to both cAAa and ncAAs can be utilized by the existing translation machinery for the synthesis of sequencedefined polymers. In particular, the authors synthesized 3'-thio-3'-deoxyadenosine triphosphate from xylose and demonstrated its incorporation into truncated tRNAs via the E. coli CCA-adding enzyme, enabling the generation of 3'-thio-tRNAs acylated with α- and non-α-amino acids. These 3'-thio-tRNAs were successfully utilized by wild-type E. coli ribosomes during in vitro translation.

In particular, the 3'-OH on the 3'-terminal adenosines of a tRNA were replaced with a 3'-SH, and its performance was evaluated in tRNA extension, aminoacylation, and ribosome-mediated peptide bond formation. All three reactions were fully supported by thioesters, with yields comparable to those of oxo-ester tRNAs. The 3'-thio-ATP is a substrate for E. coli tRNA nucleotidyltransferase, a CCA-adding enzyme, to add 3'-thio-3'-deoxyadenosine to the 3'-end of truncated tRNAs generated using in vitro transcription.

The central novelty of the paper is the use of native chemical ligation as evidence for the presence of thioesters. The authors investigated the dynamic interconversion between 3'thioesters and 2'-oxo-esters on acylated tRNAs, finding that while the 2'-oxo-ester is thermodynamically favored, transient formation of the 3'-thioester occurs under kinetic control. This transient state was confirmed through native chemical ligation, resulting in dipeptide formation, despite the predominance of the 2'-oxo-ester observed via NMR. Translation efficiency in the ribosomal PTC was assessed using tRNAs with thioester or oxoester linkages, revealing significantly higher peptide yields with oxoester-linked tRNAs compared to thioesterlinked tRNAs, expectedly demonstrating the superior compatibility of oxoesters in translation. In particular, in vitro ribosomal translation was shown to tolerate a single O-to-S substitution at the P-site tRNA when incorporating non-canonical α-amino acids, with oxoester-linked tRNAs producing higher yields of peptides containing successive BocLys residues. However, when incorporating a β2-hydroxy acid monomer, yields were equivalent regardless of the linkage type, though overall efficiency was reduced compared to incorporating two BocLys monomers. In conclusion, thioesters were explored as replacements for esters in aminoacyl-tRNAs, leveraging their increased electrophilicity and potential role in peptide (and possibly fatty acid) formation. The key contribution of this approach lies in demonstrating that ribosomal translation machinery can tolerate the substitution of the ester bond, thereby facilitating the incorporation of less nucleophilic ncAAs via thioesters.

Technically, the chemical syntheses and aminoacylations of 3'-thio-tRNAs were thoroughly documented and performed with appropriate citation of relevant literature. However, the manuscript does not clearly delineate the novelty or advantages of this method compared to established approaches, such as native chemical ligation, expressed protein ligation, or the use of flexizymes. Furthermore, while evolutionary implications are discussed, the practical applications of this approach are insufficiently addressed, with only broad, unspecific statements regarding utility in synthetic biology.

Finally, the relevance of this chemistry for synthesizing biological polymers with alternative backbones - such as proline-rich sequences like collagen or foldamers based on beta or gamma amino acids - remains unclear. These critical points, alongside other relevant considerations, should be thoughtfully addressed in the revised manuscript to enhance its impact and clarity.

We thank the reviewer for the interest in our work. Some points that were raised were also made by other reviewers and have been addressed in the discussion. We note that other methods such as native chemical ligation and expressed protein ligation are inherently posttranslational events. By contrast, our approach allows for direct incorporation of monomers by the ribosome, which in the future may allow for multiple monomer incorporation events. We also show in Figure 3 that 3'-thio-tRNAs are widely compatible with aminoacyl-tRNA synthetases, flexizymes, and even thioester

exchange. We discuss these advantages in the third paragraph of the Discussion. Although we intend to pursue the synthetic biology angle presented in this manuscript further to determine how this new method could allow for more efficient incorporation of some recalcitrant monomers such as beta amino acids, this manuscript serves primarily as a proof of concept that thioesters can be used by the full suite of translation machinery. We have modified the last sentences of the Discussion to make this point more explicitly.

Additional Questions:

Quality of experimental data, technical rigor: High

Significance to chemistry researchers in this and related fields: High

Broad interest to other researchers: High

Novelty: High

Is this research study suitable for media coverage or a First Reactions (a News & Views piece in the journal)?: Yes
